# Multi-omics analysis of m^6^A modification-related patterns based on m^6^A regulators and tumor microenvironment infiltration in lung adenocarcinoma

**DOI:** 10.1038/s41598-021-00272-z

**Published:** 2021-10-22

**Authors:** Xincheng Wu, Zhengping Bai

**Affiliations:** 1 Hunan University of Chinese Medicine, 300 Xueshi Rd., Yuelu District, 410208 Changsha China; 2 Hunan Academy of Chinese Medicine, 58 Lushan Rd., Yuelu District, 410006 Changsha China

**Keywords:** Cancer, Genetics, Immunology, Molecular medicine

## Abstract

Epigenetic modifications, especially N^6^-methyladenosine (m^6^A) modification, play a key role in tumor microenvironment (TME) infiltration. However, the regulatory role of m^6^A modification in the TME of lung adenocarcinoma (LUAD) remains unclear. A total of 2506 patients with LUAD were included in the analysis and divided into different groups according to distinct m^6^A modification-related patterns based on 23 m^6^A regulators. A comprehensive analysis was performed to explore TME infiltration in different m^6^A modification-related patterns. Principal component analysis was performed to obtain the m^6^Ascore and to quantify m^6^A modification-related patterns in different individuals. Three distinct m^6^A modification-related patterns were identified by 23 m^6^A regulators. The pathway enrichment analysis showed that m^6^Acluster-A was associated with immune activation; m^6^Acluster-B was associated with carcinogenic activation; m^6^Acluster-C was prominently related to substance metabolism. M^6^Acluster-A was remarkably rich in TME-infiltrating immune cells and patients with this pattern showed a survival advantage. The m^6^Ascore could predict TME infiltration, tumor mutation burden (TMB), the effect of tumor immunotherapy, and the prognosis of patients in LUAD. High m^6^Ascore was characterized by increased TME infiltration, reduced TMB, and survival advantage. Patients with a high m^6^Ascore exhibited significantly improved clinical response to anti-cytotoxic T lymphocyte antigen-4 (anti-CTLA4) immunotherapy. This study explored the regulatory mechanisms of TME infiltration in LUAD. The comprehensive analysis of m^6^A modification-related patterns may contribute to the development of individualized immunotherapy and the improvement of the overall effectiveness of immunotherapy for LUAD patients.

## Introduction

Lung cancer (LC), a fatal malignancy, has become a leading cause of malignant tumor-related death worldwide^1^. The 5-year survival rate of LC at a localized stage, regional stage, and distant stage is 54%, 26%, and 4%, respectively^2^. The prognosis of LC is poor, because approximately 57% of LC patients are diagnosed at the distant stage^3,4^. LC can be histologically classified into non-small-cell lung cancer (NSCLC) and small-cell lung cancer (SCLC). NSCLC accounts for ~ 85% of all LC cases^5,6^. Primary treatments for LUAD include surgery, chemotherapy, and radiotherapy. However, the prognosis of advanced LUAD remains poor due to limited treatment efficacy, which requires the development of new therapeutic targets and treatments.

Tumor growth and spread depend not only on tumor cell characteristics but also on the interaction between tumor cells and tumor microenvironment (TME), a cellular environment where tumors or cancer stem cells exist^7–9^. TME consists of multiple components, including infiltrating immune cells^10^. TME plays a pivotal role in tumorigenesis, and its heterogeneity may lead to multiple dimensions in the therapeutic response and prognosis of patients^11–14^. Immunotherapy using immune checkpoint inhibitors is based on TME cell infiltration and has become a promising treatment strategy for cancer patients, including LC^15^. The drugs that are widely used in LC immunotherapy include medications targeting programmed cell death protein 1 (PD-1) and cytotoxic T lymphocyte antigen-4 (CTLA4), such as Nivolumab and Ipilimumab^7^. Although a small proportion of cancer patients respond well to immunotherapy, the majority of them experience minimal or no clinical benefits^16^. In addition, the clinical application of immunotherapies is limited by their toxicity profiles^17–19^. Future investigations on the diversity and complexity of TME may elucidate the effects of TME on tumor progression, immune escape, and immunotherapeutic response. Personalized immunotherapy may also be provided for LUAD patients based on the tumor-immune phenotypes identified by the analysis of TME heterogeneity.

N^6^-methyladenosine (m^6^A) modification, referring to methylation at the sixth N atom of adenine, is the third layer of epigenetic modification. It is the most common post-transcriptional modification on mRNA, long non-coding RNA, as well as microRNA^20–24^. The m^6^A modification on RNA is a dynamic process involving binding proteins (“readers”), demethylases (“erasers”), and methyltransferases (“writers”), all of which are termed m^6^A regulators^25^. The formation of m^6^A is catalyzed by methyltransferases. The binding proteins recognize and bind to m^6^A methylation sites, and the methyl codes of target RNAs are removed by demethylases^26,27^. M^6^A modification is implicated in RNA transcription, processing, splicing, degradation, and translation^28,29^. Aberrant m^6^A modification is closely associated with the onset and progression of tumors^22,23^. The m^6^A regulators also play critical roles in tumorigenesis^22,30,31^. Jin et al. found that ALKBH5 inhibited the expression of YAP via targeting the remover of m^6^A modification. In addition, YAP was negatively associated with the proliferation, invasion, migration, and epithelial-to-mesenchymal transition of NSCLC cells^32^. Taken together, m^6^A regulators-mediated m^6^A modification is implicated in the occurrence, progression, and prognosis of cancers, including LC.

Recent evidence has revealed that m^6^A modification is closely related to TME infiltration of immune cells, which affects immunotherapeutic responses^33^. Wang et al. found that the suppression of m^6^A modification sensitized tumor cells to immunotherapy by altering TME and the recruitment of CD^8+^ tumor-infiltrating lymphocytes. In addition, the inhibition of m^6^A regulators improved the effectiveness of immunotherapies against colorectal cancer^34^. Therefore, a comprehensive analysis of the correlation between TME and m^6^A regulators-mediated m^6^A modification may further elucidate the pathogenic mechanisms of LUAD and provide scientific support for the development of novel immunotherapy. In this study, the genomic data of LUAD samples were obtained from the public databases, and then used for comprehensive analyses of m^6^A modification-related patterns and the correlation between m^6^A regulators and TME infiltration. Three distinct m^6^A modification-related patterns with distinct degrees of TME cell infiltration were identified, suggesting that m^6^A modification played an indispensable role in the formation of TME. Moreover, a scoring system was developed to quantify m^6^A modification-related patterns in different individuals. This study may provide insights into a better understanding of TME-related regulatory mechanisms in LUAD and the optimization of personalized immunotherapy for LUAD patients.

## Results

### Genetic variation of m^6^A regulators in LUAD

**Figure 1:**
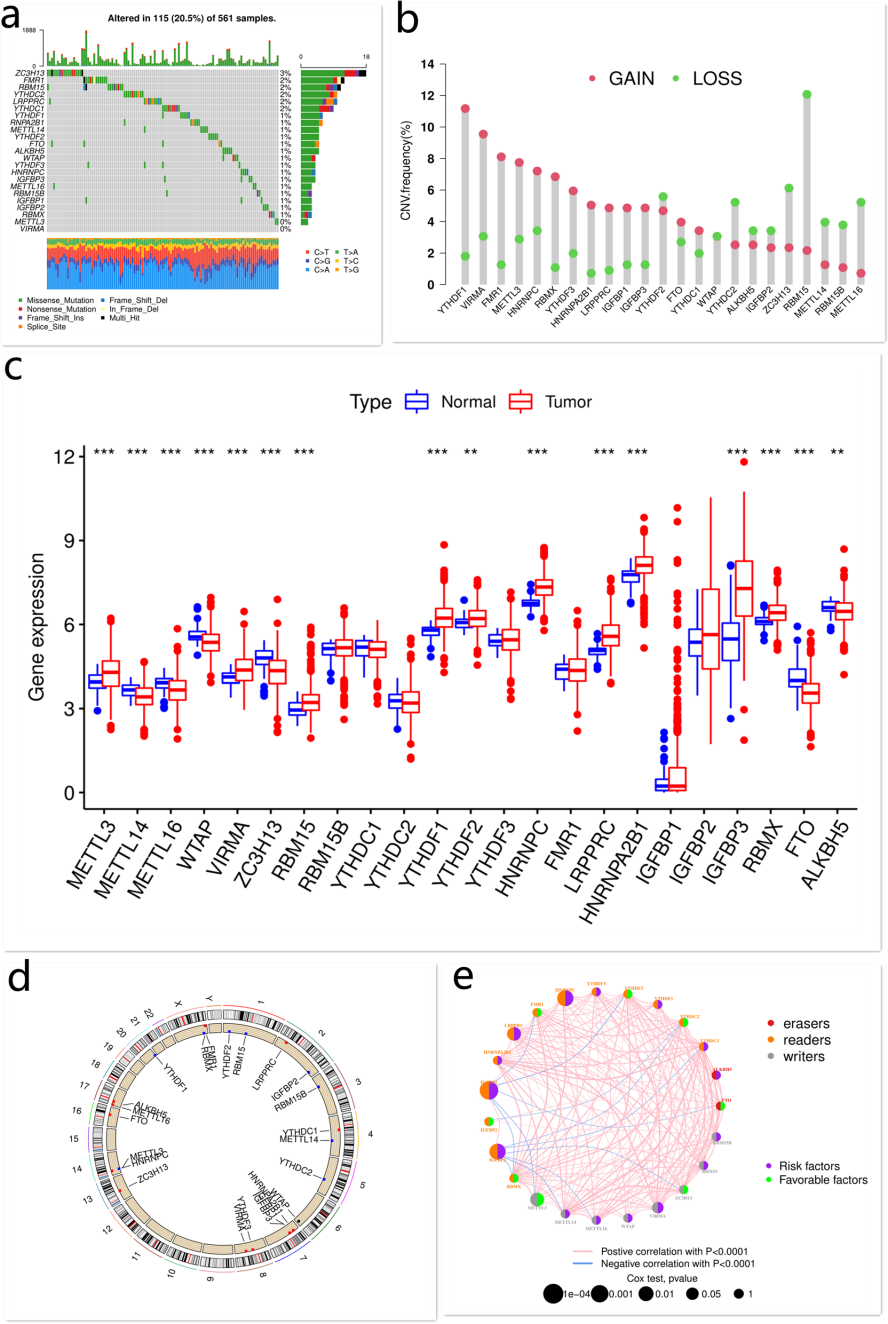
The expression, mutation characteristics, and relation of m^6^A regulators in LUAD. The mutation frequency of m^6^A regulators in LUAD (**a**). The CNV frequency of m^6^A regulators in LUAD. Blue dots indicate deletion frequency, while red dots indicate amplification frequency (**b**). The expression of m^6^A regulators in LUAD (**c**). The location of CNV alteration of 23 m^6^A regulators on chromosomes (**d**). The relation of m^6^A regulators in LUAD. The circle size indicates the survival impact of each m^6^A regulator. The lines connecting m^6^A regulators indicate their relations. The thickness of line indicates the strength of the relation. The red lines indicate positive relations, while the blue lines indicate negative relations (**e**). This figure is created using the R (version 4.0.3) (https://www.r-project.org/).

A total of 23 m^6^A regulators, including 13 “readers”, 8 “writers”, and 2 “erasers”, were identified (Table 1). The incidence of CNV and somatic mutations of 23 m^6^A regulators in LUAD was summarized. The mutation of m^6^A regulators was observed in 115 out of 561 samples, with a frequency of 20.5%. ZC3H13 exhibited the highest mutation frequency among all m^6^A regulators (Fig. 1a). The CNV analysis was performed to show the CNV frequency of 23 regulators. Among them, YTHDF1, VIRMA, FMR1, METTL3, HNRNPC, RBMX, YTHDF3, HNRNPA2B1, LRPPRC, IGFBP1, IGFBP3, FTO, and YTHDC1 showed a trend of amplification, while YTHDF2, WTAP, YTHDC2, ALKBH5, IGFBP2, ZC3H13, RBM15, METTL14, RBM15B, and METTL16 had a high frequency of deletion (Fig. 1b). We then measured the mRNA levels of these regulators in LUAD and normal lung tissues. Compared with normal tissues, the expressions of METTL3, VIRMA, RBM15, YTHDF1, YTHDF2, HNRNPC, LRPPRC, HNRNPA2B1, IGFBP3, and RBMX were markedly elevated in LUAD tissues, and vice versa (e.g. METTL14, METTL16, WTAP, ZC3H13, FTO, and ALKBH5) (Fig. 1c). The location of CNV alteration of m^6^A regulators on chromosomes is shown in Fig. 1d. These findings showed high genetic and expressional heterogeneity of m^6^A regulators between LUAD and normal lung tissues, suggesting that aberrant expression of m^6^A regulators may play a critical role in the occurrence and development of LUAD.

**Table 1:** N^6^-methyladenosine (m^6^A) regulators.

Regulator	Full name	Category	Regulator	Full name	Category
METTL3	Methyltransferase-like protein 3	Writer^**1**^	YTHDF3	YTH m^6^A RNA-binding protein 3	Reader
METTL14	Methyltransferase-like protein 14	Writer	HNRNPC	Heterogeneous nuclear ribonucleoprotein C	Reader
METTL16	Methyltransferase-like protein 16	Writer	FMR1	Fragile X mental retardation protein	Reader
WTAP	Wilms tumor 1-associated protein	Writer	LRPPRC	Leucine-rich PPR-motif-containing protein	Reader
VIRMA	Vir-like m^6^A methyltransferase associated protein	Writer	HNRNPA2B1	Heterogeneous nuclear ribonucleoprotein A2B1	Reader
ZC3H13	zinc finger CCCH domain-containing protein 13	Writer	IGFBP1	Insulin-like growth factor binding protein 1	Reader
RBM15	RNA-binding motif protein 15	Writer	IGFBP2	Insulin-like growth factor binding protein 2	Reader
RBM15B	RNA binding motif protein 15B	Writer	IGFBP3	Insulin-like growth factor binding protein 3	Reader
YTHDC1	YTH domain-containing 1	Reader^**2**^	RBMX	X-linked RNA-binding motif protein	Reader
YTHDC2	YTH domain-containing 2	Reader	FTO	Fat mass and obesity-associated protein	Eraser^**3**^
YTHDF1	YTH m^6^A RNA-binding protein 1	Reader	ALKBH5	Alk B homologue 5	Eraser
YTHDF2	YTH m^6^A RNA-binding protein 2	Reader			

^1^The m^6^A methyltransferases catalyze the formation of m^6^A as m^6^A writers.

^2^The m^6^A demethylases remove the methyl codes from target RNAs as m^6^A erasers.

^3^The m^6^A-binding proteins recognize and bind to the m^6^A methylation sites in RNA as m^6^A readers.

### M^6^A modification-related patterns mediated by 23 m^6^A regulators

Three GEO datasets (GSE68465, GSE68571, and GSE72094) with available clinical information and overall survival (OS) data were integrated into one meta-cohort. A m^6^A regulator network was generated to depict the landscape of m^6^A regulator interactions and their prognostic value for LUAD (Fig. 1e; Supplementary Fig. S1). The m^6^A regulators in the same functional category were significantly correlated. We also observed significant correlations among “readers”, “writers”, and “erasers”. HNRNPC, YTHDF3, YTHDF1, YTHDC1, ALKBH5, RBM15B, RBM15, VIRMA, WTAP, METTL16, METTL14, IGFBP3, IGFBP1, HNRNPA2B1, and LRPPRC were the risk factors for LUAD. In addition, IGFBP1, HNRNPC, IGFBP3, and LRPPRC were significantly associated with the prognosis of LUAD patients (Fig. 1e, Supplementary Fig. S1). Considering that some m^6^A regulators (e.g. ZC3H13, FMR1, RBM15, YTHDC2, LRPPRC, and YTHDC1) had a relatively high mutation frequency, we compared the expression of mutant and wild-type m^6^A regulators. Compared with mutant-type tumors, the levels of WTAP, IGFBP2, and IGFBP1 in tumors with normal FMR1, LRPPRC, and YTHDC2 expression, respectively, were upregulated. Compared with wild-type tumors, the levels of LRPPRC and HNRNPA2B1 were upregulated in RBM15 and YTHDC1-mutant tumors, respectively (Supplementary Fig. S2a–e). The above data suggested that the cross-talk among these regulators plays a critical role in the occurrence, development, and prognosis of LUAD.

**Figure 2:**
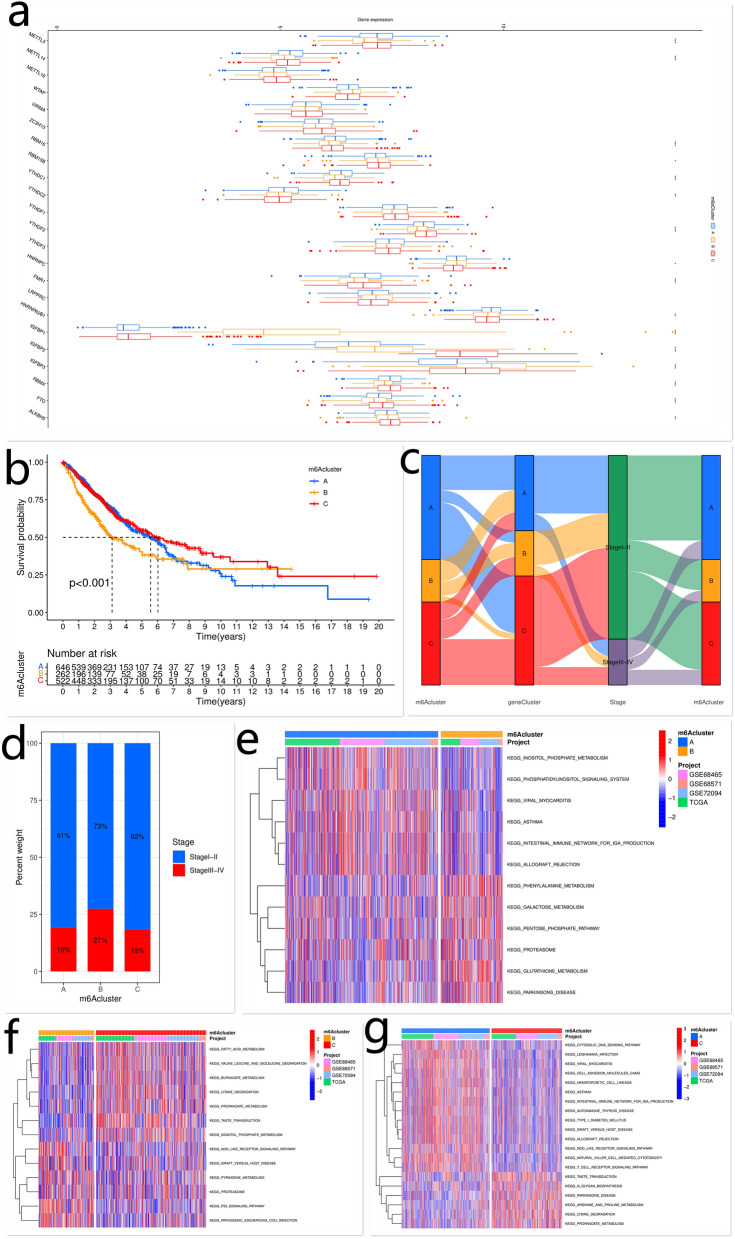
The expression of m6A regulators, clinical characteristics of LUAD patients, and biological processes in different m6A modification-related patterns. The expression of m^6^A regulators in different m^6^A modification-related patterns (**a**). The Kaplan–Meier curves of the OS of LUAD patients with different m^6^A modification-related patterns (**b**). The relationships among m^6^Acluster, m^6^A genecluster, and stage were visualized using alluvial diagram (**c**). The percentage of patients with stage I, II/III, and IV LUAD in each m^6^Acluster (**d**). The biological processes in different m^6^A modification-related patterns. Red indicates activation, while blue indicates inhibition (**e**, **f**, **g**). This figure is created using the R (version 4.0.3) (https://www.r-project.org/).

Three m^6^A modification-related patterns based on the expression of 23 m^6^A regulators were identified and termed m^6^Acluster-A–C, respectively (Supplementary Fig. S2f). Patients were then classified into different groups according to their m^6^A modification-related patterns (m^6^Acluster-A: n = 646; m^6^Acluster-B: n = 262; m^6^Acluster-C: n = 522). M^6^Acluster A was characterized by the upregulation of METTL14, RBM15, YTHDC1, YTHDC2, FMR1, and HNRNPA2B1; m^6^Acluster B showed upregulated expression of IGFBP1 and IGFBP3; m^6^Acluster C exhibited significantly increased expression of RBM15B, YTHDF2, IGFBP2, FTO, and ALKBH5 (Fig. 2a). Furthermore, a prominent survival advantage was observed in patients with m^6^Acluster-A and -C, while the worst survival was observed in those with m^6^Acluster-B (Fig. 2b). We also noticed that the percentage of patients with stage III and IV LUAD in m^6^Acluster-B was higher than that in m^6^Acluster-A and -C (Fig. 2c,d, Supplementary Table S1). Therefore, patients with m^6^Acluster-B had the worst survival and most advanced tumor stages compared to those with m^6^Acluster-A and C. Further analysis showed that the transcriptional profile of m^6^Acluster-B was significantly distinct from that of m^6^Acluster-A and -C, which was consistent with the clinical features (e.g. clinical survival and tumor stage) of patients with different m^6^A modification-related patterns (Supplementary Fig. S3a). The above results showed that m^6^A modification played a crucial role in the progression and prognosis of LUAD.

### Characteristics of TME infiltration in different m^6^A modification-related patterns

**Figure 3:**
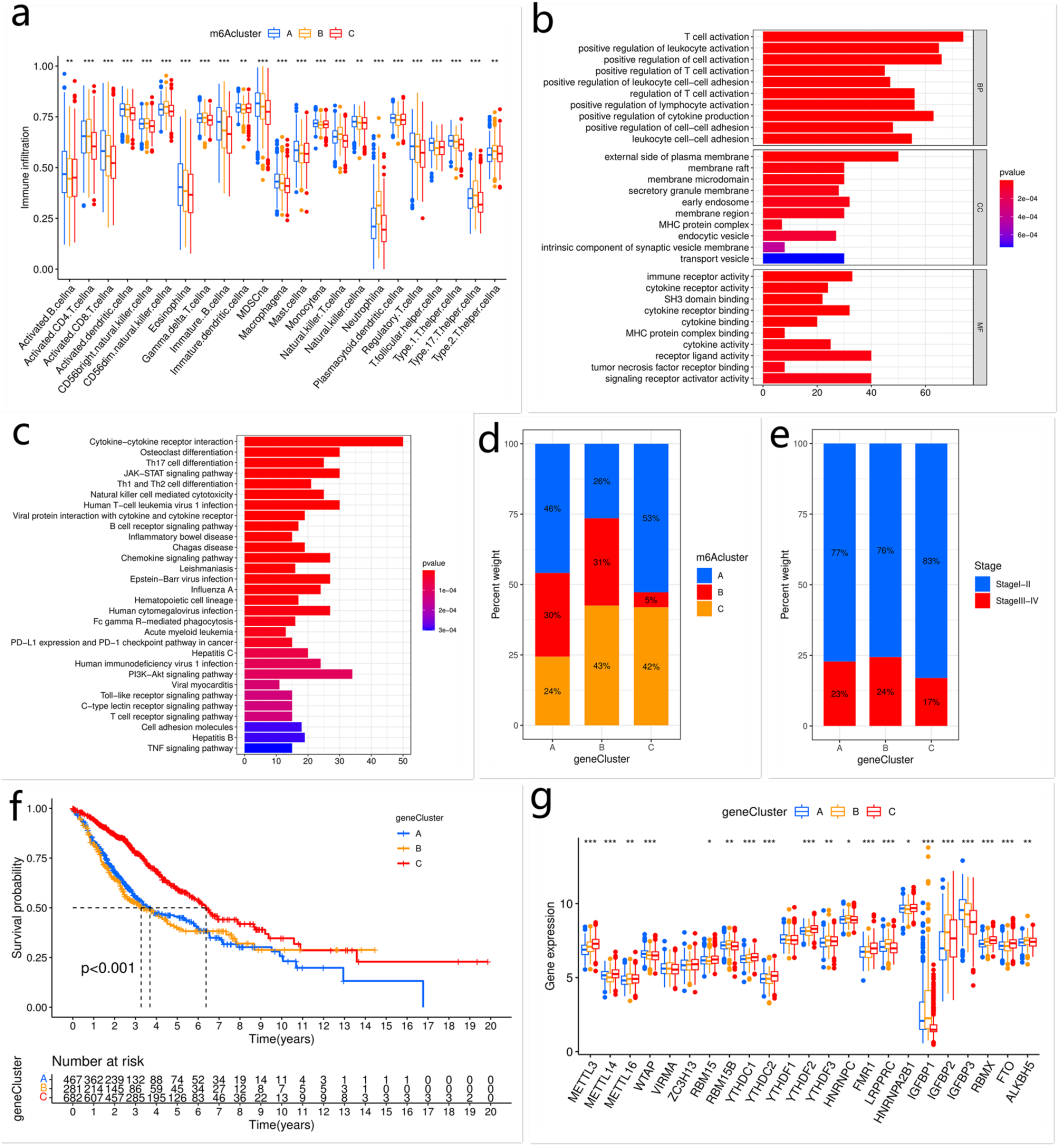
TME-infiltrating immune cells in different m6A modification-related patterns; biological processes and clinical characteristics of LUAD patients in different m6A geneclusters. Characteristics of TME-infiltrating cells in different m^6^A modification-related patterns (**a**). GO and KEGG enrichment analyses of m^6^A phenotype-related DEGs (**b**, **c**). The percentage of patients with m^6^Acluster-A/-B/-C in each m^6^A geneCluster (**d**). The percentage of patients with stage I, II/III, and IV LUAD in each m^6^A geneCluster (**e**). The Kaplan–Meier curves of the OS of LUAD patients in different m^6^A geneClusters (**f**). The expression of m^6^A regulators in distinct m^6^A geneclusters (**g**). This figure is created using the R (version 4.0.3) (https://www.r-project.org/).

The GSVA enrichment analysis was performed to explore the biological behaviors of different m^6^A modification-related patterns. In m^6^Acluster-A, the enriched pathways were associated with immune activation, such as cell adhesion molecules, T cell receptor signaling pathway, and natural killer cell-mediated cytotoxicity. Patients with m^6^Acluster-B presented poor survival and the enriched pathways were associated with carcinogenic activation, including NOD-like receptor signaling pathway and p53 signaling pathway. M^6^Acluster-C was predominantly related to sugar, lipid, and protein metabolism, such as fatty acid metabolism, N glycan biosynthesis, valine leucine and isoleucine degradation, and the TCA cycle (Fig. 2e–g, Tables 2, 3, 4). Surprisingly, m^6^Acluster-A was remarkably rich in TME-infiltrating immune cells, including T follicular helper cells, eosinophils, activated B cells, activated CD8 T cells, activated dendritic cells, immature B cells, mast cells, natural killer cells, macrophages, monocytes, plasmacytoid dendritic cells, myeloid-derived suppressor cells, and Type 1 T helper cells (Fig. 3a). Patients with m^6^Acluster-A also showed a survival advantage.

**Table 2:** The activation states of biological pathways in distinct m^6^A modification patterns by GSVA enrichment analysis (A vs. B).

Pathway	logFC	AveExpr	t	*p *value	Adj. *p* val.
Glutathione metabolism	0.165641	− 0.02904	8.208943	7.21E−16	1.14E−13
Inositol phosphate metabolism	− 0.12199	− 0.00484	− 7.71161	3.12E−14	2.47E−12
Phosphatidylinositol signaling system	− 0.10869	0.002708	− 7.19802	1.24E−12	6.52E−11
Phenylalanine metabolism	0.138609	− 0.03086	6.801562	1.83E−11	7.21E−10
Asthma	− 0.1507	0.048538	− 6.3068	4.35E−10	8.60E−09
Parkinsons disease	0.127406	− 0.05194	6.105625	1.49E−09	2.35E−08
Viral myocarditis	− 0.10773	0.054209	− 5.85274	6.65E−09	9.55E−08
Galactose metabolism	0.101446	0.012333	5.52438	4.27E−08	4.08E−07
Pentose phosphate pathway	0.111034	− 0.0334	5.519075	4.39E−08	4.08E−07
Intestinal immune network for IGA production	− 0.10316	0.058451	− 3.9643	7.91E−05	0.000278
Allograft rejection	− 0.10911	0.068016	− 3.9402	8.74E−05	0.0003
Proteasome	0.103781	− 0.00538	3.913471	9.75E−05	0.000328

AveExpr: average expression; adj. *p*. val: adjust *p* value.

**Table 3:** The activation states of biological pathways in distinct m^6^A modification patterns by GSVA enrichment analysis (B vs. C).

Pathway	logFC	AveExpr	t	*p* value	Adj. *p* val.
Taste transduction	0.173708	0.023282	9.54949	1.48E−20	2.33E−18
Lysine degradation	0.176431	0.022864	8.486039	1.00E−16	7.91E−15
Valine leucine and isoleucine degradation	0.165508	0.00292	7.31964	5.96E−13	3.14E−11
P53 signaling pathway	− 0.10236	− 0.00466	− 6.907	9.97E−12	3.94E−10
Inositol phosphate metabolism	0.100877	− 0.02475	6.540024	1.08E−10	3.15E−09
Pathogenic Escherichia coli infection	− 0.11322	− 0.02392	− 6.52473	1.20E−10	3.15E−09
Propanoate metabolism	0.159995	0.012698	6.401749	2.59E−10	5.85E−09
Butanoate metabolism	0.136867	0.00647	6.275974	5.64E−10	1.09E−08
Nod like receptor signaling pathway	− 0.10518	− 0.0319	− 5.96341	3.68E−09	5.28E−08
Pyrimidine metabolism	− 0.10614	− 0.01397	− 5.65029	2.21E−08	2.91E−07
Fatty acid metabolism	0.122256	0.006	5.269864	1.75E−07	1.84E−06
Proteasome	− 0.11058	− 0.00488	− 4.13551	3.91E−05	0.000199
Graft versus host disease	− 0.10532	− 0.06268	− 3.57728	0.000368	0.00132

AveExpr: Average expression, adj.P.Val: adjust P Value.

**Table 4:** The activation states of biological pathways in distinct m^6^A modification patterns by GSVA enrichment analysis (A vs. C).

Pathway	logFC	AveExpr	t	P.Value	adj.P.Val
Natural killer cell mediated cytotoxicity	− 0.13661	0.013288	− 10.3918	2.70E−24	4.27E−22
Lysine degradation	0.151444	− 0.00211	9.616063	3.77E−21	2.98E−19
Arginine and proline metabolism	0.111808	− 0.01228	9.110563	3.28E−19	1.73E−17
Graft versus host disease	− 0.19841	0.012295	− 8.72385	8.68E−18	2.74E−16
Allograft rejection	− 0.18999	0.015054	− 8.69579	1.10E−17	2.89E−16
Leishmania infection	− 0.13862	0.012236	− 8.5997	2.42E−17	5.47E−16
Viral myocarditis	− 0.1303	0.027342	− 8.43941	8.94E−17	1.47E−15
Nod like receptor signaling pathway	− 0.11469	− 0.00344	− 8.43444	9.31E−17	1.47E−15
T cell receptor signaling pathway	− 0.10918	0.008681	− 8.16473	7.98E−16	9.70E−15
Cytosolic DNA sensing pathway	− 0.11416	0.00249	− 8.05372	1.90E−15	2.14E−14
Type I diabetes mellitus	− 0.14067	0.005598	− 7.76164	1.77E−14	1.74E−13
Asthma	− 0.14966	0.025435	− 7.64815	4.12E−14	3.83E−13
Taste transduction	0.105965	0.022686	7.616596	5.21E−14	4.33E−13
Intestinal immune network for IGA production	− 0.15357	0.019942	− 7.40868	2.38E−13	1.79E−12
Parkinsons disease	0.116117	− 0.03703	7.038527	3.24E−12	2.13E−11
Autoimmune thyroid disease	− 0.12703	0.016094	− 6.86282	1.07E−11	6.78E−11
Hematopoietic cell lineage	− 0.10746	0.017027	− 6.79882	1.65E−11	1.00E−10
N-glycan biosynthesis	0.101026	− 0.01521	6.612347	5.65E−11	3.30E−10
Propanoate metabolism	0.12416	− 0.00262	6.549583	8.49E−11	4.79E−10
Cell adhesion molecules cams	− 0.10653	0.022996	− 6.53179	9.52E−11	5.19E−10
Primary immunodeficiency	− 0.14419	0.008395	− 6.46014	1.51E−10	7.95E−10
Citrate cycle TCA cycle	0.101751	− 0.02543	5.872817	5.52E−09	2.57E−08

AveExpr: average expression, adj. *p *val: adjust *p* value.

### Establishment of m^6^A phenotype-gene signature and functional annotations

To investigate the biological behavior of different m^6^A modification-related patterns, we identified 810 m^6^A phenotype-related DEGs using the “limma” package (Supplementary Fig. S3b). The GO and KEGG^35^ enrichment analyses for the DEGs were performed by the “clusterProfiler” package. The significantly enriched biological processes are shown in Supplementary Tables S2 and S3. The DEGs were closely related to immunity, implying that m^6^A modification played a vital role in tumor immune regulation. The immunity-related biological processes included positive regulation of PD-L1 expression, T cell activation, leukocyte activation, Th1 and Th2 cell differentiation, Th17 cell differentiation, PD-1 checkpoint pathway in cancer, etc. (Fig. 3b,c). To validate this finding, unsupervised clustering analysis based on 810 m^6^A phenotype-related genes was performed. Patients were then classified into different genomic subgroups. Consistent with the grouping by m^6^A modification-related patterns, three distinct m^6^A modification genomic phenotypes were identified using the unsupervised clustering algorithm, named m^6^A genecluster-A–C, respectively. There were 467 cases in m^6^A genecluster-A, 281 cases in m^6^A genecluster-B, and 682 cases in m^6^A genecluster-C (Supplementary Fig. S3c). Further analysis showed that patients with m^6^Acluster-B and the poorest survival were mainly assigned to m^6^A genecluster-A and -B, while those with m^6^Acluster-A/-C and survival advantages were mainly assigned to m^6^A genecluster-C (Figs. 2c, 3d; Supplementary Table S1). Patients with stage I and II LUAD were mainly characterized by m^6^A genecluster-C, which was proven to be related to a better prognosis, while those with stage III and IV LUAD were characterized by m^6^A genecluster-A and -B, with a poorer clinical outcome (Figs. 2c, 3e–f; Supplementary Table S1). These results furtherly confirmed that m^6^A modification played a crucial role in the progression and prognosis of LUAD. In these m^6^A gene clusters, differential expression of m^6^A regulators was observed. M^6^A genecluster-A was characterized by upregulated expression of WTAP and IGFBP3; m^6^A genecluster-B showed increased expression of RBM15B, YTHDF3, LRPPRC, HNRNPC, IGFBP1, IGFBP2, and ALKBH5; m^6^A genecluster-C exhibited significantly increased expression of METTL3, METTL14, RBM15, YTHDC1, YTHDC2, YTHDF2, FMR1, RBMX, and FTO (Fig. 3g).

### Clinical and transcriptome characteristics of three m^6^A modification-related patterns

**Figure 4:**
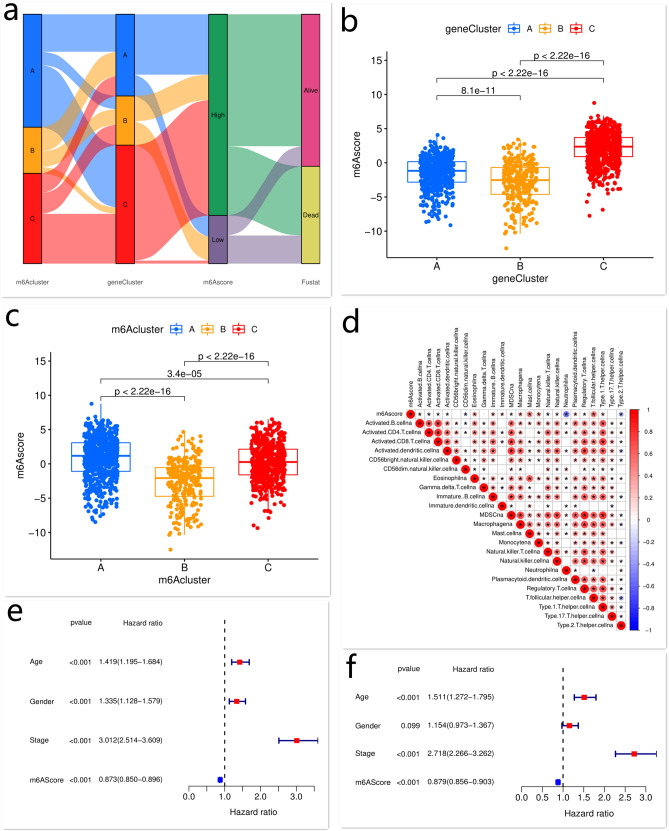
The relationships between the m^6^Ascore and molecular characteristics. The relationships among m^6^Acluster, m^6^A genecluster, survival status (Fustat), and m^6^Ascore were visualized using the alluvial diagram (**a**). The m^6^Ascore in different m^6^A genecluster and m^6^Acluster was obtained by the Kruskal–Wallis test (**b**, **c**). The correlation between TME-infiltrating immune cells and the m^6^Ascore in LUAD (**d**). Univariate and multivariate analyses of the m^6^Ascore (**e**, **f**). This figure is created using the R (version 4.0.3) (https://www.r-project.org/).

The above results suggested that m^6^A modification played a key regulatory role in shaping the TME landscape. However, these data were based on the patient population, not at the individual level. Considering the complexity and heterogeneity of m^6^A modification in different individuals, a scoring system was developed based on m^6^A phenotype-related genes to quantify the m^6^A modification-related pattern of each patient, and the results were shown as the m^6^Ascore. The alluvial diagram was used to visualize the attribute changes of each individual (Fig. 4a). The Kruskal–Wallis test showed significant differences in the m^6^Ascore among different m^6^A gene clusters. M^6^A genecluster-C was related to a better prognosis and showed a higher median score compared with m^6^A genecluster-A and -B (Fig. 4b). In addition, m^6^Acluster-A and -C showed significantly increased m^6^Ascore compared with m^6^Acluster-B (Fig. 4c). Previous analysis demonstrated that patients with m^6^Acluster-A had a survival advantage and this pattern was remarkably rich in infiltrating immune cells, indicating that high m^6^Ascore may be correlated with immune activation-related signature and survival advantage. To better elucidate the characteristics of the m^6^A signature, we examined the correlation between the m^6^Ascore and TME-infiltrating immune cells in LUAD (Fig. 4d). The results showed that high m^6^Ascore was significantly correlated with immune activation.

**Figure 5:**
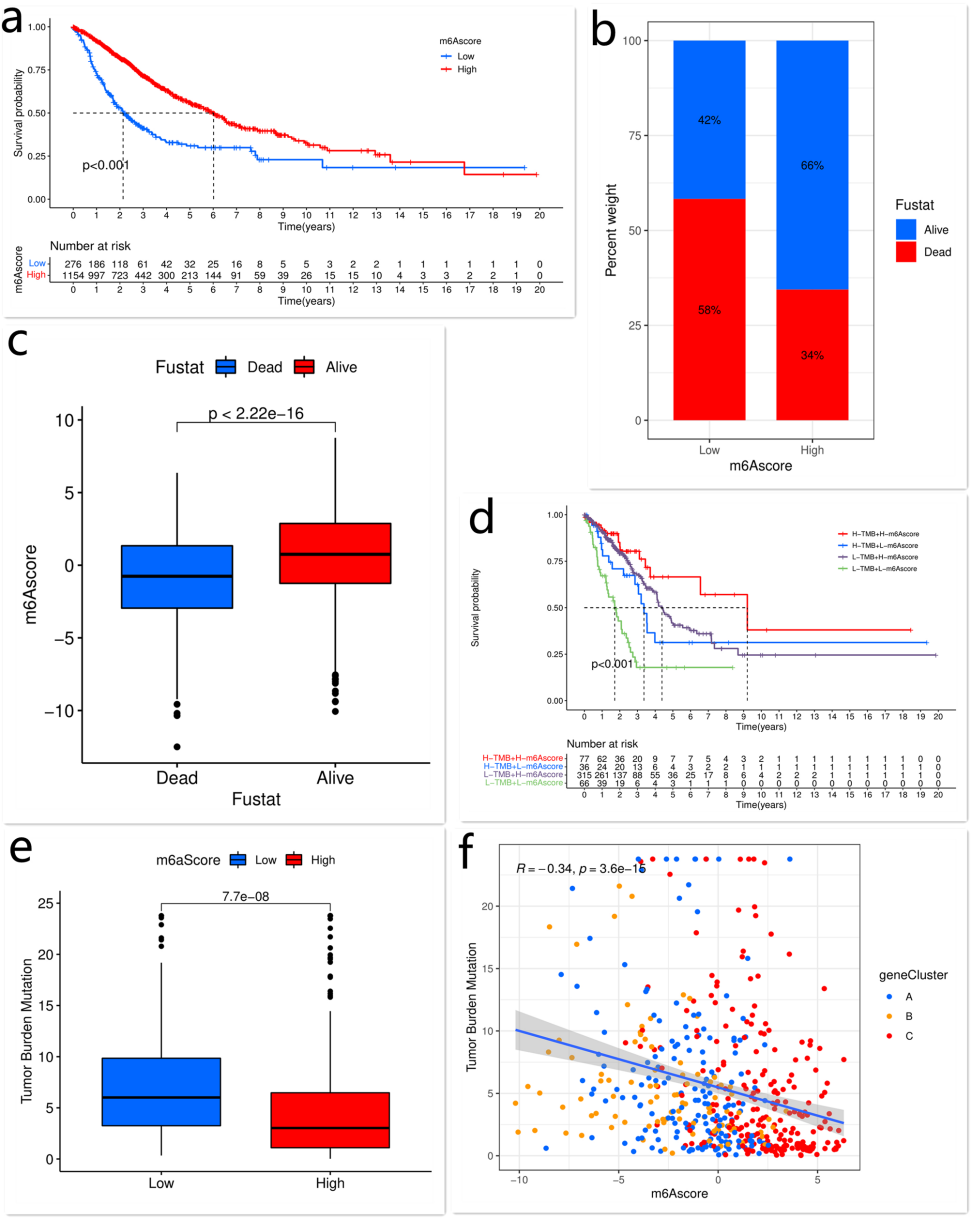
The correlations of the m^6^Ascore with clinical characteristics and TMB. The Kaplan–Meier curves of the OS of LUAD patients with low or high m^6^Ascore (**a**). The correlation between the m^6^Ascore and survival status (Fustat) of patients (**b**, **c**). The Kaplan–Meier curves of the OS of subgroup patients stratified by the m^6^Ascore and TMB (**d**). The relationship between the m^6^Ascore and TMB (**e**, **f**). This figure is created using the R (version 4.0.3) (https://www.r-project.org/).

To evaluate the prognostic value of the m^6^Ascore for patients’ outcomes, LUAD patients were classified into the low and high m^6^Ascore groups with the cut-off value determined by the “survminer” R package. Next, we investigated whether the m^6^Ascore was an independent prognostic biomarker for LUAD. The univariate and multivariate Cox regression model analyses, which included the clinical and demographic factors of patients (i.e. gender, age, and TNM stage), confirmed that the m^6^Ascore was an independent and robust prognostic marker for the outcome of LUAD patients and was inversely associated with the risk of LUAD (Fig. 4e,f). Further analysis showed that patients with a high m^6^Ascore had a significant survival benefit, which was consistent with the above results (Fig. 5a–c). To further assess the stability of the m^6^Ascore model, the prognostic value of the risk score for LUAD patients with different clinical characteristics, including age, gender, and TNM stage, was evaluated (Supplementary Fig. S4a–f). The results also showed that high m^6^Ascore was correlated with a better clinical benefit. In addition, we examined whether the combination of the m^6^Ascore and the mutation signatures of m^6^A regulators could predict the survival of patients with LUAD. We found that patients with high m^6^Ascore and mutation frequency had a better prognosis, while those with low m^6^Ascore and mutation frequency experienced poor outcomes (Fig. 5d). The other result obtained from this analysis was that patients with a high m^6^Ascore always showed a survival advantage, independent of the mutation frequency (Fig. 5d).

### Characteristics of m^6^A modification in tumor somatic mutation

**Figure 6:**
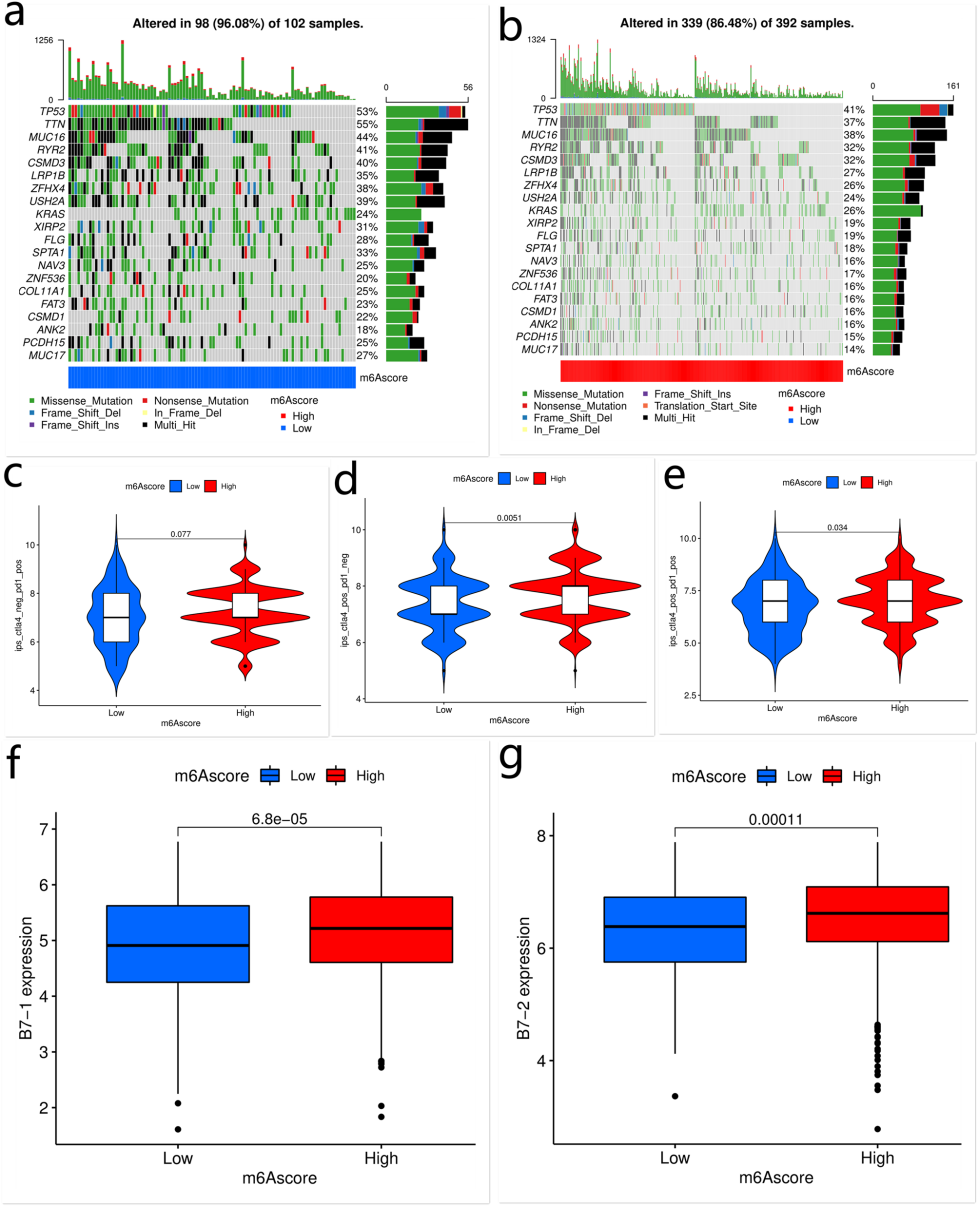
The frequency of all mutated gene and immunotherapeutic response of patients with low or high m^6^Ascore. The frequency of all mutated genes in LUAD in the low and high m^6^Ascore groups (**a**,**b**). The effectiveness of anti-CTLA4 and/or anti-PD-1 immunotherapies in the low and high m^6^Ascore groups. “ctla4-neg-pd1-pos” indicates patients treated with anti-PD-1 therapy alone; “ctla4-pos-pd1-neg” indicates patients treated with anti-CTLA4 therapy alone; “ctla4-pos-pd1-pos” indicates patients treated with both anti-CTLA4 and anti-PD-1 therapies (**c**–**e**). The expressions of B7-1 and B7-2 in the low and high m^6^Ascore groups (**f**,**g**). This figure is created using the R (version 4.0.3) (https://www.r-project.org/).

The difference in the distribution of somatic mutation between high and low m^6^Ascore groups in the TCGA-LUAD cohort was analyzed using the “maftools” package. The low m^6^Ascore group showed more extensive TMB compared with patients with a high m^6^Ascore (rate of all mutated gene: 96.08% *vs.* 86.48%) (Figs. 5e,f, 6a,b). The TMB quantification analysis showed that the m^6^Ascore and TMB were negatively corrected.

### The role of m^6^A modification-related patterns in anti-CTLA4 immunotherapy

The anti-CTLA4 and anti-PD-1 immunotherapies have emerged as promising options for cancer therapy. We examined whether the m^6^A modification-related pattern could predict the response of patients to anti-CTLA4 and anti-PD-1 therapies. Patients treated with anti-CTLA4 immunotherapy exhibited significant clinical benefits (Fig. 6c–e). In addition, compared to the low m^6^Ascore group, patients with a high m^6^Ascore showed significant therapeutic advantage and better clinical response to anti-CTLA4 therapy (Fig. 6d). Patients with a high m^6^Ascore also showed upregulated expressions of B7-1 (CD80) and B7-2 (CD86), indicating a potential response to anti-CTLA4 therapy (Fig. 6f,g). These data implied that the m^6^Ascore was a robust biomarker for predicting the clinical response and prognosis of LUAD patients. Taken together, our study showed that m^6^A modification-related patterns were significantly correlated with tumor immune phenotypes and clinical response to anti-CTLA4 therapy. The established m^6^A modification-related signature may be used to predict the response of LUAD patients to anti-CTLA4 immunotherapy.

## Discussion

As the most common RNA modification, m^6^A methylation plays an important role in post-transcriptional regulation^22,36^. Aberrant m^6^A modification is closely associated with the onset and development of cancers^22,23^. Increasing evidence has shown that m^6^A modification plays a key role in TME infiltration of immune cells and tumor immunotherapy. However, the mechanisms by which m^6^A modification affects TME infiltration and immunotherapy have not been fully elucidated. Moreover, previous studies mainly focused on a single TME cell type or regulator. The regulatory effects of multiple m^6^A regulators on the overall characteristics of TME infiltration in LUAD warrant further investigation.

In this study, we identified three distinct m^6^A modification-related patterns with significantly distinct biological characteristics by 23 m^6^A regulators. Patients with m^6^Acluster-A showed a survival advantage and the enriched pathways were associated with immune activation. Patients with m^6^Acluster-B had poor survival and the enriched pathways were associated with carcinogenic activation. The pathways enriched in patients with m^6^Acluster-C were related to substance metabolism. In addition, m^6^Acluster-A was remarkably rich in TME-infiltrating immune cells. These findings were consistent with a previous study, showing that an imbalanced immune system played a pivotal role in tumor progression^13,37^. LUAD is an immunosuppressive disorder that is implicated in TME cell infiltration^14,38^. Cancer patients with abundant TME-infiltrating immune cells showed a survival advantage^39,40^. TME is regulated by various immunoregulatory signals that are involved in the initiation, development, and metastasis of LC, and its heterogeneity may lead to multiple dimensions in the therapeutic response and prognosis of patients^11,12,41,42^. A previous study reported that immunotherapy promoted the therapeutic effects of NSCLC treatment by activating the host immune system and regulating TME^43^. In this study, by analyzing TME infiltration and survival outcome of each cluster, we validated the reliability of immune phenotype classification for distinct m^6^A modification-related patterns. These findings suggested that TME-infiltrating immune cells protected against LUAD and had an effect on LUAD immunotherapy.

Next, the DEGs in distinct m^6^A modification-related patterns were identified, referring to m^6^A phenotype-related genes. Based on these DEGs, patients with LUAD were divided into three groups. Further analysis showed that the DEGs were closely related to immunity, indicating that m^6^A modification plays a vital role in the classification of TME. The characteristics of TME-infiltrating immune cells in LUAD were further investigated by a comprehensive assessment of m^6^A modification-related patterns. Considering the heterogeneity and complexity of m^6^A modification in different individuals, a scoring system was developed to quantify the m^6^A modification-related pattern of each patient, and the results were shown as the m^6^Ascore. The m^6^A modification-related pattern that was rich in infiltrating immune cells was characterized by significantly increased m^6^Ascore and survival advantage. The univariate and multivariate Cox regression model analyses identified the m^6^Ascore as an independent prognostic marker for the outcome of LUAD patients. Additionally, the m^6^Ascore was a reliable prognostic factor for LUAD patients with different clinical characteristics, including gender, age, and TNM stage. These data suggest that the m^6^Ascore may be used to comprehensively assess individual m^6^A modification-related pattern and therefore to determine TME infiltration pattern, that is, tumor immune phenotype. Further analysis revealed that the high m^6^Ascore group had lower TMB than the low m^6^Ascore group. The missense mutation is closely related to immunotherapy^44^. The study by Samstein et al. found that patients with higher somatic TMB had better immunotherapy responses^45^. The mutation is also related to the activation of immune cells. HNSCC patients with low TMB had increased numbers of CD^4+^ memory resting cells and B memory cells, as well as a better prognosis^46^.

Although anti-CTLA4 and anti-PD-1 immunotherapies have emerged as promising approaches for treating LUAD, especially advanced LUAD, individual heterogeneity remains a critical challenge. Therefore, it is of great importance to identify novel markers that could predict the outcomes of immunotherapies. In this study, we showed that m^6^A modification significantly affected the TME landscape in LUAD, implying that the therapeutic efficacy of immunotherapy may be affected by m^6^A modification. Additionally, patients treated with anti-CTLA4 immunotherapy exhibited significant clinical benefits. Patients with a high m^6^Ascore showed significant therapeutic advantage and better clinical response to anti-CTLA4 therapy. Meanwhile, Patients with high m^6^Ascores also showed upregulated expressions of B7-1 and B7-2. Previous studies have reported that CTLA-4 was a negative regulator of T cell activation. The binding of CTLA-4 to B7-1 and B7-2 ligands inhibited T cell activation. Meanwhile, anti-CTLA4 immunotherapy augments antitumor responses by inhibiting B7-1 and B7-2 ligands of T cells^47–49^. Here, we showed that m^6^A modification significantly affected the response of LUAD patients to immunotherapy and the m^6^Ascore was a predictor of clinical response to anti-CTLA4 immunotherapy in this population.

Our study provided a new perspective of individualized immunotherapy and immuno-oncology for LUAD. However, some limitations of the current study need to be addressed. The data were obtained from TCGA and GEO databases. Due to insufficient clinical cohort, the proposed model and interactions among m^6^A modification, TME, and immunotherapy, warrant clinical verification. Future large-cohort, prospective clinical trials are needed.

## Conclusions

This study showed the regulatory mechanisms of m^6^A modification on TME in LUAD patients. The response of patients with different m^6^Ascore to immunotherapy was comprehensively assessed. Our findings may contribute to the improvement of current immunotherapy and the development of individualized immunotherapy for LUAD patients.

## Methods

### Data source and preprocessing

**Figure 7:**
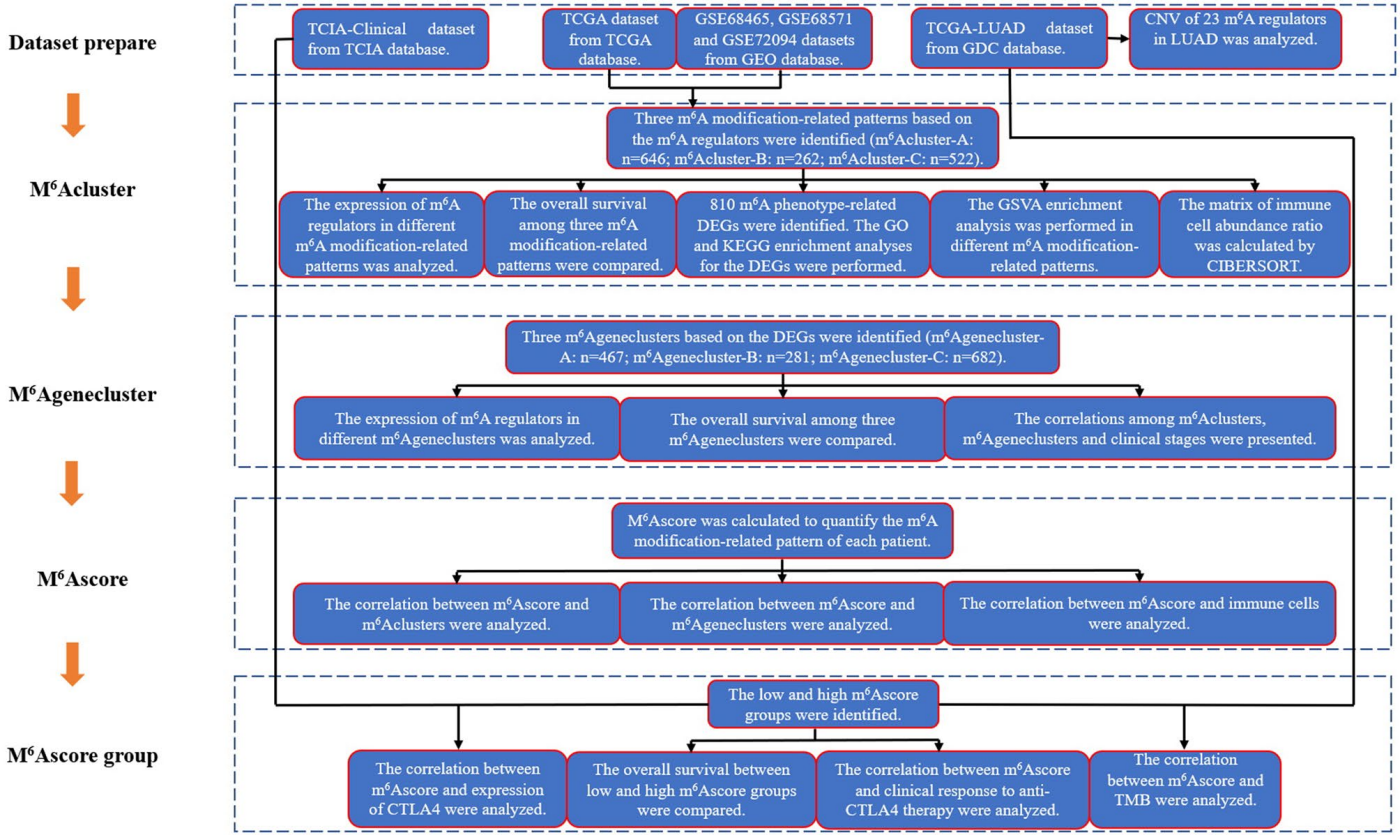
The workflow of our study.

The workflow of our study was shown in Fig. 7. The RNA sequencing transcriptome of LUAD patients and corresponding clinical data were obtained from TCGA (https://portal.gdc.cancer.gov/) and GEO (https://www.ncbi.nlm.nih.gov/geo/) databases. Patients without survival information were excluded. A total of four eligible LUAD cohorts (GSE68465, GSE68571, GSE72094, and The Cancer Genome Atlas-Lung Adenocarcinoma (TCGA-LUAD)) were gathered for further analyses. The R (version 4.0.3) (https://www.r-project.org/) and R Bioconductor packages (https://www.bioconductor.org/) were used for data analysis. For the TCGA datasets, the RNA sequencing data (FPKM value) obtained from the Genomic Data Commons (GDC) were transformed into transcripts per kilobase million (TPM) values. The somatic mutation data obtained from TCGA were used to demonstrate the mutation frequency of m^6^A regulators in LUAD using the “maftools” R package. The copy number variation (CNV) data obtained from UCSC Xena (https://xena.ucsc.edu/) database were used for Copy Number Variation analysis using the R (version 4.0.3). The baseline information of LUAD patients on the datasets of our study was shown in Table 5.

**Table 5:** The baseline information of LUAD patients on the datasets of our study.

Characteristics	TCGA		GSE68465		GSE68571		GSE72094		TCIA-ClinicalData	
	Number of cases	Percentages (%)	Number of cases	Percentages (%)	Number of cases	Percentages (%)	Number of cases	Percentages (%)	Number of cases	Percentages (%)
**Sex**										
Female	280	53.60	220	49.66	51	59.30	240	54.30	280	48.78
Male	242	46.40	223	50.34	35	40.70	202	45.70	242	42.16
Unknown	0	0.00	0	0.00	0	0.00	0	0.00	52	9.06
**Age**										
≤ 65	241	46.17	231	52.14	50	58.14	127	28.73	236	41.11
> 65	262	50.19	212	47.86	36	41.86	294	66.52	255	44.43
Unknown	19	3.64	0	0.00	0	0.00	21	4.75	83	14.46
**Race**										
White	–	–	295	66.59	–	–	399	90.27	393	68.47
Black	–	–	12	2.71	–	–	13	2.94	53	9.23
Unknown	–	–	136	30.70	–	–	30	6.79	128	22.30
**Smoking**										
Yes	–	–	300	67.72	74	86.05	335	75.79	356	62.02
No	–	–	49	11.06	9	10.46	33	7.47	0	0.00
Unknown	–	–	94	21.22	3	3.49	74	16.74	218	37.98
**Survival time**										
≤ 5 years	460	88.12	273	61.62	65	75.58	393	88.91	460	80.14
> 5 years	53	10.15	169	38.15	21	24.42	5	1.13	53	9.23
Unknown	9	1.73	1	0.23	0	0.00	44	9.96	61	10.63
**Survival status**										
Alive	334	64.98	207	46.73	62	72.09	298	67.42	334	58.19
Dead	188	36.02	236	53.27	24	27.91	122	27.60	197	34.32
Unknown	0	0.00	0	0.00	0	0.00	22	4.98	43	7.49
**Stage**										
Stage I	279	53.45	276	62.30	67	77.91	265	59.95	279	48.61
Stage II	124	23.76	95	21.44	0	0.00	69	15.61	124	21.60
Stage III	85	16.28	69	15.58	19	22.09	63	14.25	85	14.81
Stage IV	26	4.98	0	0.00	0	0.00	17	3.85	26	4.53
Unknown	8	1.53	3	0.68	0	0.00	28	6.34	60	10.45

### Unsupervised clustering for 23 m^6^A regulators

A total of 23 m^6^A regulators were collected from previous studies (Table 1). To determine their biological and functional characteristics in LUAD, unsupervised clustering algorithm was used to categorize LUAD patients according to their m^6^A modification-related patterns using the “ConsensusClusterPlus” package. This algorithm was applied 1000 times to ensure the stability of classification.

### Gene set variation analysis (GSVA) and functional annotations

To investigate the biological processes and pathways in different m^6^A modification-related patterns, the “GSVA” R packages and “c2.cp.kegg.v6.2.symbols” gene sets were obtained for GSVA. A *p*-value of less than 0.05 indicated significantly enriched biological processes and pathways. The functional annotations of m^6^A-related genes were analyzed using the “clusterProfiler” R package. The cut-off value was set as a *p* < 0.05.

### Estimation of TME infiltration

The relative abundance of TME-infiltrating cells in individual samples was yielded using the single-sample gene set enrichment analysis (ssGSEA). The enrichment score was obtained from the ssGSEA and differential immune cell infiltration among different subsets was analyzed.

### Identification of differentially expressed genes (DEGs) among different m^6^A phenotypes

Patients were divided into three groups according to their m^6^A modification-related patterns. A *p*-value of < 0.05 was used to identify DEGs using the “Limma” R package.

### Establishment of m^6^A phenotype-related gene signature

A scoring system was developed to quantify the m^6^A modification-related pattern of each patient and the m^6^A phenotype-related gene signature was termed the m^6^Ascore. The gene signature was established as follows: Unsupervised clustering algorithm was used to identify overlapped DEGs and then to divide patients into different subsets. To define the number of clusters and their stability, consensus clustering algorithm was applied. Then, a univariate Cox regression model was established to determine the prognostic value of each gene. The genes with significant prognostic value were extracted for further analyses. Subsequently, principal component analysis (PCA) was performed to establish the m^6^A phenotype-related gene signature. The m^6^Ascore was calculated using the following equation^50,51^:

$${{m}}^{6} {{Ascore}} = \sum \left( {{{PC}}1i + {{PC}}2i} \right)$$

$i$ indicates the expression of m^6^A phenotype-related genes.

### Data of immune-checkpoint blockade

To evaluate the therapeutic response of patients with distinct m^6^A modification-related patterns to CTLA4 and PD-1 blockade therapies, the TCIA-Clinical Data of LUAD was downloaded from The Cancer Immunome Atlas (TCIA) database (https://tcia.at/). Four groups of patients were included in our study: (1) patients treated with both anti-PD-1 and anti-CTLA4 immunotherapies; (2) patients treated with anti-CTLA4 therapy but not anti-PD-1 therapy; (3) patients treated with anti-PD-1 therapy but not anti-CTLA4 therapy; (4) patients not treated with anti-PD-1 or anti-CTLA4 therapy. The immunotherapy score of each patient was obtained for further analysis. Then, the correlation between immunotherapy effectiveness and the m^6^Ascore was examined. The expression levels of B7-1 and B7-2 were also obtained from the above databases.

### Statistical analysis

Spearman and distance correlation analyses were performed to assess the correlation between the expression of m^6^A regulators and TME-infiltrating immune cells. Kruskal–Wallis tests and one-way ANOVA were used to compare the results among three or more subgroups. The “survminer” R package was used to calculate the cut-off point of each dataset subgroup according to the correlation between the m^6^Ascore and patients’ survival. The “surv-cutpoint” function, which repeatedly tested all potential cut points to find the one achieving the maximum rank statistic, was used to dichotomize the m^6^Ascore. Subsequently, patients were classified into the low and high m^6^Ascore groups using the maximally selected log-rank statistics to minimize the batch effect. The Kaplan–Meier method was applied to visualize the survival curves and log-rank tests were used to identify statistical significance. The univariate and multivariate Cox regression model analyses were used to identify independent prognostic factors. The forest plots of prognostic factors were generated using the “forestplot” R package. The waterfall function of the “maftools” R package was used to demonstrate the mutation landscape of LUAD patients with low or high m^6^Ascore. The CNV landscape of 23 m^6^A regulators in 23 pairs of chromosomes was delineated using the “RCircos” R package. A *p*-value of less than 0.05 indicated statistical significance. The R (version 4.0.3) was used for data analysis.

### Ethical approval

My study did not require ethical approval.

## Data availability

The following information was supplied regarding data availability: The datasets are available at the TCGA (https://portal.gdc.cancer.gov/), GEO (https://www.ncbi.nlm.nih.gov/geo/), and TCIA (https://tcia.at/) databases.

## Supplementary Information

Supplementary Information.
