# Supplementary material for: Multi-omics analysis of m6A modification-related patterns based on m6A regulators and tumor microenvironment infiltration in lung adenocarcinoma
Source: Sci Rep. 2021 Oct 22;11:20921. doi: 10.1038/s41598-021-00272-z (PMC8536683; doi:10.1038/s41598-021-00272-z)
Supplement: Supplementary file 1 — Supplementary Information. [file 41598_2021_272_MOESM1_ESM.docx]

Multi-omics analysis of m^6^A modification-related patterns based on m^6^A regulators and tumor microenvironment infiltration in lung adenocarcinoma

**Authors:**

Xincheng Wu^1^, Ph.D. student

Zhengping Bai^2*^, Prof **(corresponding author)**

**^1^ Affiliation:** Hunan University of Chinese Medicine, Changsha, China.

**Address:** 300 Xueshi Rd., Yuelu District, 410208, Changsha, China

**Telephone number:** +86-0731-88458277

**E-mail:** 465006379@qq.com

**ORCID:** 0000-0002-9523-4568

**^2^ Affiliation:** Hunan Academy of Chinese Medicine, Changsha, China.

**Address:** 58 Lushan Rd., Yuelu District, 410006, Changsha, China

**Telephone number:** +86-0731-88883684

**E-mail:** baizhengping@yeah.net

**ORCID:** 0000-0002-1296-1138

^*^ Zhengping Bai is corresponding author on this work.

The authors declare that there are no conflicts of interest in this work.

**Supplementary Figure S1 The relations of m^6^A regulators in LUAD (The larger version of Figure 2e).**


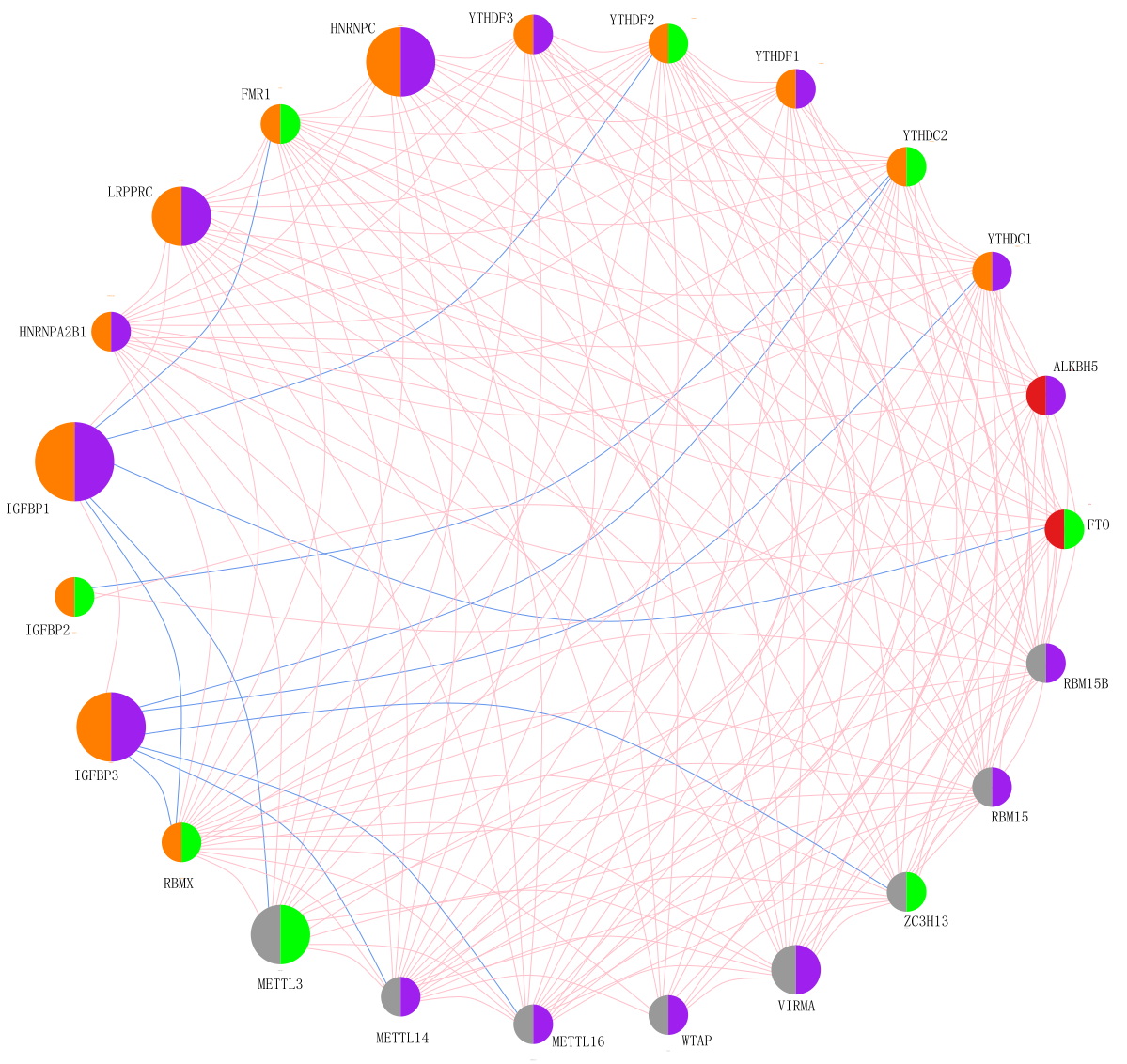


The circle size indicates the survival impact of each m^6^A regulator. The lines connecting m^6^A regulators indicate their relations. The thickness of line indicates the strength of the relation. The red lines indicate positive relations, while the blue lines indicate negative relations. This figure is created using the R (version 4.0.3) (<https://www.r-project.org/>).

**Supplementary Figure S2 The expression of mutant and wild-type m^6^A regulators.**


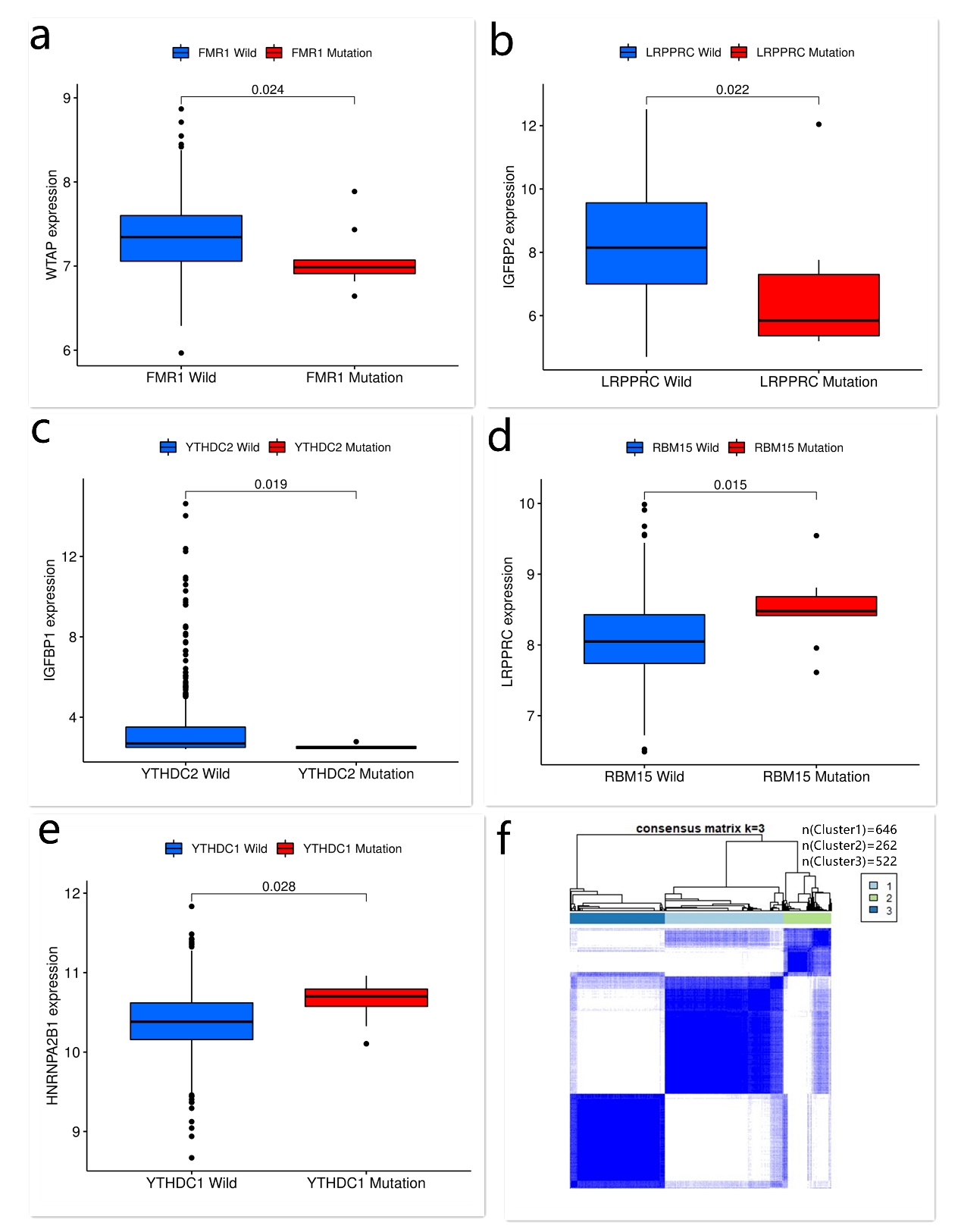


The expression of mutant and wild-type m^6^A regulators was compared (**a**, **b**, **c**, **d,** and **e**). Consensus clustering matrix for k=3 based on m^6^A regulators (**f**). This figure is created using the R (version 4.0.3) (<https://www.r-project.org/>).

**Supplementary Figure S3 Gene characteristics in different m^6^A modification patterns.**


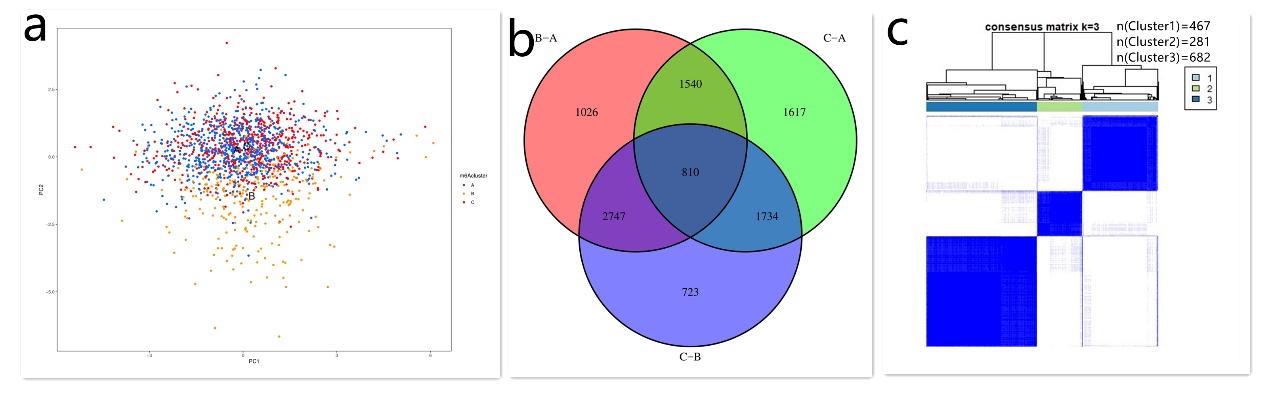


Principal component analysis of gene expression in different m^6^A modification patterns (**a**). The DEGs among distinct m^6^A modification patterns (**b**). Consensus clustering matrix for k=3 based on DEGs (**c**). This figure is created using the R (version 4.0.3) (<https://www.r-project.org/>).

**Supplementary Figure S4 The Kaplan-Meier curves of the OS of LUAD patients with different clinical characteristics.**


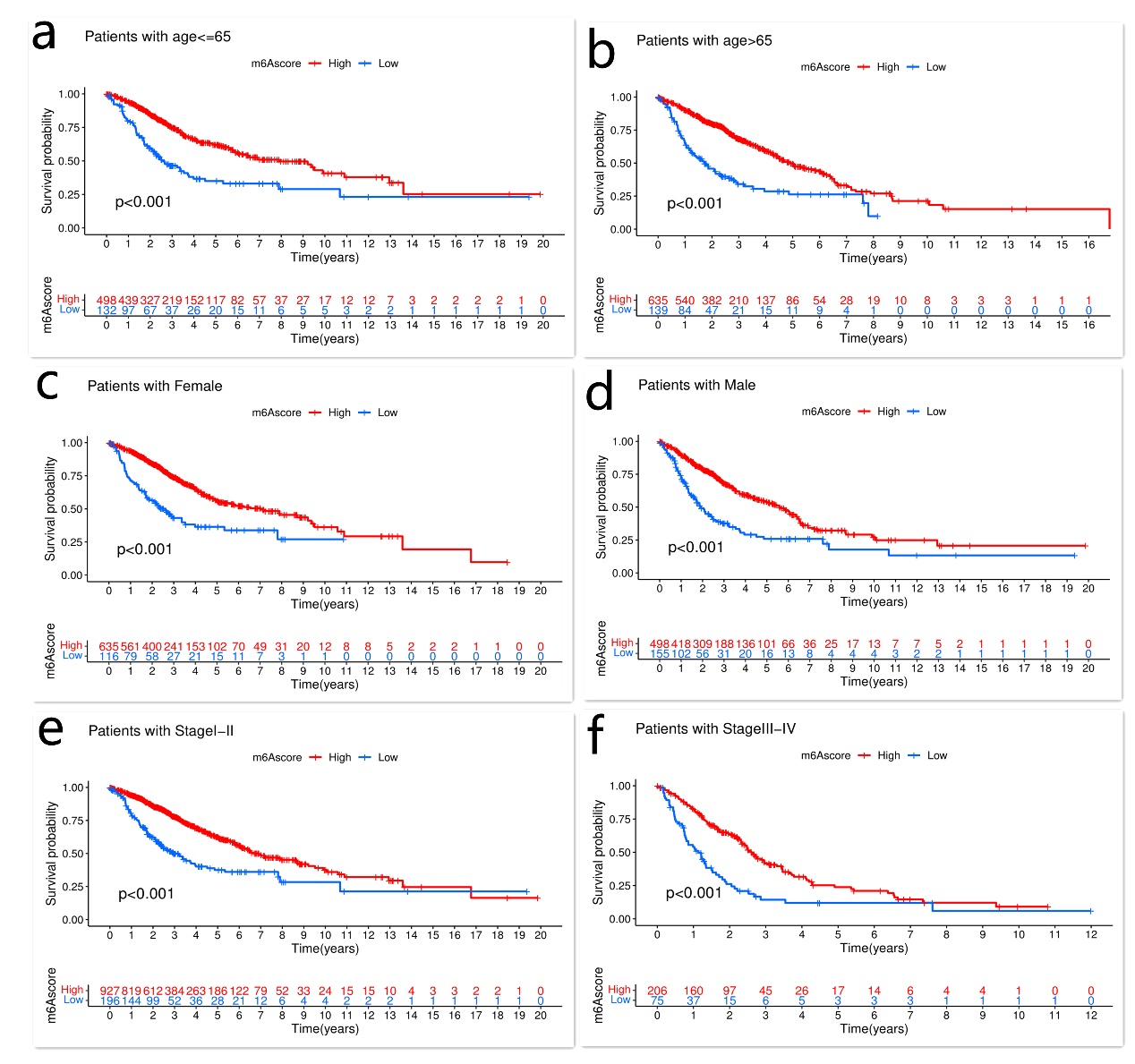


Age≦65 (**a**), age>65 (**b**), female (**c**), male (**d**), early TNM stage (**e**), and late TNM stage (**f**). This figure is created using the R (version 4.0.3) (<https://www.r-project.org/>).

**Supplementary Table S1 The baseline information,** **m^6^A modification-related pattern, and m^6^A modification genomic phenotype of each LUAD patient on the datasets of our study**

| id | Age | Gender | Stage | Survival status | m^6^Acluster | | geneCluster |
| --- | --- | --- | --- | --- | --- | --- | --- |
| GSM1672281 | 74 | Female | Stage IA | Alive | | C | C |
| GSM1672282 | 74 | Female | Stage IA | Alive | | C | C |
| GSM1672283 | 74 | Female | Stage IB | Dead | | A | C |
| GSM1672284 | 82 | Female | Stage IA | Alive | | B | C |
| GSM1672285 | 59 | Female | Stage IB | Dead | | B | A |
| GSM1672286 | 60 | Female | Stage IA | Alive | | C | C |
| GSM1672287 | 54 | Female | Stage IA | Alive | | A | C |
| GSM1672288 | 63 | Female | Stage IA | Alive | | A | C |
| GSM1672289 | 63 | Female | Stage IA | Dead | | A | C |
| GSM1672290 | 66 | Male | Stage IIB | Dead | | A | C |
| GSM1672291 | 52 | Female | Stage IA | Alive | | A | C |
| GSM1672292 | 62 | Female | Stage IA | Dead | | A | C |
| GSM1672293 | 60 | Male | Stage IA | Dead | | B | B |
| GSM1672294 | 66 | Female | Stage IB | Dead | | A | C |
| GSM1672295 | 61 | Female | Stage IB | Alive | | C | C |
| GSM1672296 | 68 | Female | Stage IB | Dead | | C | B |
| GSM1672297 | 48 | Female | Stage IB | Dead | | C | B |
| GSM1672298 | 75 | Female | Stage IA | Dead | | B | C |
| GSM1672299 | 50 | Female | Stage IB | Alive | | A | B |
| GSM1672300 | 75 | Female | Stage IA | Dead | | C | C |
| GSM1672301 | 72 | Female | Stage IB | Dead | | A | C |
| GSM1672302 | 72 | Female | Stage IIIA | Dead | | B | B |
| GSM1672303 | 71 | Male | Stage IIIB | Dead | | B | B |
| GSM1672304 | 58 | Male | Stage IIIA | Dead | | A | A |
| GSM1672305 | 63 | Male | Stage IIIA | Dead | | C | B |
| GSM1672306 | 74 | Female | Stage IIIA | Dead | | A | A |
| GSM1672307 | 76 | Male | Stage IB | Dead | | A | C |
| GSM1672308 | 59 | Male | Stage IA | Dead | | B | A |
| GSM1672309 | 71 | Male | Stage IIIA | Dead | | B | B |
| GSM1672310 | 45 | Female | Stage IIB | Dead | | A | A |
| GSM1672311 | 78 | Female | Stage IIIA | Dead | | A | C |
| GSM1672312 | 75 | Male | Stage IB | Alive | | A | A |
| GSM1672313 | 61 | Male | Stage IB | Dead | | A | C |
| GSM1672314 | 65 | Male | Stage IA | Alive | | C | A |
| GSM1672315 | 69 | Male | Stage IA | Alive | | B | B |
| GSM1672316 | 73 | Male | Stage IB | Dead | | A | A |
| GSM1672317 | 51 | Male | Stage IB | Dead | | B | A |
| GSM1672318 | 63 | Female | Stage IB | Dead | | C | B |
| GSM1672319 | 60 | Male | Stage IA | Alive | | A | C |
| GSM1672320 | 42 | Male | Stage IA | Alive | | B | A |
| GSM1672321 | 56 | Male | Stage IIB | Dead | | A | A |
| GSM1672322 | 69 | Female | Stage IA | Alive | | C | C |
| GSM1672323 | 58 | Female | Stage IIIA | Dead | | A | A |
| GSM1672324 | 62 | Male | Stage IIIA | Dead | | A | A |
| GSM1672325 | 62 | Female | Stage IA | Alive | | A | A |
| GSM1672326 | 65 | Male | Stage IB | Alive | | A | C |
| GSM1672327 | 73 | Female | Stage IB | Alive | | A | A |
| GSM1672328 | 52 | Female | Stage II | Dead | | C | A |
| GSM1672329 | 72 | Male | Stage IA | Dead | | B | A |
| GSM1672330 | 65 | Male | Stage IA | Dead | | A | C |
| GSM1672331 | 65 | Female | Stage IA | Alive | | B | B |
| GSM1672332 | 59 | Female | Stage IB | Alive | | A | B |
| GSM1672333 | 62 | Female | Stage IA | Alive | | A | C |
| GSM1672334 | 63 | Male | Stage IB | Alive | | C | A |
| GSM1672335 | 54 | Female | Stage IA | Dead | | A | C |
| GSM1672336 | 57 | Female | Stage IA | Alive | | A | A |
| GSM1672337 | 59 | Female | Stage IA | Alive | | C | B |
| GSM1672338 | 48 | Female | Stage IA | Alive | | B | A |
| GSM1672339 | 44 | Female | Stage IA | Alive | | B | B |
| GSM1672340 | 40 | Female | Stage IA | Alive | | C | A |
| GSM1672341 | 64 | Male | Stage IIIA | Dead | | B | A |
| GSM1672342 | 69 | Male | Stage IIIA | Dead | | B | A |
| GSM1672343 | 67 | Male | Stage IA | Dead | | A | A |
| GSM1672344 | 62 | Female | Stage IA | Alive | | A | C |
| GSM1672345 | 60 | Male | Stage IB | Alive | | C | B |
| GSM1672346 | 46 | Male | Stage IA | Alive | | C | B |
| GSM1672347 | 77 | Male | Stage IIIA | Dead | | C | C |
| GSM1672348 | 56 | Male | Stage IIIA | Dead | | C | A |
| GSM1672349 | 53 | Female | Stage IA | Alive | | C | C |
| GSM1672350 | 51 | Male | Stage IB | Alive | | A | A |
| GSM1672351 | 63 | Female | Stage II | Alive | | A | A |
| GSM1672352 | 85 | Female | Stage IA | Dead | | C | C |
| GSM1672353 | 60 | Female | Stage IIB | Dead | | A | A |
| GSM1672354 | 58 | Male | Stage II | Dead | | C | C |
| GSM1672355 | 81 | Male | Stage IA | Alive | | C | A |
| GSM1672356 | 52 | Female | Stage II | Dead | | C | C |
| GSM1672357 | 81 | Female | Stage II | Alive | | C | C |
| GSM1672358 | 57 | Female | Stage IA | Alive | | C | C |
| GSM1672359 | 76 | Female | Stage IA | Dead | | C | C |
| GSM1672360 | 60 | Female | Stage IIB | Dead | | A | C |
| GSM1672361 | 62 | Female | Stage IA | Alive | | B | C |
| GSM1672362 | 68 | Male | Stage II | Dead | | B | B |
| GSM1672363 | 73 | Male | Stage IA | Alive | | B | A |
| GSM1672364 | 65 | Female | Stage IIB | Alive | | C | C |
| GSM1672365 | 63 | Male | Stage IB | Alive | | C | C |
| GSM1672366 | 70 | Male | Stage IA | Alive | | B | C |
| GSM1672367 | 78 | Male | Stage IA | Alive | | A | A |
| GSM1672368 | 54 | Male | Stage II | Dead | | B | B |
| GSM1672369 | 78 | Male | Stage IIB | Dead | | C | A |
| GSM1672370 | 66 | Male | Stage IB | Alive | | C | C |
| GSM1672371 | 72 | Male | Stage IA | Alive | | A | C |
| GSM1672372 | 81 | Female | Stage IA | Dead | | A | C |
| GSM1672373 | 69 | Female | Stage IB | Alive | | C | C |
| GSM1672374 | 59 | Male | Stage IIIB | Alive | | A | A |
| GSM1672375 | 76 | Female | Stage IB | Alive | | A | C |
| GSM1672376 | 70 | Male | Stage IB | Dead | | B | B |
| GSM1672377 | 58 | Male | Stage IB | Alive | | C | C |
| GSM1672378 | 48 | Female | Stage IB | Dead | | A | C |
| GSM1672379 | 82 | Male | Stage IIB | Dead | | A | C |
| GSM1672380 | 62 | Male | Stage IA | Dead | | B | B |
| GSM1672381 | 82 | Male | Stage IIIA | Dead | | C | B |
| GSM1672382 | 68 | Male | Stage IA | Alive | | A | C |
| GSM1672383 | 58 | Male | Stage IB | Alive | | B | A |
| GSM1672384 | 63 | Male | Stage IA | Dead | | A | C |
| GSM1672385 | 78 | Male | Stage II | Dead | | A | A |
| GSM1672386 | 57 | Female | Stage IB | Dead | | C | A |
| GSM1672387 | 77 | Female | Stage IA | Alive | | C | C |
| GSM1672388 | 86 | Female | Stage IIIA | Dead | | A | C |
| GSM1672389 | 78 | Female | Stage IIIA | Alive | | A | A |
| GSM1672390 | 73 | Male | Stage IB | Alive | | A | C |
| GSM1672391 | 70 | Female | Stage IA | Alive | | B | C |
| GSM1672392 | 75 | Female | Stage IA | Alive | | A | C |
| GSM1672393 | 49 | Female | Stage IA | Alive | | B | C |
| GSM1672394 | 51 | Female | Stage IA | Alive | | C | C |
| GSM1672395 | 66 | Male | Stage IA | Alive | | C | A |
| GSM1672396 | 76 | Female | Stage IB | Alive | | C | C |
| GSM1672397 | 78 | Female | Stage IA | Alive | | B | B |
| GSM1672398 | 76 | Male | Stage IA | Dead | | A | C |
| GSM1672399 | 60 | Female | Stage IIIA | Dead | | C | A |
| GSM1672400 | 73 | Male | Stage IA | Dead | | A | C |
| GSM1672401 | 72 | Female | Stage IA | Alive | | B | A |
| GSM1672402 | 55 | Female | Stage IIIA | Dead | | A | A |
| GSM1672403 | 64 | Female | Stage IB | Alive | | A | C |
| GSM1672404 | 53 | Female | Stage IB | Alive | | B | A |
| GSM1672405 | 77 | Female | Stage IIIA | Alive | | A | C |
| GSM1672406 | 55 | Female | Stage IA | Alive | | A | C |
| GSM1672407 | 67 | Male | Stage IB | Dead | | B | A |
| GSM1672408 | 70 | Female | Stage IA | Alive | | C | A |
| GSM1672409 | 72 | Female | Stage IB | Dead | | C | A |
| GSM1672410 | 43 | Male | Stage IA | Dead | | C | A |
| GSM1672411 | 52 | Female | Stage IIB | Dead | | B | B |
| GSM1672412 | 69 | Female | Stage IA | Alive | | C | A |
| GSM1672413 | 33 | Female | Stage IB | Alive | | B | A |
| GSM1672414 | 59 | Male | Stage IIIA | Dead | | B | A |
| GSM1672415 | 62 | Male | Stage IA | Dead | | A | C |
| GSM1672416 | 70 | Male | Stage IA | Dead | | A | A |
| GSM1672417 | 74 | Male | Stage IIIA | Dead | | A | A |
| GSM1672418 | 46 | Male | Stage IB | Dead | | B | A |
| GSM1672419 | 67 | Male | Stage IIIA | Dead | | A | C |
| GSM1672420 | 60 | Male | Stage IB | Dead | | C | C |
| GSM1672421 | 75 | Male | Stage IB | Dead | | B | B |
| GSM1672422 | 64 | Male | Stage IIIA | Dead | | A | A |
| GSM1672423 | 62 | Male | Stage IB | Alive | | A | B |
| GSM1672424 | 70 | Female | Stage IA | Alive | | A | A |
| GSM1672425 | 67 | Male | Stage IIIB | Dead | | A | C |
| GSM1672426 | 56 | Female | Stage II | Dead | | A | B |
| GSM1672427 | 57 | Male | Stage IIB | Alive | | C | C |
| GSM1672428 | 81 | Female | Stage IB | Alive | | C | C |
| GSM1672429 | 65 | Female | Stage IA | Alive | | A | C |
| GSM1672430 | 73 | Female | Stage IIIA | Alive | | A | C |
| GSM1672431 | 65 | Male | Stage IA | Alive | | B | B |
| GSM1672432 | 62 | Male | Stage IA | Alive | | C | C |
| GSM1672433 | 71 | Male | Stage IIIA | Alive | | A | C |
| GSM1672434 | 82 | Female | Stage IA | Alive | | A | A |
| GSM1672435 | 68 | Male | Stage IB | Dead | | A | C |
| GSM1672436 | 48 | Female | Stage IIIA | Dead | | C | C |
| GSM1672437 | 82 | Female | Stage IB | Dead | | C | B |
| GSM1672438 | 64 | Male | Stage IIIA | Dead | | C | B |
| GSM1672439 | 68 | Male | Stage IB | Alive | | B | A |
| GSM1672440 | 58 | Male | Stage IIIA | Dead | | B | B |
| GSM1672441 | 73 | Male | Stage IA | Alive | | C | C |
| GSM1672442 | 52 | Female | Stage IIIA | Dead | | B | B |
| GSM1672443 | 62 | Male | Stage IB | Alive | | C | C |
| GSM1672444 | 51 | Male | Stage IB | Alive | | A | B |
| GSM1672445 | 62 | Male | Stage IB | Alive | | C | C |
| GSM1672446 | 50 | Female | Stage IA | Alive | | A | B |
| GSM1672447 | 65 | Male | Stage IB | Alive | | A | C |
| GSM1672448 | 74 | Male | Stage IB | Dead | | A | C |
| GSM1672449 | 55 | Female | Stage II | Alive | | A | B |
| GSM1672450 | 54 | Male | Stage IIIB | Dead | | A | C |
| GSM1672451 | 76 | Male | Stage IA | Alive | | A | B |
| GSM1672452 | 64 | Female | Stage IIIA | Dead | | A | C |
| GSM1672453 | 58 | Female | Stage IIIA | Dead | | B | A |
| GSM1672454 | 70 | Male | Stage II | Dead | | A | A |
| GSM1672455 | 57 | Female | Stage IIIA | Dead | | C | C |
| GSM1672456 | 67 | Female | Stage II | Alive | | B | B |
| GSM1672457 | 74 | Female | Stage IB | Dead | | A | B |
| GSM1672458 | 59 | Female | Stage II | Alive | | C | C |
| GSM1672459 | 66 | Female | Stage IB | Alive | | C | B |
| GSM1672460 | 67 | Male | Stage IIB | Dead | | C | C |
| GSM1672461 | 57 | Female | Stage IA | Alive | | C | C |
| GSM1672462 | 64 | Male | Stage IB | Alive | | C | B |
| GSM1672463 | 59 | Female | Stage IB | Dead | | C | A |
| GSM1672464 | 76 | Female | Stage IB | Alive | | C | C |
| GSM1672465 | 82 | Female | Stage IB | Alive | | A | C |
| GSM1672466 | 58 | Male | Stage IA | Alive | | C | C |
| GSM1672467 | 65 | Male | Stage IB | Alive | | C | C |
| GSM1672468 | 49 | Female | Stage IIIA | Alive | | C | C |
| GSM1672469 | 71 | Female | Stage IIIA | Alive | | A | C |
| GSM1672470 | 61 | Female | Stage IA | Dead | | C | C |
| GSM1672471 | 75 | Male | Stage IIIA | Dead | | A | B |
| GSM1672472 | 46 | Female | Stage IA | Alive | | C | B |
| GSM1672473 | 74 | Female | Stage IB | Dead | | A | A |
| GSM1672474 | 51 | Female | Stage II | Alive | | C | C |
| GSM1672475 | 54 | Male | Stage IA | Alive | | C | C |
| GSM1672476 | 50 | Female | Stage IIIA | Dead | | A | C |
| GSM1672477 | 69 | Female | Stage IB | Alive | | C | C |
| GSM1672478 | 63 | Female | Stage II | Alive | | C | B |
| GSM1672479 | 76 | Female | Stage IB | Dead | | A | A |
| GSM1672480 | 72 | Female | Stage II | Alive | | A | C |
| GSM1672481 | 70 | Female | Stage IA | Alive | | A | C |
| GSM1672482 | 76 | Male | Stage IA | Alive | | A | C |
| GSM1672483 | 81 | Female | Stage IA | Alive | | A | A |
| GSM1672484 | 61 | Female | Stage IB | Dead | | A | C |
| GSM1672485 | 53 | Female | Stage IA | Alive | | A | C |
| GSM1672486 | 72 | Female | Stage II | Dead | | C | B |
| GSM1672487 | 53 | Female | Stage II | Dead | | A | A |
| GSM1672488 | 61 | Male | Stage IB | Alive | | C | C |
| GSM1672489 | 66 | Male | Stage IIIA | Dead | | C | B |
| GSM1672490 | 74 | Female | Stage II | Alive | | C | B |
| GSM1672491 | 48 | Female | Stage IIIA | Dead | | A | C |
| GSM1672492 | 65 | Female | Stage IB | Alive | | C | A |
| GSM1672493 | 70 | Female | Stage IB | Dead | | C | B |
| GSM1672494 | 79 | Female | Stage IB | Dead | | A | B |
| GSM1672495 | 67 | Male | Stage IB | Dead | | A | C |
| GSM1672496 | 77 | Male | Stage IB | Alive | | A | C |
| GSM1672497 | 63 | Female | Stage IIIA | Dead | | C | C |
| GSM1672498 | 76 | Female | Stage IB | Alive | | A | C |
| GSM1672499 | 72 | Male | Stage II | Dead | | A | B |
| GSM1672500 | 51 | Female | Stage IA | Dead | | A | A |
| GSM1672501 | 63 | Female | Stage IA | Alive | | C | C |
| GSM1672502 | 80 | Female | Stage IA | Alive | | C | B |
| GSM1672503 | 74 | Female | Stage II | Alive | | A | C |
| GSM1672504 | 58 | Female | Stage IA | Alive | | A | C |
| GSM1672505 | 62 | Female | Stage IB | Alive | | A | C |
| GSM1672506 | 67 | Female | Stage IA | Alive | | C | B |
| GSM1672507 | 71 | Female | Stage IA | Alive | | C | C |
| GSM1672508 | 61 | Female | Stage IA | Alive | | C | C |
| GSM1672509 | 38 | Female | Stage IIIA | Alive | | A | C |
| GSM1672510 | 72 | Female | Stage II | Alive | | A | A |
| GSM1672511 | 43 | Female | Stage IA | Alive | | B | C |
| GSM1672512 | 65 | Female | Stage IIIA | Dead | | C | C |
| GSM1672513 | 54 | Female | Stage IB | Alive | | A | A |
| GSM1672514 | 79 | Female | Stage IIB | Dead | | C | C |
| GSM1672515 | 61 | Male | Stage IB | Dead | | C | A |
| GSM1672516 | 74 | Male | Stage IB | Alive | | A | C |
| GSM1672517 | 80 | Female | Stage IIB | Alive | | C | C |
| GSM1672518 | 68 | Female | Stage IB | Dead | | A | A |
| GSM1672519 | 64 | Female | Stage IA | Dead | | A | C |
| GSM1672520 | 58 | Female | Stage II | Alive | | A | C |
| GSM1672521 | 57 | Male | Stage IB | Alive | | C | B |
| GSM1672522 | 57 | Female | Stage IB | Alive | | A | C |
| GSM1672523 | 67 | Male | Stage IB | Alive | | A | C |
| GSM1672524 | 75 | Female | Stage II | Alive | | C | B |
| GSM1672525 | 74 | Male | Stage IA | Alive | | A | C |
| GSM1672526 | 59 | Male | Stage IB | Alive | | B | C |
| GSM1672527 | 75 | Male | Stage IB | Dead | | C | C |
| GSM1672528 | 60 | Female | Stage IB | Alive | | C | B |
| GSM1672529 | 41 | Female | Stage IB | Alive | | A | A |
| GSM1672530 | 59 | Male | Stage IA | Dead | | B | B |
| GSM1672531 | 60 | Female | Stage IB | Alive | | B | A |
| GSM1672532 | 43 | Male | Stage IIB | Alive | | C | C |
| GSM1672533 | 54 | Male | Stage IB | Dead | | A | A |
| GSM1672534 | 52 | Female | Stage II | Dead | | A | A |
| GSM1672535 | 47 | Male | Stage IB | Alive | | B | C |
| GSM1672536 | 55 | Female | Stage IB | Alive | | B | B |
| GSM1672537 | 66 | Female | Stage IB | Dead | | B | A |
| GSM1672538 | 70 | Female | Stage IIB | Dead | | C | B |
| GSM1672539 | 68 | Male | Stage IB | Dead | | C | B |
| GSM1672540 | 61 | Female | Stage IB | Dead | | C | A |
| GSM1672541 | 60 | Female | Stage IB | Alive | | B | B |
| GSM1672542 | 35 | Male | Stage IB | Alive | | C | A |
| GSM1672543 | 53 | Female | Stage II | Alive | | C | A |
| GSM1672544 | 54 | Male | Stage II | Dead | | B | A |
| GSM1672545 | 73 | Female | Stage IB | Alive | | A | C |
| GSM1672546 | 38 | Female | Stage IB | Alive | | B | A |
| GSM1672547 | 58 | Male | Stage IB | Alive | | C | A |
| GSM1672548 | 65 | Male | Stage IB | Dead | | A | C |
| GSM1672549 | 66 | Female | Stage IB | Alive | | B | A |
| GSM1672550 | 66 | Male | Stage II | Dead | | C | C |
| GSM1672551 | 76 | Male | Stage II | Dead | | B | A |
| GSM1672552 | 53 | Male | Stage IB | Dead | | A | C |
| GSM1672553 | 60 | Male | Stage IB | Alive | | A | C |
| GSM1672554 | 40 | Female | Stage IB | Alive | | B | A |
| GSM1672555 | 67 | Male | Stage II | Dead | | B | B |
| GSM1672556 | 62 | Male | Stage IB | Dead | | C | C |
| GSM1672557 | 61 | Male | Stage IIB | Dead | | C | C |
| GSM1672558 | 63 | Male | Stage II | Dead | | A | C |
| GSM1672559 | 62 | Male | Stage II | Dead | | B | C |
| GSM1672560 | 51 | Female | Stage IB | Alive | | C | C |
| GSM1672561 | 53 | Female | Stage IB | Alive | | C | B |
| GSM1672562 | 56 | Female | Stage II | Dead | | B | C |
| GSM1672563 | 61 | Male | Stage IIB | Dead | | C | B |
| GSM1672564 | 52 | Female | Stage IB | Dead | | C | A |
| GSM1672565 | 60 | Male | Stage IB | Alive | | B | A |
| GSM1672566 | 57 | Male | Stage II | Alive | | C | C |
| GSM1672567 | 46 | Female | Stage IB | Alive | | B | B |
| GSM1672568 | 71 | Male | Stage II | Dead | | C | B |
| GSM1672569 | 53 | Male | Stage II | Alive | | B | A |
| GSM1672570 | 68 | Male | Stage IIB | Alive | | A | C |
| GSM1672571 | 72 | Female | Stage II | Dead | | C | C |
| GSM1672572 | 68 | Female | Stage IA | Dead | | A | C |
| GSM1672573 | 65 | Male | Stage II | Alive | | C | A |
| GSM1672574 | 68 | Female | Stage IB | Alive | | C | C |
| GSM1672575 | 68 | Male | Stage IA | Alive | | C | A |
| GSM1672576 | 57 | Female | Stage IB | Alive | | C | C |
| GSM1672577 | 56 | Female | Stage IA | Alive | | C | A |
| GSM1672578 | 56 | Male | Stage IB | Alive | | A | B |
| GSM1672579 | 51 | Female | Stage IA | Alive | | A | A |
| GSM1672580 | 68 | Female | Stage IA | Dead | | A | A |
| GSM1672581 | 61 | Male | Stage IB | Alive | | A | A |
| GSM1672582 | 62 | Female | Stage IA | Dead | | C | A |
| GSM1672583 | 73 | Male | Stage IB | Alive | | B | A |
| GSM1672584 | 63 | Female | Stage II | Dead | | B | B |
| GSM1672585 | 72 | Male | Stage IB | Dead | | C | C |
| GSM1672586 | 57 | Female | Stage IA | Alive | | A | C |
| GSM1672587 | 55 | Female | Stage IA | Alive | | B | C |
| GSM1672588 | 64 | Male | Stage IB | Alive | | B | C |
| GSM1672589 | 40 | Female | Stage IB | Alive | | C | B |
| GSM1672590 | 75 | Female | Stage IIB | Dead | | B | A |
| GSM1672591 | 60 | Male | Stage IB | Alive | | A | C |
| GSM1672592 | 58 | Female | Stage IB | Alive | | B | A |
| GSM1672593 | 64 | Male | Stage IB | Alive | | C | C |
| GSM1672594 | 62 | Male | Stage IB | Alive | | B | C |
| GSM1672595 | 75 | Male | Stage IA | Alive | | C | C |
| GSM1672596 | 76 | Male | Stage IB | Dead | | C | A |
| GSM1672597 | 59 | Female | Stage IIB | Dead | | B | C |
| GSM1672598 | 54 | Male | Stage IA | Alive | | A | A |
| GSM1672599 | 74 | Male | Stage II | Dead | | A | A |
| GSM1672600 | 70 | Female | Stage IB | Alive | | B | C |
| GSM1672601 | 68 | Female | Stage IB | Alive | | A | C |
| GSM1672602 | 69 | Male | Stage IIB | Alive | | C | C |
| GSM1672603 | 75 | Male | Stage IB | Alive | | A | C |
| GSM1672604 | 64 | Male | Stage IB | Alive | | C | C |
| GSM1672605 | 79 | Male | Stage II | Dead | | A | C |
| GSM1672606 | 69 | Male | Stage IB | Dead | | B | B |
| GSM1672607 | 77 | Male | Stage II | Dead | | A | A |
| GSM1672608 | 66 | Female | Stage IB | Dead | | A | A |
| GSM1672609 | 79 | Male | Stage IB | Dead | | A | B |
| GSM1672610 | 72 | Female | Stage IB | Dead | | A | A |
| GSM1672611 | 67 | Male | Stage IIIB | Dead | | B | A |
| GSM1672612 | 79 | Female | Stage IIIA | Dead | | A | C |
| GSM1672613 | 74 | Female | Stage IIIA | Dead | | A | A |
| GSM1672615 | 40 | Male | Stage IB | Alive | | C | B |
| GSM1672616 | 79 | Male | Stage IIB | Dead | | C | C |
| GSM1672617 | 75 | Female | Stage IIB | Dead | | A | B |
| GSM1672619 | 77 | Female | Stage IA | Dead | | C | C |
| GSM1672620 | 66 | Female | Stage IIIA | Dead | | A | C |
| GSM1672621 | 49 | Male | Stage IB | Dead | | B | A |
| GSM1672622 | 62 | Male | Stage II | Dead | | A | B |
| GSM1672623 | 66 | Male | Stage IB | Dead | | A | C |
| GSM1672624 | 87 | Female | Stage IA | Dead | | C | C |
| GSM1672625 | 62 | Female | Stage IIB | Dead | | B | A |
| GSM1672626 | 82 | Male | Stage IIB | Dead | | B | A |
| GSM1672627 | 77 | Male | Stage IB | Dead | | C | B |
| GSM1672628 | 61 | Female | Stage IB | Dead | | A | A |
| GSM1672629 | 81 | Male | Stage IA | Dead | | B | B |
| GSM1672630 | 72 | Female | Stage II | Alive | | C | A |
| GSM1672631 | 67 | Female | Stage IB | Dead | | C | C |
| GSM1672632 | 74 | Female | Stage IIIA | Dead | | C | A |
| GSM1672633 | 64 | Female | Stage IB | Dead | | A | B |
| GSM1672634 | 65 | Female | Stage IB | Dead | | C | A |
| GSM1672635 | 76 | Male | Stage IIB | Dead | | C | B |
| GSM1672636 | 66 | Female | Stage IB | Alive | | B | A |
| GSM1672637 | 70 | Male | Stage IA | Alive | | C | C |
| GSM1672638 | 66 | Female | Stage IB | Alive | | C | C |
| GSM1672639 | 70 | Male | Stage IB | Dead | | A | C |
| GSM1672640 | 63 | Male | Stage IB | Dead | | B | B |
| GSM1672641 | 68 | Female | Stage IB | Dead | | A | C |
| GSM1672642 | 76 | Male | Stage IB | Dead | | C | B |
| GSM1672643 | 49 | Male | Stage IIIA | Dead | | C | C |
| GSM1672644 | 61 | Male | Stage IIIA | Dead | | A | A |
| GSM1672646 | 60 | Male | Stage IIB | Dead | | A | C |
| GSM1672647 | 73 | Female | Stage IA | Dead | | A | A |
| GSM1672648 | 47 | Male | Stage IIB | Alive | | B | B |
| GSM1672649 | 64 | Male | Stage IB | Dead | | C | C |
| GSM1672650 | 66 | Female | Stage IA | Alive | | A | C |
| GSM1672651 | 48 | Male | Stage II | Alive | | C | C |
| GSM1672652 | 79 | Male | Stage IIIA | Dead | | C | B |
| GSM1672653 | 68 | Female | Stage IIIA | Dead | | A | C |
| GSM1672654 | 68 | Female | Stage IIIB | Dead | | B | A |
| GSM1672655 | 82 | Female | Stage IB | Dead | | A | C |
| GSM1672656 | 70 | Male | Stage IB | Alive | | A | B |
| GSM1672657 | 75 | Male | Stage IB | Alive | | A | C |
| GSM1672658 | 63 | Female | Stage IB | Dead | | A | C |
| GSM1672659 | 81 | Female | Stage IB | Dead | | A | C |
| GSM1672660 | 75 | Female | Stage IIB | Dead | | A | A |
| GSM1672661 | 60 | Female | Stage IB | Dead | | C | C |
| GSM1672662 | 60 | Female | Stage IB | Alive | | A | C |
| GSM1672663 | 71 | Female | Stage IB | Dead | | C | C |
| GSM1672664 | 73 | Male | Stage IB | Alive | | A | A |
| GSM1672665 | 77 | Male | Stage IB | Dead | | A | A |
| GSM1672666 | 50 | Male | Stage IIB | Dead | | C | C |
| GSM1672667 | 59 | Female | Stage II | Dead | | C | B |
| GSM1672668 | 70 | Male | Stage IA | Dead | | C | B |
| GSM1672669 | 58 | Male | Stage II | Alive | | A | C |
| GSM1672670 | 60 | Male | Stage IB | Alive | | C | B |
| GSM1672671 | 72 | Male | Stage II | Dead | | B | B |
| GSM1672672 | 62 | Female | Stage IB | Alive | | A | C |
| GSM1672673 | 77 | Female | Stage IA | Alive | | B | B |
| GSM1672674 | 82 | Male | Stage IB | Dead | | A | B |
| GSM1672675 | 73 | Male | Stage IB | Dead | | C | B |
| GSM1672676 | 57 | Female | Stage II | Dead | | C | B |
| GSM1672677 | 73 | Male | Stage II | Dead | | A | B |
| GSM1672678 | 70 | Female | Stage IIIA | Dead | | B | A |
| GSM1672679 | 53 | Male | Stage IB | Alive | | C | C |
| GSM1672680 | 75 | Male | Stage IIIB | Dead | | C | C |
| GSM1672681 | 71 | Female | Stage II | Dead | | A | A |
| GSM1672682 | 36 | Male | Stage II | Dead | | A | B |
| GSM1672683 | 68 | Female | Stage IIIA | Dead | | A | B |
| GSM1672684 | 68 | Female | Stage IIB | Dead | | A | A |
| GSM1672685 | 65 | Male | Stage IIIA | Dead | | A | B |
| GSM1672686 | 51 | Female | Stage IIIA | Dead | | A | C |
| GSM1672687 | 62 | Male | Stage IA | Dead | | C | C |
| GSM1672688 | 72 | Male | Stage IIB | Dead | | A | C |
| GSM1672689 | 69 | Male | Stage IIIA | Dead | | A | A |
| GSM1672690 | 68 | Male | Stage IIIA | Dead | | C | C |
| GSM1672691 | 57 | Male | Stage IIB | Dead | | A | C |
| GSM1672692 | 69 | Male | Stage IB | Dead | | A | A |
| GSM1672693 | 58 | Male | Stage IA | Dead | | A | B |
| GSM1672694 | 72 | Male | Stage IIB | Dead | | A | A |
| GSM1672695 | 47 | Male | Stage IB | Dead | | C | C |
| GSM1672696 | 64 | Male | Stage IIIA | Dead | | C | A |
| GSM1672697 | 59 | Male | Stage IIB | Alive | | A | A |
| GSM1672698 | 69 | Male | Stage IA | Dead | | A | A |
| GSM1672699 | 77 | Male | Stage IIB | Dead | | A | A |
| GSM1672700 | 77 | Male | Stage IA | Dead | | A | C |
| GSM1672701 | 70 | Male | Stage II | Dead | | C | A |
| GSM1672702 | 57 | Male | Stage IIIA | Dead | | A | A |
| GSM1672703 | 67 | Male | Stage IB | Alive | | A | A |
| GSM1672704 | 66 | Male | Stage IIIA | Dead | | A | C |
| GSM1672705 | 78 | Male | Stage IB | Dead | | A | C |
| GSM1672706 | 46 | Male | Stage IB | Dead | | C | A |
| GSM1672707 | 62 | Male | Stage IA | Dead | | A | A |
| GSM1672708 | 66 | Male | Stage IB | Dead | | A | B |
| GSM1672709 | 65 | Male | Stage II | Dead | | A | A |
| GSM1672710 | 46 | Female | Stage IIB | Alive | | C | C |
| GSM1672711 | 63 | Female | Stage IIB | Alive | | A | C |
| GSM1672712 | 60 | Male | Stage IIB | Dead | | C | C |
| GSM1672713 | 77 | Male | Stage IB | Alive | | C | A |
| GSM1672714 | 44 | Male | Stage IIIA | Dead | | B | A |
| GSM1672715 | 49 | Male | Stage IIIB | Dead | | A | A |
| GSM1672716 | 70 | Male | Stage IIIA | Dead | | A | A |
| GSM1672717 | 68 | Male | Stage IA | Dead | | A | A |
| GSM1672718 | 66 | Male | Stage IB | Dead | | A | A |
| GSM1672719 | 74 | Male | Stage IIIA | Alive | | A | C |
| GSM1672720 | 62 | Male | Stage IIIA | Dead | | C | C |
| GSM1672721 | 81 | Male | Stage IB | Alive | | C | C |
| GSM1672722 | 65 | Male | Stage IIB | Alive | | B | B |
| GSM1672723 | 61 | Male | Stage II | Dead | | A | B |
| GSM1675665 | 65 | Male | Stage I | Dead | | C | C |
| GSM1675666 | 66 | Female | Stage I | Alive | | B | A |
| GSM1675667 | 60 | Female | Stage I | Alive | | C | B |
| GSM1675668 | 62 | Female | Stage I | Alive | | A | C |
| GSM1675669 | 66 | Male | Stage I | Dead | | C | A |
| GSM1675670 | 56 | Male | Stage I | Alive | | A | C |
| GSM1675671 | 75 | Male | Stage I | Alive | | C | A |
| GSM1675672 | 77 | Male | Stage I | Alive | | A | B |
| GSM1675673 | 63 | Male | Stage I | Alive | | C | A |
| GSM1675674 | 52 | Male | Stage I | Dead | | B | A |
| GSM1675675 | 55 | Female | Stage I | Alive | | C | C |
| GSM1675676 | 58 | Female | Stage I | Alive | | A | A |
| GSM1675677 | 60 | Female | Stage I | Alive | | C | B |
| GSM1675678 | 48 | Female | Stage I | Alive | | C | A |
| GSM1675679 | 73 | Female | Stage I | Alive | | A | C |
| GSM1675680 | 46 | Female | Stage I | Alive | | A | C |
| GSM1675681 | 75 | Female | Stage I | Alive | | B | B |
| GSM1675682 | 85 | Female | Stage I | Alive | | C | C |
| GSM1675683 | 69 | Female | Stage I | Alive | | C | C |
| GSM1675684 | 74 | Female | Stage I | Alive | | A | C |
| GSM1675685 | 83 | Female | Stage I | Alive | | C | C |
| GSM1675686 | 59 | Female | Stage I | Alive | | A | A |
| GSM1675687 | 61 | Female | Stage I | Alive | | C | C |
| GSM1675688 | 68 | Female | Stage I | Dead | | A | A |
| GSM1675689 | 55 | Female | Stage I | Alive | | A | C |
| GSM1675690 | 45 | Female | Stage I | Alive | | A | B |
| GSM1675691 | 67 | Male | Stage I | Dead | | C | A |
| GSM1675692 | 41 | Female | Stage I | Alive | | C | A |
| GSM1675693 | 83 | Female | Stage I | Alive | | A | C |
| GSM1675694 | 57 | Male | Stage III | Dead | | C | C |
| GSM1675695 | 80 | Male | Stage I | Dead | | A | A |
| GSM1675696 | 66 | Male | Stage I | Alive | | C | A |
| GSM1675697 | 62 | Male | Stage III | Alive | | A | A |
| GSM1675698 | 85 | Female | Stage I | Alive | | A | C |
| GSM1675699 | 63 | Female | Stage I | Alive | | A | C |
| GSM1675700 | 61 | Male | Stage I | Alive | | A | C |
| GSM1675701 | 70 | Male | Stage I | Alive | | B | B |
| GSM1675702 | 52 | Female | Stage I | Alive | | B | C |
| GSM1675703 | 62 | Female | Stage I | Alive | | C | A |
| GSM1675704 | 54 | Female | Stage III | Alive | | C | C |
| GSM1675705 | 77 | Male | Stage III | Dead | | C | C |
| GSM1675706 | 64 | Male | Stage III | Dead | | B | A |
| GSM1675707 | 70 | Male | Stage III | Dead | | A | A |
| GSM1675708 | 64 | Male | Stage III | Dead | | C | B |
| GSM1675709 | 79 | Female | Stage III | Alive | | C | C |
| GSM1675710 | 55 | Female | Stage III | Dead | | C | C |
| GSM1675711 | 73 | Female | Stage I | Dead | | B | A |
| GSM1675712 | 76 | Female | Stage I | Alive | | C | C |
| GSM1675713 | 51 | Female | Stage I | Alive | | A | B |
| GSM1675714 | 75 | Female | Stage I | Dead | | A | A |
| GSM1675715 | 60 | Male | Stage I | Alive | | A | C |
| GSM1675716 | 60 | Male | Stage I | Alive | | C | B |
| GSM1675717 | 43 | Male | Stage I | Alive | | A | A |
| GSM1675718 | 66 | Female | Stage I | Alive | | A | C |
| GSM1675719 | 72 | Male | Stage I | Dead | | C | A |
| GSM1675720 | 67 | Male | Stage I | Alive | | A | C |
| GSM1675721 | 59 | Female | Stage III | Dead | | B | A |
| GSM1675722 | 46 | Female | Stage III | Dead | | A | A |
| GSM1675723 | 60 | Male | Stage I | Alive | | C | C |
| GSM1675724 | 74 | Female | Stage I | Alive | | A | C |
| GSM1675725 | 72 | Female | Stage III | Alive | | B | B |
| GSM1675726 | 63 | Female | Stage I | Dead | | B | B |
| GSM1675727 | 52 | Female | Stage III | Alive | | B | C |
| GSM1675728 | 65 | Male | Stage I | Alive | | A | C |
| GSM1675729 | 60 | Male | Stage I | Alive | | A | C |
| GSM1675730 | 46 | Male | Stage I | Alive | | C | B |
| GSM1675731 | 76 | Female | Stage I | Alive | | A | C |
| GSM1675732 | 49 | Female | Stage I | Dead | | C | B |
| GSM1675733 | 68 | Female | Stage I | Dead | | C | B |
| GSM1675734 | 58 | Male | Stage I | Alive | | B | B |
| GSM1675735 | 69 | Female | Stage I | Alive | | C | C |
| GSM1675736 | 62 | Female | Stage I | Alive | | C | C |
| GSM1675737 | 67 | Female | Stage I | Alive | | A | C |
| GSM1675738 | 60 | Male | Stage I | Alive | | C | B |
| GSM1675739 | 63 | Female | Stage I | Alive | | A | C |
| GSM1675740 | 66 | Male | Stage I | Alive | | A | C |
| GSM1675741 | 53 | Female | Stage I | Alive | | A | C |
| GSM1675742 | 59 | Male | Stage III | Alive | | B | A |
| GSM1675743 | 64 | Female | Stage I | Dead | | B | C |
| GSM1675744 | 64 | Male | Stage III | Alive | | C | B |
| GSM1675745 | 55 | Male | Stage III | Alive | | B | B |
| GSM1675746 | 72 | Male | Stage III | Dead | | B | B |
| GSM1675747 | 72 | Female | Stage III | Dead | | A | B |
| GSM1675748 | 64 | Female | Stage III | Dead | | B | A |
| GSM1675749 | 64 | Female | Stage I | Alive | | A | C |
| GSM1675750 | 74 | Male | Stage I | Alive | | A | A |
| GSM1854798 | 70 | Female | Stage IV | Alive | | A | C |
| GSM1854799 | 59 | Female | Stage IIB | Alive | | B | A |
| GSM1854800 | 57 | Male | Stage IB | Alive | | A | C |
| GSM1854801 | 72 | Female | Stage IB | Alive | | A | C |
| GSM1854802 | 73 | Female | Stage IA | Dead | | A | C |
| GSM1854803 | 49 | Female | Stage IA | Alive | | A | A |
| GSM1854804 | 70 | Male | Stage I | Alive | | A | A |
| GSM1854805 | 71 | Male | Stage IA | Alive | | A | A |
| GSM1854806 | 66 | Male | Stage IB | Alive | | C | C |
| GSM1854807 | 68 | Male | Stage IIB | Dead | | C | C |
| GSM1854808 | 86 | Male | Stage IA | Alive | | B | C |
| GSM1854809 | 65 | Female | Stage IA | Alive | | A | C |
| GSM1854810 | 78 | Male | Stage IA | Alive | | A | C |
| GSM1854811 | 85 | Female | Stage IIB | Alive | | A | C |
| GSM1854812 | 71 | Female | Stage IA | Alive | | A | A |
| GSM1854813 | 66 | Male | Stage IA | Alive | | C | C |
| GSM1854814 | 44 | Male | Stage IIB | Alive | | A | C |
| GSM1854815 | 73 | Female | Stage IIIB | Alive | | C | A |
| GSM1854816 | 50 | Male | Stage IIIA | Alive | | C | B |
| GSM1854817 | 80 | Male | Stage IA | Alive | | A | C |
| GSM1854818 | 63 | Female | Stage IA | Alive | | A | A |
| GSM1854819 | 67 | Male | Stage IIIA | Alive | | A | A |
| GSM1854820 | 72 | Female | Stage IA | Alive | | C | B |
| GSM1854821 | 83 | Male | Stage IIB | Alive | | C | C |
| GSM1854822 | 65 | Male | Stage IB | Alive | | C | B |
| GSM1854823 | 57 | Female | Stage IIB | Alive | | C | A |
| GSM1854824 | 67 | Female | Stage IIB | Alive | | C | C |
| GSM1854825 | 54 | Female | Stage IA | Alive | | C | C |
| GSM1854826 | 69 | Male | Stage IB | Alive | | A | C |
| GSM1854827 | 66 | Female | Stage IB | Alive | | A | A |
| GSM1854828 | 73 | Female | Stage IIIB | Alive | | A | C |
| GSM1854829 | 69 | Male | Stage IIIA | Alive | | B | A |
| GSM1854830 | 69 | Female | Stage IIIB | Alive | | A | C |
| GSM1854831 | 77 | Male | Stage IIIA | Dead | | B | B |
| GSM1854832 | 63 | Female | Stage IA | Alive | | A | C |
| GSM1854833 | 79 | Male | Stage IIB | Alive | | A | C |
| GSM1854834 | 66 | Female | Stage IB | Alive | | C | C |
| GSM1854835 | 44 | Female | Stage IA | Alive | | A | A |
| GSM1854836 | 74 | Male | Stage IA | Dead | | A | A |
| GSM1854837 | 78 | Female | Stage IIB | Dead | | C | A |
| GSM1854838 | 50 | Female | Stage IB | Dead | | C | A |
| GSM1854839 | 70 | Female | Stage IV | Alive | | C | C |
| GSM1854840 | 72 | Male | Stage IA | Dead | | A | B |
| GSM1854841 | 65 | Female | Stage IB | Alive | | C | B |
| GSM1854842 | 75 | Female | Stage IIIA | Dead | | C | C |
| GSM1854843 | 66 | Male | Stage IB | Alive | | C | C |
| GSM1854844 | 52 | Female | Stage IIIA | Dead | | C | B |
| GSM1854845 | 63 | Female | Stage IB | Dead | | A | C |
| GSM1854846 | 80 | Female | Stage IA | Alive | | B | B |
| GSM1854847 | 56 | Male | Stage IA | Alive | | C | C |
| GSM1854848 | 61 | Female | Stage IA | Alive | | A | A |
| GSM1854849 | 77 | Male | Stage IB | Alive | | C | C |
| GSM1854850 | 86 | Male | Stage IA | Alive | | B | C |
| GSM1854851 | 63 | Female | Stage I | Alive | | C | C |
| GSM1854852 | 76 | Female | Stage IA | Alive | | A | C |
| GSM1854853 | 67 | Male | Stage IA | Alive | | A | B |
| GSM1854854 | 64 | Male | Stage IA | Alive | | A | C |
| GSM1854855 | 84 | Male | Stage IB | Dead | | C | A |
| GSM1854856 | 78 | Female | Stage IIIA | Dead | | C | A |
| GSM1854857 | 75 | Female | Stage IB | Alive | | A | C |
| GSM1854858 | 73 | Male | Stage IIB | Dead | | A | A |
| GSM1854859 | 74 | Female | Stage IIIA | Dead | | A | B |
| GSM1854860 | 81 | Male | Stage IV | Dead | | B | A |
| GSM1854861 | 67 | Male | Stage IIIB | Dead | | C | C |
| GSM1854862 | 73 | Female | Stage IA | Alive | | A | C |
| GSM1854863 | 71 | Female | Stage IB | Alive | | A | C |
| GSM1854864 | 79 | Female | Stage IIIB | Dead | | C | C |
| GSM1854865 | 62 | Male | Stage IIB | Alive | | C | C |
| GSM1854866 | 72 | Female | Stage IB | Alive | | C | B |
| GSM1854867 | 64 | Male | Stage IB | Alive | | C | C |
| GSM1854868 | 66 | Male | Stage IIB | Dead | | B | A |
| GSM1854869 | 65 | Male | Stage IA | Alive | | B | C |
| GSM1854870 | 47 | Male | Stage IA | Alive | | C | C |
| GSM1854871 | 78 | Female | Stage IIB | Alive | | A | C |
| GSM1854872 | 76 | Female | Stage IA | Alive | | B | A |
| GSM1854873 | 68 | Male | Stage IA | Alive | | C | A |
| GSM1854874 | 72 | Female | Stage IIA | Dead | | C | C |
| GSM1854875 | 64 | Male | Stage IIB | Alive | | A | C |
| GSM1854876 | 74 | Female | Stage IIB | Alive | | B | B |
| GSM1854877 | 83 | Female | Stage IA | Alive | | B | B |
| GSM1854879 | 81 | Female | Stage IV | Alive | | C | C |
| GSM1854880 | 80 | Female | Stage IA | Alive | | A | B |
| GSM1854881 | 76 | Male | Stage IIB | Dead | | C | B |
| GSM1854882 | 70 | Female | Stage IA | Alive | | A | C |
| GSM1854883 | 62 | Female | Stage IIIB | Dead | | A | C |
| GSM1854884 | 64 | Male | Stage IIIA | Dead | | A | A |
| GSM1854885 | 82 | Male | Stage IB | Dead | | B | B |
| GSM1854886 | 59 | Male | Stage IIIA | Dead | | B | B |
| GSM1854887 | 78 | Female | Stage IA | Alive | | B | B |
| GSM1854888 | 69 | Male | Stage IA | Alive | | C | C |
| GSM1854889 | 67 | Male | Stage IIIA | Dead | | B | B |
| GSM1854890 | 68 | Male | Stage IIB | Alive | | C | B |
| GSM1854891 | 71 | Male | Stage IIIB | Dead | | C | A |
| GSM1854892 | 85 | Male | Stage IIB | Dead | | B | A |
| GSM1854893 | 47 | Female | Stage IIB | Dead | | C | A |
| GSM1854895 | 74 | Female | Stage IIIA | Dead | | B | A |
| GSM1854896 | 75 | Male | Stage IIIB | Dead | | B | A |
| GSM1854897 | 68 | Male | Stage IA | Dead | | C | C |
| GSM1854898 | 64 | Male | Stage IA | Alive | | A | C |
| GSM1854899 | 73 | Male | Stage IV | Dead | | B | B |
| GSM1854902 | 75 | Male | Stage IA | Alive | | C | C |
| GSM1854903 | 74 | Female | Stage IIIB | Dead | | B | A |
| GSM1854904 | 62 | Female | Stage IB | Alive | | A | C |
| GSM1854906 | 61 | Female | Stage IA | Alive | | A | C |
| GSM1854907 | 70 | Male | Stage IA | Alive | | B | B |
| GSM1854908 | 73 | Female | Stage IA | Alive | | A | A |
| GSM1854909 | 81 | Female | Stage I | Alive | | A | C |
| GSM1854910 | 77 | Female | Stage IA | Alive | | A | C |
| GSM1854911 | 69 | Male | Stage IB | Dead | | A | C |
| GSM1854913 | 73 | Female | Stage IB | Alive | | C | B |
| GSM1854915 | 77 | Female | Stage IA | Alive | | A | A |
| GSM1854916 | 78 | Male | Stage IA | Alive | | B | B |
| GSM1854917 | 75 | Female | Stage IA | Alive | | A | C |
| GSM1854918 | 72 | Female | Stage IIA | Alive | | C | C |
| GSM1854919 | 72 | Female | Stage IA | Alive | | C | C |
| GSM1854920 | 85 | Female | Stage IB | Alive | | A | C |
| GSM1854921 | 76 | Male | Stage IIA | Dead | | A | A |
| GSM1854922 | 58 | Female | Stage IA | Alive | | A | C |
| GSM1854923 | 79 | Female | Stage IB | Alive | | A | C |
| GSM1854924 | 67 | Male | Stage IA | Alive | | C | C |
| GSM1854925 | 63 | Female | Stage IV | Alive | | C | B |
| GSM1854926 | 67 | Female | Stage IIIA | Alive | | A | C |
| GSM1854927 | 83 | Male | Stage IA | Dead | | B | A |
| GSM1854928 | 63 | Male | Stage IB | Alive | | C | C |
| GSM1854929 | 62 | Male | Stage IB | Alive | | C | A |
| GSM1854930 | 76 | Male | Stage IB | Alive | | C | A |
| GSM1854931 | 76 | Female | Stage IB | Alive | | A | C |
| GSM1854932 | 59 | Female | Stage IIB | Alive | | C | A |
| GSM1854933 | 74 | Male | Stage IA | Alive | | C | C |
| GSM1854934 | 72 | Male | Stage IB | Alive | | A | C |
| GSM1854935 | 79 | Male | Stage IIB | Dead | | B | A |
| GSM1854936 | 66 | Female | Stage IB | Alive | | C | C |
| GSM1854937 | 63 | Male | Stage IIA | Alive | | B | A |
| GSM1854938 | 60 | Female | Stage IIB | Alive | | A | A |
| GSM1854939 | 76 | Female | Stage IIIB | Alive | | A | A |
| GSM1854940 | 57 | Female | Stage IA | Alive | | B | A |
| GSM1854941 | 87 | Female | Stage IV | Alive | | C | C |
| GSM1854942 | 68 | Male | Stage IIIA | Alive | | C | C |
| GSM1854944 | 77 | Female | Stage IB | Alive | | A | A |
| GSM1854945 | 68 | Female | Stage IIIA | Alive | | B | A |
| GSM1854946 | 60 | Female | Stage IA | Alive | | B | C |
| GSM1854947 | 70 | Male | Stage IIB | Dead | | B | A |
| GSM1854948 | 74 | Female | Stage IA | Dead | | A | C |
| GSM1854949 | 76 | Male | Stage IIIA | Dead | | A | C |
| GSM1854950 | 72 | Male | Stage IB | Alive | | A | C |
| GSM1854951 | 70 | Female | Stage IA | Dead | | A | C |
| GSM1854952 | 72 | Female | Stage IIA | Alive | | C | C |
| GSM1854953 | 55 | Male | Stage IA | Alive | | C | C |
| GSM1854954 | 84 | Male | Stage IV | Alive | | C | A |
| GSM1854955 | 72 | Female | Stage IIIA | Alive | | B | C |
| GSM1854956 | 77 | Male | Stage IA | Dead | | C | C |
| GSM1854957 | 77 | Female | Stage IIA | Dead | | A | C |
| GSM1854958 | 79 | Female | Stage IA | Alive | | A | C |
| GSM1854960 | 60 | Male | Stage IIB | Alive | | A | A |
| GSM1854961 | 70 | Female | Stage IV | Dead | | A | C |
| GSM1854962 | 74 | Male | Stage IIIA | Alive | | A | C |
| GSM1854963 | 72 | Female | Stage IIA | Dead | | C | A |
| GSM1854964 | 78 | Male | Stage IA | Alive | | B | A |
| GSM1854965 | 71 | Female | Stage IA | Dead | | A | A |
| GSM1854966 | 84 | Male | Stage IV | Dead | | A | C |
| GSM1854967 | 78 | Male | Stage IB | Alive | | B | A |
| GSM1854969 | 71 | Female | Stage IA | Alive | | A | C |
| GSM1854970 | 64 | Male | Stage IB | Alive | | A | C |
| GSM1854971 | 83 | Male | Stage IB | Alive | | B | A |
| GSM1854972 | 64 | Female | Stage IIIB | Alive | | A | C |
| GSM1854973 | 57 | Male | Stage IA | Alive | | C | C |
| GSM1854974 | 83 | Male | Stage IIB | Alive | | C | C |
| GSM1854975 | 78 | Male | Stage IB | Alive | | A | B |
| GSM1854977 | 66 | Male | Stage IB | Alive | | A | C |
| GSM1854978 | 72 | Male | Stage IA | Dead | | C | A |
| GSM1854979 | 67 | Male | Stage IB | Alive | | C | C |
| GSM1854980 | 88 | Female | Stage IA | Alive | | C | C |
| GSM1854981 | 79 | Female | Stage IIA | Dead | | C | C |
| GSM1854982 | 58 | Female | Stage I | Alive | | A | C |
| GSM1854983 | 81 | Male | Stage IB | Dead | | C | B |
| GSM1854984 | 82 | Male | Stage IA | Alive | | C | B |
| GSM1854985 | 79 | Female | Stage IIIA | Alive | | A | C |
| GSM1854986 | 77 | Male | Stage IB | Alive | | A | A |
| GSM1854987 | 47 | Female | Stage IA | Alive | | C | C |
| GSM1854988 | 64 | Female | Stage IA | Alive | | A | A |
| GSM1854989 | 67 | Male | Stage IA | Alive | | C | C |
| GSM1854990 | 76 | Female | Stage IA | Alive | | C | C |
| GSM1854991 | 65 | Female | Stage IV | Alive | | A | C |
| GSM1854992 | 70 | Female | Stage IB | Alive | | A | A |
| GSM1854994 | 75 | Female | Stage IA | Alive | | A | C |
| GSM1854995 | 49 | Female | Stage IA | Dead | | B | A |
| GSM1854996 | 69 | Female | Stage IB | Dead | | C | A |
| GSM1854997 | 79 | Male | Stage IIIB | Dead | | B | A |
| GSM1854998 | 61 | Female | Stage IIIA | Dead | | B | A |
| GSM1854999 | 77 | Female | Stage IB | Alive | | A | C |
| GSM1855000 | 50 | Female | Stage IA | Alive | | A | A |
| GSM1855001 | 62 | Female | Stage IIB | Dead | | A | B |
| GSM1855003 | 75 | Female | Stage IB | Alive | | A | C |
| GSM1855004 | 71 | Male | Stage IA | Alive | | C | C |
| GSM1855005 | 59 | Female | Stage IIIA | Alive | | C | C |
| GSM1855006 | 79 | Male | Stage IIB | Alive | | C | A |
| GSM1855007 | 78 | Male | Stage IA | Alive | | C | C |
| GSM1855008 | 66 | Female | Stage IA | Alive | | A | A |
| GSM1855009 | 61 | Female | Stage IIIA | Alive | | C | C |
| GSM1855010 | 64 | Male | Stage IA | Alive | | C | A |
| GSM1855011 | 52 | Female | Stage IA | Alive | | A | C |
| GSM1855012 | 74 | Female | Stage IB | Alive | | B | A |
| GSM1855014 | 68 | Male | Stage IA | Dead | | B | A |
| GSM1855015 | 75 | Female | Stage IB | Alive | | C | C |
| GSM1855016 | 60 | Male | Stage IA | Alive | | C | A |
| GSM1855017 | 64 | Female | Stage IV | Dead | | C | C |
| GSM1855018 | 68 | Female | Stage IB | Alive | | A | A |
| GSM1855019 | 73 | Male | Stage IA | Dead | | C | A |
| GSM1855020 | 77 | Female | Stage IA | Alive | | A | C |
| GSM1855021 | 70 | Female | Stage IB | Alive | | A | C |
| GSM1855022 | 70 | Male | Stage IB | Alive | | B | B |
| GSM1855023 | 59 | Female | Stage IIB | Alive | | C | C |
| GSM1855024 | 82 | Male | Stage IIA | Alive | | B | A |
| GSM1855025 | 68 | Male | Stage IA | Alive | | B | A |
| GSM1855026 | 47 | Female | Stage IA | Alive | | A | B |
| GSM1855027 | 61 | Female | Stage IB | Dead | | A | B |
| GSM1855028 | 75 | Female | Stage IA | Alive | | C | B |
| GSM1855029 | 70 | Male | Stage IA | Dead | | B | A |
| GSM1855031 | 71 | Male | Stage IA | Alive | | A | C |
| GSM1855032 | 73 | Female | Stage IB | Alive | | C | C |
| GSM1855033 | 65 | Female | Stage IB | Alive | | C | A |
| GSM1855034 | 53 | Female | Stage IA | Alive | | C | C |
| GSM1855035 | 69 | Female | Stage IA | Alive | | C | A |
| GSM1855036 | 63 | Male | Stage IA | Dead | | A | C |
| GSM1855037 | 38 | Female | Stage IB | Alive | | C | B |
| GSM1855038 | 62 | Female | Stage IB | Alive | | C | B |
| GSM1855039 | 69 | Male | Stage IIB | Dead | | B | A |
| GSM1855040 | 70 | Male | Stage IA | Alive | | C | C |
| GSM1855041 | 68 | Male | Stage IA | Alive | | C | B |
| GSM1855042 | 53 | Female | Stage IA | Alive | | A | C |
| GSM1855043 | 70 | Male | Stage IB | Alive | | C | B |
| GSM1855044 | 65 | Female | Stage IA | Alive | | A | C |
| GSM1855045 | 67 | Male | Stage IA | Alive | | A | C |
| GSM1855046 | 78 | Male | Stage IA | Alive | | C | C |
| GSM1855047 | 73 | Female | Stage IIB | Alive | | B | B |
| GSM1855048 | 77 | Male | Stage IB | Dead | | C | B |
| GSM1855049 | 57 | Female | Stage IA | Dead | | A | A |
| GSM1855050 | 63 | Female | Stage IB | Alive | | A | C |
| GSM1855051 | 55 | Female | Stage IA | Alive | | A | A |
| GSM1855052 | 79 | Male | Stage IA | Dead | | B | A |
| GSM1855053 | 72 | Female | Stage IB | Alive | | C | C |
| GSM1855054 | 67 | Female | Stage IA | Alive | | A | C |
| GSM1855055 | 70 | Female | Stage IA | Alive | | A | B |
| GSM1855056 | 67 | Female | Stage IB | Alive | | C | C |
| GSM1855057 | 70 | Female | Stage IIA | Alive | | A | C |
| GSM1855059 | 71 | Male | Stage IB | Alive | | C | C |
| GSM1855060 | 64 | Female | Stage IB | Alive | | C | C |
| GSM1855061 | 60 | Female | Stage IV | Dead | | C | C |
| GSM1855062 | 72 | Female | Stage IIB | Dead | | A | A |
| GSM1855063 | 66 | Male | Stage IIIA | Dead | | C | A |
| GSM1855064 | 69 | Male | Stage IB | Alive | | A | A |
| GSM1855065 | 81 | Male | Stage IIB | Alive | | C | A |
| GSM1855066 | 80 | Female | Stage IIB | Alive | | A | B |
| GSM1855067 | 75 | Male | Stage IB | Alive | | B | A |
| GSM1855068 | 70 | Male | Stage IA | Dead | | C | C |
| GSM1855069 | 58 | Male | Stage IIA | Alive | | C | C |
| GSM1855070 | 71 | Female | Stage IIIA | Alive | | A | C |
| GSM1855071 | 80 | Female | Stage IA | Alive | | A | A |
| GSM1855072 | 86 | Female | Stage IB | Alive | | A | C |
| GSM1855073 | 60 | Female | Stage IA | Alive | | C | C |
| GSM1855074 | 65 | Male | Stage IA | Alive | | C | B |
| GSM1855075 | 77 | Male | Stage IIA | Alive | | C | C |
| GSM1855076 | 60 | Female | Stage IIIA | Alive | | A | C |
| GSM1855077 | 75 | Male | Stage IB | Alive | | C | C |
| GSM1855078 | 62 | Female | Stage IA | Alive | | B | B |
| GSM1855079 | 66 | Male | Stage IB | Dead | | B | A |
| GSM1855080 | 80 | Female | Stage IIB | Alive | | C | A |
| GSM1855081 | 78 | Female | Stage IA | Alive | | A | A |
| GSM1855082 | 60 | Female | Stage IA | Alive | | C | C |
| GSM1855083 | 56 | Male | Stage IIIA | Alive | | B | B |
| GSM1855084 | 75 | Male | Stage IA | Alive | | C | C |
| GSM1855085 | 60 | Female | Stage IA | Alive | | C | A |
| GSM1855086 | 82 | Male | Stage IB | Alive | | A | A |
| GSM1855087 | 77 | Male | Stage IB | Alive | | B | A |
| GSM1855088 | 70 | Female | Stage I | Alive | | C | C |
| GSM1855089 | 78 | Female | Stage IB | Alive | | B | A |
| GSM1855091 | 79 | Male | Stage IB | Dead | | B | A |
| GSM1855092 | 78 | Female | Stage IB | Alive | | A | C |
| GSM1855093 | 72 | Male | Stage IA | Alive | | A | C |
| GSM1855094 | 66 | Female | Stage IB | Alive | | C | C |
| GSM1855095 | 81 | Male | Stage IA | Alive | | A | A |
| GSM1855096 | 74 | Female | Stage IIIB | Alive | | C | C |
| GSM1855097 | 66 | Female | Stage IIIA | Alive | | A | C |
| GSM1855098 | 83 | Female | Stage IB | Alive | | A | C |
| GSM1855099 | 81 | Male | Stage IB | Dead | | C | C |
| GSM1855100 | 67 | Female | Stage IB | Alive | | C | B |
| GSM1855101 | 55 | Female | Stage IIIA | Dead | | A | C |
| GSM1855102 | 73 | Female | Stage IIIA | Alive | | C | C |
| GSM1855103 | 69 | Male | Stage IB | Alive | | C | B |
| GSM1855105 | 68 | Female | Stage IA | Alive | | B | C |
| GSM1855106 | 84 | Male | Stage IA | Dead | | B | C |
| GSM1855107 | 76 | Female | Stage IA | Alive | | B | A |
| GSM1855108 | 61 | Female | Stage IIIA | Alive | | B | B |
| GSM1855109 | 62 | Male | Stage IA | Dead | | C | A |
| GSM1855110 | 61 | Male | Stage IIA | Alive | | A | C |
| GSM1855112 | 69 | Female | Stage IA | Alive | | A | C |
| GSM1855113 | 77 | Male | Stage IA | Alive | | A | C |
| GSM1855114 | 58 | Male | Stage IA | Alive | | A | B |
| GSM1855116 | 76 | Male | Stage IA | Alive | | C | A |
| GSM1855117 | 74 | Female | Stage IIIA | Alive | | B | B |
| GSM1855118 | 45 | Male | Stage IIIB | Alive | | A | B |
| GSM1855119 | 50 | Female | Stage IIIA | Alive | | B | A |
| GSM1855120 | 70 | Female | Stage IB | Dead | | B | A |
| GSM1855121 | 67 | Male | Stage IIIA | Dead | | A | B |
| GSM1855122 | 41 | Male | Stage IIA | Alive | | C | C |
| GSM1855123 | 84 | Female | Stage IA | Alive | | B | A |
| GSM1855124 | 51 | Male | Stage IB | Alive | | B | A |
| GSM1855125 | 74 | Male | Stage IB | Dead | | C | A |
| GSM1855126 | 66 | Female | Stage IIB | Alive | | A | A |
| GSM1855127 | 52 | Female | Stage IIIA | Alive | | A | C |
| GSM1855128 | 51 | Female | Stage IA | Alive | | A | A |
| GSM1855129 | 53 | Female | Stage IA | Alive | | A | C |
| GSM1855131 | 65 | Male | Stage IA | Dead | | A | A |
| GSM1855132 | 79 | Female | Stage IB | Alive | | A | A |
| GSM1855133 | 59 | Female | Stage IB | Alive | | B | C |
| GSM1855134 | 69 | Female | Stage IA | Dead | | A | C |
| GSM1855135 | 83 | Female | Stage IIIA | Alive | | A | A |
| GSM1855136 | 50 | Male | Stage IA | Dead | | B | A |
| GSM1855137 | 72 | Male | Stage IIIA | Alive | | A | A |
| GSM1855138 | 67 | Female | Stage IA | Alive | | A | C |
| GSM1855139 | 77 | Male | Stage IA | Alive | | A | B |
| GSM1855140 | 82 | Male | Stage IA | Alive | | C | A |
| GSM1855141 | 72 | Female | Stage IA | Alive | | A | C |
| GSM1855142 | 72 | Male | Stage IA | Alive | | A | A |
| GSM1855143 | 70 | Female | Stage IA | Alive | | A | C |
| GSM1855144 | 51 | Male | Stage IB | Alive | | C | C |
| GSM1855145 | 74 | Female | Stage IA | Dead | | A | A |
| GSM1855146 | 74 | Female | Stage IA | Alive | | A | A |
| GSM1855147 | 69 | Female | Stage IA | Alive | | C | C |
| GSM1855148 | 69 | Male | Stage IA | Alive | | C | A |
| GSM1855149 | 69 | Female | Stage IB | Alive | | C | C |
| GSM1855150 | 73 | Female | Stage IIB | Dead | | C | B |
| GSM1855151 | 76 | Female | Stage IA | Alive | | A | C |
| GSM1855152 | 77 | Male | Stage IA | Alive | | A | C |
| GSM1855153 | 66 | Male | Stage IB | Dead | | B | C |
| GSM1855155 | 62 | Male | Stage IA | Alive | | C | C |
| GSM1855156 | 66 | Female | Stage IIB | Alive | | A | A |
| GSM1855157 | 85 | Male | Stage IIB | Alive | | A | A |
| GSM1855158 | 81 | Male | Stage IIA | Alive | | A | C |
| GSM1855159 | 64 | Female | Stage IA | Alive | | A | C |
| GSM1855160 | 56 | Male | Stage IIA | Alive | | A | A |
| GSM1855161 | 66 | Male | Stage IB | Dead | | A | C |
| GSM1855162 | 77 | Female | Stage IB | Alive | | A | C |
| GSM1855163 | 58 | Female | Stage IA | Alive | | A | C |
| GSM1855164 | 54 | Female | Stage IIB | Alive | | A | A |
| GSM1855165 | 68 | Female | Stage IB | Dead | | C | B |
| GSM1855166 | 68 | Male | Stage IIB | Alive | | B | A |
| GSM1855167 | 70 | Female | Stage IIIB | Alive | | A | B |
| GSM1855168 | 77 | Female | Stage IA | Dead | | B | B |
| GSM1855169 | 68 | Female | Stage IA | Alive | | A | C |
| GSM1855170 | 67 | Male | Stage IA | Alive | | A | B |
| GSM1855171 | 76 | Male | Stage IA | Alive | | C | A |
| GSM1855172 | 81 | Male | Stage IA | Alive | | C | C |
| GSM1855173 | 77 | Male | Stage IIB | Dead | | A | B |
| GSM1855174 | 80 | Female | Stage IIIA | Alive | | A | C |
| GSM1855175 | 76 | Female | Stage IIIA | Dead | | A | C |
| GSM1855177 | 78 | Female | Stage IA | Alive | | A | C |
| GSM1855178 | 76 | Female | Stage IIB | Dead | | B | B |
| GSM1855179 | 66 | Male | Stage IB | Alive | | B | B |
| GSM1855180 | 52 | Female | Stage IIIA | Alive | | A | A |
| GSM1855181 | 62 | Male | Stage IIA | Alive | | A | B |
| GSM1855182 | 71 | Female | Stage IIB | Alive | | A | C |
| GSM1855183 | 53 | Female | Stage IIB | Alive | | A | C |
| GSM1855184 | 73 | Female | Stage IIA | Dead | | A | A |
| GSM1855185 | 67 | Male | Stage IB | Alive | | A | A |
| GSM1855186 | 57 | Female | Stage IA | Dead | | A | A |
| GSM1855187 | 76 | Male | Stage IV | Dead | | C | C |
| GSM1855188 | 82 | Male | Stage IB | Dead | | A | C |
| GSM1855189 | 76 | Male | Stage IV | Dead | | B | B |
| GSM1855190 | 49 | Female | Stage IA | Dead | | B | A |
| GSM1855191 | 70 | Male | Stage IA | Dead | | B | A |
| GSM1855192 | 70 | Female | Stage IIB | Dead | | C | C |
| GSM1855193 | 75 | Female | Stage IB | Alive | | A | C |
| GSM1855195 | 63 | Male | Stage IIB | Dead | | C | C |
| GSM1855196 | 66 | Female | Stage IIB | Dead | | B | C |
| GSM1855197 | 70 | Male | Stage IIIB | Dead | | A | C |
| GSM1855199 | 61 | Female | Stage IB | Dead | | B | B |
| GSM1855200 | 60 | Male | Stage IA | Dead | | C | C |
| GSM1855202 | 56 | Female | Stage IB | Dead | | A | B |
| GSM1855203 | 65 | Male | Stage IIIB | Alive | | A | A |
| GSM1855204 | 62 | Female | Stage IIIA | Dead | | B | C |
| GSM1855205 | 72 | Female | Stage IIIA | Alive | | A | C |
| GSM1855206 | 77 | Male | Stage IIIA | Dead | | A | A |
| GSM1855207 | 81 | Female | Stage IIB | Alive | | B | A |
| GSM1855208 | 65 | Male | Stage IB | Dead | | A | C |
| GSM1855209 | 61 | Female | Stage IB | Dead | | B | C |
| GSM1855210 | 50 | Female | Stage IA | Alive | | C | C |
| GSM1855211 | 63 | Female | Stage IB | Alive | | C | B |
| GSM1855212 | 75 | Female | Stage IV | Alive | | C | C |
| GSM1855213 | 89 | Female | Stage IA | Alive | | A | A |
| GSM1855214 | 82 | Female | Stage IA | Alive | | C | C |
| GSM1855215 | 79 | Male | Stage IB | Alive | | A | C |
| GSM1855216 | 66 | Male | Stage III | Alive | | A | C |
| GSM1855217 | 85 | Female | Stage IB | Alive | | A | C |
| GSM1855218 | 67 | Female | Stage IV | Dead | | C | C |
| GSM1855219 | 46 | Female | Stage IIIB | Dead | | C | A |
| GSM1855220 | 57 | Female | Stage IB | Alive | | A | A |
| GSM1855221 | 72 | Female | Stage IA | Alive | | A | C |
| GSM1855222 | 68 | Female | Stage IA | Alive | | A | C |
| GSM1855223 | 85 | Female | Stage IA | Dead | | A | C |
| GSM1855224 | 54 | Female | Stage IIB | Dead | | A | A |
| GSM1855225 | 72 | Male | Stage I | Dead | | A | C |
| GSM1855226 | 56 | Male | Stage IA | Alive | | B | B |
| GSM1855228 | 69 | Male | Stage IIIB | Dead | | A | B |
| GSM1855229 | 78 | Male | Stage IA | Alive | | C | C |
| GSM1855230 | 76 | Female | Stage IIB | Alive | | C | C |
| GSM1855231 | 59 | Male | Stage IIB | Dead | | A | B |
| GSM1855232 | 79 | Male | Stage IA | Dead | | B | A |
| GSM1855233 | 62 | Male | Stage IB | Dead | | A | A |
| GSM1855234 | 83 | Male | Stage IB | Alive | | C | A |
| GSM1855235 | 75 | Female | Stage IA | Dead | | A | A |
| GSM1855236 | 75 | Male | Stage IB | Alive | | C | C |
| GSM1855237 | 81 | Female | Stage IIIA | Dead | | B | B |
| GSM1855238 | 73 | Female | Stage IA | Dead | | C | B |
| TCGA-05-4244 | 70 | Male | Stage IV | Alive | | A | C |
| TCGA-05-4249 | 67 | Male | Stage IB | Alive | | A | C |
| TCGA-05-4250 | 79 | Female | Stage IIIA | Dead | | B | A |
| TCGA-05-4382 | 68 | Male | Stage IB | Alive | | A | A |
| TCGA-05-4384 | 66 | Male | Stage IIIA | Alive | | C | B |
| TCGA-05-4389 | 70 | Male | Stage IA | Alive | | B | B |
| TCGA-05-4390 | 58 | Female | Stage IB | Alive | | C | B |
| TCGA-05-4395 | 76 | Male | Stage IIIB | Dead | | B | A |
| TCGA-05-4396 | 76 | Male | Stage IIIB | Dead | | C | B |
| TCGA-05-4397 | 65 | Male | Stage IIB | Dead | | B | C |
| TCGA-05-4398 | 47 | Female | Stage IIIB | Alive | | B | A |
| TCGA-05-4402 | 57 | Female | Stage IV | Dead | | C | A |
| TCGA-05-4403 | 76 | Male | Stage IB | Alive | | B | A |
| TCGA-05-4405 | 74 | Female | Stage IB | Alive | | A | C |
| TCGA-05-4410 | 62 | Male | Stage IB | Alive | | A | C |
| TCGA-05-4415 | 57 | Male | Stage IIIB | Dead | | B | B |
| TCGA-05-4417 | 51 | Female | Stage IB | Alive | | C | B |
| TCGA-05-4418 | 69 | Male | Stage IIIA | Dead | | B | B |
| TCGA-05-4420 | 41 | Male | Stage IB | Alive | | A | C |
| TCGA-05-4422 | 68 | Male | Stage IB | Alive | | C | B |
| TCGA-05-4424 | 70 | Male | Stage IIB | Alive | | A | C |
| TCGA-05-4425 | 70 | Female | Stage IV | Alive | | A | A |
| TCGA-05-4426 | 71 | Male | Stage IB | Alive | | C | A |
| TCGA-05-4427 | 65 | Female | Stage IIB | Alive | | A | C |
| TCGA-05-4430 | 59 | Female | Stage IB | Alive | | A | A |
| TCGA-05-4432 | 66 | Male | Stage IIB | Alive | | C | C |
| TCGA-05-4433 | 82 | Male | Stage IB | Alive | | B | A |
| TCGA-05-4434 | 67 | Female | Stage IV | Dead | | B | A |
| TCGA-05-5420 | 67 | Male | Stage IIIA | Alive | | B | B |
| TCGA-05-5423 | 65 | Male | Stage IIB | Alive | | A | C |
| TCGA-05-5425 | 68 | Male | Stage IIB | Alive | | A | B |
| TCGA-05-5428 | 57 | Male | Stage IIA | Alive | | A | A |
| TCGA-05-5429 | 60 | Male | Stage IIIA | Dead | | B | A |
| TCGA-05-5715 | 69 | Female | Stage IB | Alive | | A | A |
| TCGA-35-3615 | 57 | Male | Stage IB | Alive | | C | C |
| TCGA-35-4122 | 69 | Male | Stage IA | Alive | | A | A |
| TCGA-35-4123 | 38 | Male | Stage IA | Alive | | A | A |
| TCGA-35-5375 | 61 | Male | Stage IIIA | Alive | | C | C |
| TCGA-38-4625 | 66 | Female | Stage IB | Alive | | A | A |
| TCGA-38-4627 | 64 | Female | Stage IIA | Dead | | C | A |
| TCGA-38-4628 | 65 | Female | Stage IIB | Dead | | A | C |
| TCGA-38-4629 | 68 | Male | Stage IIB | Dead | | C | A |
| TCGA-38-4630 | 75 | Female | Stage IB | Dead | | C | C |
| TCGA-38-4631 | 72 | Female | Stage IB | Dead | | C | A |
| TCGA-38-4632 | 42 | Male | Stage IV | Dead | | B | A |
| TCGA-38-6178 | 70 | Female | Stage IIIA | Alive | | C | A |
| TCGA-38-7271 | 72 | Female | Stage IA | Dead | | A | C |
| TCGA-38-A44F | 80 | Male | Stage IB | Alive | | B | A |
| TCGA-44-2655 | 65 | Female | Stage IA | Alive | | C | C |
| TCGA-44-2656 | 59 | Male | Stage IB | Alive | | A | C |
| TCGA-44-2657 | 74 | Female | Stage IB | Alive | | C | C |
| TCGA-44-2659 | 65 | Female | Stage IIB | Alive | | A | C |
| TCGA-44-2661 | 69 | Female | Stage IA | Alive | | C | C |
| TCGA-44-2662 | 65 | Male | Stage IB | Alive | | B | A |
| TCGA-44-2665 | 55 | Female | Stage IIB | Alive | | C | A |
| TCGA-44-2666 | 43 | Male | Stage IB | Dead | | C | C |
| TCGA-44-2668 | 51 | Male | Stage IB | Dead | | A | A |
| TCGA-44-3396 | 74 | Female | Stage IIIA | Alive | | A | A |
| TCGA-44-3398 | 77 | Female | Stage IA | Alive | | B | A |
| TCGA-44-3917 | 33 | Female | Stage IB | Alive | | A | C |
| TCGA-44-3918 | 60 | Female | Stage IA | Alive | | A | C |
| TCGA-44-3919 | 71 | Female | Stage IA | Dead | | C | C |
| TCGA-44-4112 | 60 | Female | Stage IB | Dead | | A | C |
| TCGA-44-5643 | 53 | Male | Stage IIIA | Alive | | C | A |
| TCGA-44-5644 | 51 | Female | Stage IB | Alive | | B | B |
| TCGA-44-5645 | 61 | Female | Stage IA | Alive | | C | C |
| TCGA-44-6145 | 62 | Female | Stage IA | Alive | | B | A |
| TCGA-44-6146 | 64 | Male | Stage IIB | Alive | | A | A |
| TCGA-44-6147 | 67 | Female | Stage IA | Alive | | A | C |
| TCGA-44-6148 | 60 | Male | Stage IA | Alive | | C | C |
| TCGA-44-6774 | 56 | Female | Stage IIIA | Alive | | C | C |
| TCGA-44-6775 | 72 | Female | Stage IB | Alive | | C | A |
| TCGA-44-6776 | 60 | Female | Stage IA | Alive | | C | C |
| TCGA-44-6777 | 85 | Female | Stage IB | Dead | | C | C |
| TCGA-44-6778 | 59 | Male | Stage IA | Alive | | C | C |
| TCGA-44-6779 | 50 | Female | Stage IIB | Dead | | A | A |
| TCGA-44-7659 | 70 | Male | Stage IA | Alive | | A | C |
| TCGA-44-7660 | 72 | Male | Stage IB | Alive | | C | A |
| TCGA-44-7661 | 69 | Female | Stage IB | Dead | | B | A |
| TCGA-44-7662 | 61 | Male | Stage IB | Alive | | B | A |
| TCGA-44-7667 | 49 | Female | Stage IIB | Alive | | C | C |
| TCGA-44-7669 | 59 | Male | Stage IIA | Dead | | C | B |
| TCGA-44-7670 | 47 | Female | Stage IIA | Alive | | B | A |
| TCGA-44-7671 | 64 | Male | Stage IB | Alive | | C | B |
| TCGA-44-7672 | 52 | Female | Stage IA | Alive | | B | C |
| TCGA-44-8117 | 54 | Female | Stage IB | Alive | | A | C |
| TCGA-44-8119 | 73 | Male | Stage IIB | Alive | | C | B |
| TCGA-44-8120 | 58 | Male | Stage IB | Alive | | A | A |
| TCGA-44-A479 | 73 | Female | Stage IB | Alive | | C | C |
| TCGA-44-A47A | 78 | Female | Stage IB | Alive | | A | C |
| TCGA-44-A47B | 79 | Male | Stage IB | Alive | | A | C |
| TCGA-44-A47G | 73 | Female | Stage IA | Alive | | B | A |
| TCGA-44-A4SS | 73 | Male | Stage IA | Alive | | A | A |
| TCGA-44-A4SU | 67 | Female | Stage IA | Dead | | C | C |
| TCGA-49-4486 | 72 | Male | Stage IA | Dead | | C | C |
| TCGA-49-4487 | 72 | Female | Stage IA | Dead | | A | B |
| TCGA-49-4488 | 74 | Female | Stage IA | Dead | | B | C |
| TCGA-49-4490 | 45 | Female | Stage IIIA | Dead | | C | A |
| TCGA-49-4494 | 77 | Male | Stage IIIA | Dead | | C | A |
| TCGA-49-4501 | 67 | Female | Stage IB | Dead | | A | C |
| TCGA-49-4505 | 61 | Female | Stage IIB | Dead | | A | C |
| TCGA-49-4506 | 68 | Female | Stage IIB | Dead | | B | B |
| TCGA-49-4507 | 73 | Female | Stage IIIA | Dead | | A | B |
| TCGA-49-4510 | 51 | Female | Stage IIB | Dead | | C | B |
| TCGA-49-4512 | 69 | Female | Stage IIIA | Dead | | A | C |
| TCGA-49-4514 | 79 | Female | Stage IA | Alive | | A | B |
| TCGA-49-6742 | 70 | Male | Stage IIA | Dead | | B | B |
| TCGA-49-6743 | 81 | Female | Stage IIIA | Alive | | B | A |
| TCGA-49-6744 | 64 | Female | Stage IIA | Alive | | A | A |
| TCGA-49-6745 | 82 | Male | Stage IIIA | Alive | | A | A |
| TCGA-49-6761 | 68 | Female | Stage IIIA | Alive | | C | A |
| TCGA-49-6767 | 46 | Female | Stage IIB | Alive | | A | A |
| TCGA-49-AAQV | 63 | Female | Stage II | Dead | | C | C |
| TCGA-49-AAR0 | 57 | Male | Stage IA | Alive | | C | C |
| TCGA-49-AAR2 | 64 | Male | Stage IB | Alive | | C | B |
| TCGA-49-AAR3 | 69 | Male | Stage IIB | Alive | | A | A |
| TCGA-49-AAR4 | 51 | Male | Stage IIIA | Dead | | C | A |
| TCGA-49-AAR9 | 61 | Male | Stage IIB | Dead | | A | A |
| TCGA-49-AARE | 51 | Female | Stage IA | Dead | | A | A |
| TCGA-49-AARN | 56 | Female | Stage IA | Dead | | B | A |
| TCGA-49-AARO | 39 | Female | Stage IA | Alive | | A | A |
| TCGA-49-AARQ | 41 | Female | Stage I | Alive | | C | C |
| TCGA-49-AARR | 68 | Male | Stage IA | Alive | | C | C |
| TCGA-4B-A93V | 52 | Female | Stage IA | Dead | | C | B |
| TCGA-50-5044 | 72 | Female | Stage IIIB | Dead | | A | A |
| TCGA-50-5049 | 70 | Male | Stage IA | Alive | | A | C |
| TCGA-50-5051 | 42 | Female | Stage IIIA | Dead | | C | B |
| TCGA-50-5055 | 79 | Female | Stage IIA | Dead | | A | C |
| TCGA-50-5066 | 72 | Male | Stage IB | Alive | | B | A |
| TCGA-50-5068 | 59 | Female | Stage IIB | Dead | | A | C |
| TCGA-50-5072 | 74 | Male | Stage IIIA | Dead | | B | B |
| TCGA-50-5930 | 47 | Male | Stage IIIA | Dead | | B | B |
| TCGA-50-5931 | 75 | Female | Stage IB | Dead | | C | A |
| TCGA-50-5932 | 75 | Male | Stage IIB | Dead | | A | C |
| TCGA-50-5933 | 72 | Male | Stage IIIB | Dead | | A | A |
| TCGA-50-5935 | 86 | Female | Stage IA | Dead | | A | C |
| TCGA-50-5936 | 58 | Male | Stage IIIA | Dead | | B | B |
| TCGA-50-5939 | 85 | Male | Stage IB | Dead | | A | A |
| TCGA-50-5941 | 55 | Female | Stage IIIA | Alive | | A | A |
| TCGA-50-5942 | 67 | Female | Stage IA | Alive | | A | C |
| TCGA-50-5944 | 69 | Female | Stage IA | Alive | | C | C |
| TCGA-50-5946 | 62 | Male | Stage IA | Alive | | A | C |
| TCGA-50-6590 | 72 | Female | Stage IB | Dead | | A | A |
| TCGA-50-6591 | 63 | Female | Stage IV | Dead | | C | C |
| TCGA-50-6592 | 71 | Female | Stage IB | Dead | | B | A |
| TCGA-50-6593 | 49 | Female | Stage IIIA | Dead | | A | A |
| TCGA-50-6594 | 79 | Female | Stage IIIA | Dead | | A | C |
| TCGA-50-6595 | 74 | Female | Stage IIIA | Dead | | B | A |
| TCGA-50-6597 | 79 | Female | Stage IB | Dead | | A | C |
| TCGA-50-6673 | 84 | Female | Stage I | Dead | | B | A |
| TCGA-50-7109 | 60 | Male | Stage IA | Dead | | C | B |
| TCGA-50-8457 | 63 | Female | Stage IA | Alive | | A | C |
| TCGA-50-8459 | 68 | Male | Stage IIB | Alive | | B | A |
| TCGA-50-8460 | 74 | Male | Stage IA | Alive | | C | C |
| TCGA-53-7624 | 40 | Female | Stage IV | Dead | | B | A |
| TCGA-53-7626 | 76 | Female | Stage IIA | Dead | | A | C |
| TCGA-53-7813 | 51 | Female | Stage IIIB | Alive | | C | C |
| TCGA-53-A4EZ | 63 | Male | Stage IIA | Alive | | C | C |
| TCGA-55-1592 | 65 | Male | Stage IA | Dead | | C | C |
| TCGA-55-1594 | 68 | Male | Stage IIIA | Alive | | C | C |
| TCGA-55-1596 | 55 | Male | Stage IIB | Alive | | A | A |
| TCGA-55-6543 | 60 | Female | Stage IA | Alive | | C | C |
| TCGA-55-6642 | 63 | Male | Stage IB | Alive | | A | B |
| TCGA-55-6712 | 71 | Male | Stage IIA | Dead | | A | A |
| TCGA-55-6968 | 61 | Male | Stage IV | Dead | | C | C |
| TCGA-55-6970 | 67 | Female | Stage IIIA | Dead | | B | B |
| TCGA-55-6971 | 59 | Female | Stage IB | Alive | | C | C |
| TCGA-55-6972 | 72 | Male | Stage IB | Dead | | A | C |
| TCGA-55-6975 | 61 | Male | Stage IIB | Dead | | C | B |
| TCGA-55-6978 | 81 | Male | Stage IIA | Dead | | A | A |
| TCGA-55-6979 | 59 | Female | Stage IIB | Dead | | B | A |
| TCGA-55-6980 | 56 | Male | Stage IA | Alive | | C | A |
| TCGA-55-6981 | 53 | Female | Stage IIIA | Dead | | C | A |
| TCGA-55-6982 | 79 | Female | Stage IIB | Dead | | C | A |
| TCGA-55-6983 | 81 | Male | Stage IIB | Alive | | C | C |
| TCGA-55-6984 | 71 | Female | Stage IIB | Dead | | B | A |
| TCGA-55-6985 | 58 | Female | Stage IB | Alive | | C | A |
| TCGA-55-6986 | 74 | Female | Stage IB | Alive | | C | C |
| TCGA-55-6987 | 77 | Male | Stage IA | Alive | | A | B |
| TCGA-55-7227 | 77 | Male | Stage IIIA | Dead | | A | C |
| TCGA-55-7281 | 70 | Female | Stage IA | Alive | | A | A |
| TCGA-55-7283 | 76 | Female | Stage IIIA | Alive | | A | C |
| TCGA-55-7284 | 74 | Male | Stage IIB | Dead | | A | A |
| TCGA-55-7570 | 60 | Male | Stage IA | Alive | | C | A |
| TCGA-55-7573 | 72 | Female | Stage IA | Alive | | A | C |
| TCGA-55-7574 | 64 | Female | Stage IB | Dead | | A | C |
| TCGA-55-7576 | 54 | Male | Stage IB | Alive | | A | A |
| TCGA-55-7724 | 76 | Female | Stage IB | Alive | | A | A |
| TCGA-55-7725 | 68 | Female | Stage IA | Alive | | A | C |
| TCGA-55-7726 | 72 | Female | Stage IA | Alive | | A | A |
| TCGA-55-7727 | 70 | Male | Stage IIIA | Alive | | A | C |
| TCGA-55-7728 | 64 | Female | Stage IB | Alive | | A | A |
| TCGA-55-7815 | 76 | Male | Stage IB | Alive | | B | C |
| TCGA-55-7816 | 49 | Female | Stage IV | Dead | | B | C |
| TCGA-55-7903 | 64 | Male | Stage IA | Alive | | A | C |
| TCGA-55-7907 | 77 | Male | Stage IIA | Dead | | A | A |
| TCGA-55-7910 | 50 | Female | Stage IIA | Alive | | A | B |
| TCGA-55-7911 | 70 | Female | Stage IA | Alive | | A | C |
| TCGA-55-7913 | 61 | Female | Stage IA | Dead | | A | B |
| TCGA-55-7914 | 71 | Female | Stage IIA | Dead | | A | C |
| TCGA-55-7994 | 81 | Male | Stage IIB | Alive | | A | A |
| TCGA-55-7995 | 73 | Female | Stage IA | Alive | | C | C |
| TCGA-55-8085 | 64 | Male | Stage IA | Alive | | C | C |
| TCGA-55-8087 | 59 | Female | Stage IB | Alive | | A | C |
| TCGA-55-8089 | 56 | Male | Stage IA | Dead | | B | A |
| TCGA-55-8090 | 80 | Male | Stage IA | Dead | | C | C |
| TCGA-55-8091 | 74 | Male | Stage IB | Alive | | C | C |
| TCGA-55-8092 | 75 | Male | Stage IIB | Dead | | A | B |
| TCGA-55-8094 | 51 | Male | Stage IV | Alive | | B | B |
| TCGA-55-8096 | 67 | Female | Stage IB | Dead | | C | C |
| TCGA-55-8097 | 60 | Female | Stage IA | Alive | | A | C |
| TCGA-55-8203 | 69 | Female | Stage IA | Alive | | A | C |
| TCGA-55-8204 | 87 | Female | Stage IB | Alive | | C | A |
| TCGA-55-8205 | 76 | Female | Stage IIA | Alive | | B | A |
| TCGA-55-8206 | 56 | Male | Stage IA | Alive | | A | C |
| TCGA-55-8207 | 73 | Male | Stage IB | Alive | | A | C |
| TCGA-55-8208 | 73 | Female | Stage IA | Alive | | B | A |
| TCGA-55-8299 | 61 | Female | Stage IA | Dead | | B | B |
| TCGA-55-8301 | 58 | Male | Stage IB | Alive | | A | C |
| TCGA-55-8302 | 54 | Male | Stage IB | Alive | | A | A |
| TCGA-55-8505 | 62 | Male | Stage IIIA | Alive | | B | A |
| TCGA-55-8506 | 62 | Female | Stage IIB | Alive | | A | C |
| TCGA-55-8507 | 53 | Male | Stage IA | Alive | | C | C |
| TCGA-55-8508 | 60 | Female | Stage IIA | Alive | | C | B |
| TCGA-55-8510 | 55 | Female | Stage IB | Alive | | A | A |
| TCGA-55-8511 | 73 | Female | Stage IB | Alive | | A | A |
| TCGA-55-8512 | 41 | Male | Stage IV | Dead | | C | A |
| TCGA-55-8513 | 77 | Female | Stage IIB | Alive | | A | A |
| TCGA-55-8514 | 70 | Female | Stage IB | Alive | | C | C |
| TCGA-55-8614 | 76 | Male | Stage IB | Alive | | C | A |
| TCGA-55-8615 | 67 | Male | Stage IIIA | Alive | | C | B |
| TCGA-55-8616 | 58 | Female | Stage IB | Alive | | A | A |
| TCGA-55-8619 | 72 | Female | Stage IIB | Alive | | B | A |
| TCGA-55-8620 | 60 | Male | Stage IV | Dead | | C | C |
| TCGA-55-8621 | 75 | Female | Stage IA | Alive | | C | C |
| TCGA-55-A48X | 63 | Female | Stage IIA | Alive | | A | C |
| TCGA-55-A48Y | 69 | Male | Stage IIA | Alive | | A | B |
| TCGA-55-A48Z | 60 | Female | Stage IIIB | Alive | | C | C |
| TCGA-55-A490 | 78 | Male | Stage IIA | Dead | | A | C |
| TCGA-55-A491 | 81 | Female | Stage IA | Alive | | A | C |
| TCGA-55-A492 | 70 | Female | Stage IA | Alive | | C | B |
| TCGA-55-A493 | 54 | Female | Stage IB | Alive | | B | A |
| TCGA-55-A494 | 61 | Female | Stage IB | Alive | | A | C |
| TCGA-55-A4DF | 88 | Male | Stage IA | Dead | | C | C |
| TCGA-55-A4DG | 71 | Male | Stage IA | Alive | | A | C |
| TCGA-55-A57B | 80 | Female | Stage IA | Alive | | C | C |
| TCGA-62-8394 | 65 | Female | Stage IIIB | Dead | | C | A |
| TCGA-62-8395 | 80 | Female | Stage IIB | Alive | | A | C |
| TCGA-62-8397 | 70 | Female | Stage IIB | Alive | | A | C |
| TCGA-62-8398 | 55 | Male | Stage IIIA | Dead | | B | B |
| TCGA-62-8399 | 62 | Male | Stage IIIA | Alive | | B | A |
| TCGA-62-8402 | 73 | Female | Stage IIIA | Dead | | C | C |
| TCGA-62-A46O | 65 | Female | Stage IB | Dead | | A | B |
| TCGA-62-A46P | 65 | Male | Stage IB | Dead | | A | C |
| TCGA-62-A46R | 54 | Female | Stage IB | Dead | | A | C |
| TCGA-62-A46S | 73 | Male | Stage IB | Dead | | C | B |
| TCGA-62-A46V | 78 | Female | Stage IB | Alive | | A | C |
| TCGA-62-A46Y | 70 | Female | Stage IIIA | Dead | | C | C |
| TCGA-62-A470 | 84 | Male | Stage IB | Dead | | C | B |
| TCGA-62-A471 | 64 | Male | Stage IIB | Alive | | B | B |
| TCGA-62-A472 | 70 | Male | Stage IIB | Alive | | A | C |
| TCGA-64-1676 | 58 | Male | Stage IA | Alive | | A | C |
| TCGA-64-1677 | 77 | Female | Stage IIIA | Dead | | C | C |
| TCGA-64-1679 | 58 | Female | Stage IIIA | Alive | | C | A |
| TCGA-64-1680 | 63 | Male | Stage IV | Alive | | C | C |
| TCGA-64-1681 | 61 | Female | Stage IA | Dead | | C | C |
| TCGA-64-5774 | 60 | Male | Stage IB | Alive | | C | B |
| TCGA-64-5775 | 71 | Male | Stage IIIA | Dead | | A | A |
| TCGA-64-5778 | 60 | Male | Stage IB | Alive | | A | C |
| TCGA-64-5779 | 61 | Male | Stage IIIA | Alive | | C | C |
| TCGA-64-5781 | 55 | Female | Stage IB | Alive | | A | A |
| TCGA-64-5815 | 74 | Male | Stage IIB | Alive | | B | A |
| TCGA-67-3770 | 70 | Female | Stage IA | Alive | | C | C |
| TCGA-67-3771 | 77 | Female | Stage IA | Alive | | C | A |
| TCGA-67-3772 | 82 | Female | Stage IB | Alive | | C | C |
| TCGA-67-3773 | 84 | Female | Stage IB | Alive | | A | C |
| TCGA-67-3774 | 73 | Female | Stage IB | Alive | | C | B |
| TCGA-67-6215 | 52 | Female | Stage IB | Alive | | A | C |
| TCGA-67-6216 | 57 | Female | Stage IA | Alive | | C | C |
| TCGA-67-6217 | 73 | Female | Stage IIA | Alive | | A | C |
| TCGA-69-7760 | 73 | Male | Stage IIB | Alive | | A | C |
| TCGA-69-7761 | 84 | Male | Stage IB | Alive | | B | A |
| TCGA-69-7763 | 69 | Male | Stage IA | Alive | | A | A |
| TCGA-69-7764 | 75 | Male | Stage IA | Alive | | C | B |
| TCGA-69-7973 | 42 | Female | Stage IB | Alive | | C | B |
| TCGA-69-7974 | 54 | Female | Stage IIIA | Alive | | A | A |
| TCGA-69-7978 | 59 | Male | Stage IIB | Alive | | B | A |
| TCGA-69-7979 | 71 | Female | Stage IB | Alive | | A | A |
| TCGA-69-7980 | 70 | Female | Stage I | Alive | | A | A |
| TCGA-69-8253 | 59 | Female | Stage IIA | Alive | | C | A |
| TCGA-69-8255 | 71 | Male | Stage IA | Alive | | B | B |
| TCGA-69-8453 | 77 | Male | Stage IIB | Alive | | B | A |
| TCGA-69-A59K | 60 | Female | Stage IIB | Alive | | A | C |
| TCGA-71-6725 | 48 | Female | Stage IB | Alive | | A | C |
| TCGA-71-8520 | 60 | Female | Stage IB | Dead | | C | C |
| TCGA-73-4658 | 80 | Female | Stage IB | Dead | | A | A |
| TCGA-73-4659 | 66 | Male | Stage IIIA | Dead | | A | B |
| TCGA-73-4662 | 65 | Female | Stage IA | Alive | | A | C |
| TCGA-73-4666 | 52 | Female | Stage IV | Alive | | A | A |
| TCGA-73-4668 | 66 | Female | Stage IIB | Alive | | B | A |
| TCGA-73-4670 | 69 | Female | Stage IV | Alive | | B | B |
| TCGA-73-4675 | 59 | Male | Stage IIIA | Dead | | C | A |
| TCGA-73-4676 | 45 | Male | Stage IIA | Dead | | B | A |
| TCGA-73-7498 | 58 | Female | Stage IA | Alive | | C | C |
| TCGA-73-7499 | 81 | Female | Stage IB | Dead | | A | C |
| TCGA-73-A9RS | 41 | Male | Stage IIB | Dead | | A | B |
| TCGA-78-7143 | 62 | Female | Stage IB | Dead | | C | C |
| TCGA-78-7145 | 52 | Female | Stage IV | Dead | | C | A |
| TCGA-78-7146 | 71 | Female | Stage IIIA | Dead | | A | A |
| TCGA-78-7147 | 67 | Female | Stage IIB | Dead | | C | C |
| TCGA-78-7148 | 71 | Male | Stage IIB | Dead | | C | B |
| TCGA-78-7149 | 71 | Male | Stage IIIB | Alive | | C | C |
| TCGA-78-7150 | 59 | Male | Stage IIB | Dead | | B | B |
| TCGA-78-7152 | 65 | Male | Stage IB | Dead | | C | C |
| TCGA-78-7153 | 65 | Female | Stage IB | Alive | | C | C |
| TCGA-78-7154 | 72 | Male | Stage IIIA | Dead | | B | A |
| TCGA-78-7155 | 68 | Male | Stage IB | Dead | | C | C |
| TCGA-78-7156 | 62 | Male | Stage IV | Dead | | C | B |
| TCGA-78-7158 | 59 | Female | Stage IIIB | Dead | | A | C |
| TCGA-78-7159 | 60 | Female | Stage IA | Alive | | C | B |
| TCGA-78-7160 | 61 | Male | Stage IV | Dead | | A | B |
| TCGA-78-7161 | 69 | Female | Stage IIB | Dead | | C | B |
| TCGA-78-7162 | 75 | Male | Stage IA | Dead | | C | B |
| TCGA-78-7163 | 60 | Male | Stage IB | Alive | | C | C |
| TCGA-78-7166 | 84 | Male | Stage IIB | Dead | | A | B |
| TCGA-78-7167 | 77 | Male | Stage IV | Dead | | C | B |
| TCGA-78-7220 | 53 | Female | Stage IIIA | Dead | | B | B |
| TCGA-78-7535 | 45 | Male | Stage IB | Dead | | A | A |
| TCGA-78-7536 | 69 | Male | Stage IIIA | Dead | | A | B |
| TCGA-78-7537 | 72 | Male | Stage IB | Dead | | C | C |
| TCGA-78-7539 | 75 | Female | Stage IIA | Alive | | A | C |
| TCGA-78-7540 | 66 | Female | Stage IB | Dead | | A | A |
| TCGA-78-7542 | 56 | Male | Stage IB | Dead | | A | A |
| TCGA-78-7633 | 67 | Male | Stage IB | Dead | | C | B |
| TCGA-78-8640 | 59 | Male | Stage IIA | Alive | | A | C |
| TCGA-78-8648 | 58 | Female | Stage IIB | Dead | | A | C |
| TCGA-78-8655 | 77 | Female | Stage IA | Alive | | A | C |
| TCGA-78-8660 | 69 | Male | Stage IIB | Dead | | B | A |
| TCGA-78-8662 | 53 | Female | Stage IB | Dead | | A | C |
| TCGA-83-5908 | 59 | Female | Stage IA | Alive | | B | A |
| TCGA-86-6562 | 52 | Male | Stage IIA | Dead | | A | A |
| TCGA-86-6851 | 73 | Female | Stage IIA | Alive | | A | C |
| TCGA-86-7701 | 66 | Male | Stage IV | Alive | | C | B |
| TCGA-86-7711 | 70 | Male | Stage IIA | Dead | | A | A |
| TCGA-86-7713 | 70 | Male | Stage IIA | Alive | | C | B |
| TCGA-86-7714 | 61 | Female | Stage IIIA | Dead | | A | C |
| TCGA-86-7953 | 69 | Female | Stage IA | Alive | | C | C |
| TCGA-86-7954 | 68 | Female | Stage IB | Alive | | A | C |
| TCGA-86-7955 | 62 | Male | Stage IB | Alive | | A | B |
| TCGA-86-8054 | 61 | Male | Stage IIB | Alive | | B | B |
| TCGA-86-8055 | 79 | Male | Stage IIA | Dead | | C | A |
| TCGA-86-8056 | 63 | Female | Stage IIIA | Alive | | A | C |
| TCGA-86-8073 | 58 | Male | Stage IB | Alive | | A | A |
| TCGA-86-8074 | 62 | Female | Stage IIA | Alive | | C | A |
| TCGA-86-8075 | 66 | Female | Stage IB | Dead | | C | A |
| TCGA-86-8076 | 42 | Male | Stage IA | Alive | | A | B |
| TCGA-86-8278 | 63 | Female | Stage IIB | Alive | | C | A |
| TCGA-86-8279 | 46 | Male | Stage IIA | Alive | | A | C |
| TCGA-86-8280 | 54 | Female | Stage IIA | Alive | | C | C |
| TCGA-86-8281 | 75 | Male | Stage IA | Alive | | C | C |
| TCGA-86-8358 | 44 | Male | Stage IB | Alive | | C | C |
| TCGA-86-8359 | 52 | Male | Stage IIIA | Dead | | C | B |
| TCGA-86-8585 | 57 | Male | Stage IB | Alive | | A | B |
| TCGA-86-8668 | 61 | Female | Stage IA | Alive | | C | C |
| TCGA-86-8669 | 64 | Male | Stage IA | Alive | | C | C |
| TCGA-86-8671 | 72 | Female | Stage IIB | Alive | | A | C |
| TCGA-86-8672 | 59 | Male | Stage IIB | Dead | | A | A |
| TCGA-86-8673 | 61 | Male | Stage IB | Alive | | C | A |
| TCGA-86-8674 | 50 | Male | Stage IIA | Alive | | C | A |
| TCGA-86-A456 | 78 | Female | Stage IA | Alive | | A | C |
| TCGA-86-A4D0 | 48 | Male | Stage IIA | Dead | | B | B |
| TCGA-86-A4JF | 56 | Male | Stage IIB | Dead | | A | C |
| TCGA-86-A4P7 | 63 | Female | Stage IB | Alive | | A | C |
| TCGA-86-A4P8 | 59 | Female | Stage IIIA | Alive | | A | C |
| TCGA-91-6828 | 70 | Male | Stage IA | Alive | | A | C |
| TCGA-91-6829 | 78 | Male | Stage IB | Dead | | C | C |
| TCGA-91-6830 | 65 | Female | Stage IIA | Alive | | B | A |
| TCGA-91-6831 | 66 | Male | Stage IB | Alive | | C | B |
| TCGA-91-6835 | 81 | Female | Stage IA | Alive | | A | C |
| TCGA-91-6836 | 52 | Female | Stage IB | Alive | | A | C |
| TCGA-91-6840 | 59 | Female | Stage IA | Alive | | C | C |
| TCGA-91-6847 | 62 | Female | Stage IB | Alive | | C | C |
| TCGA-91-6848 | 59 | Male | Stage IIIA | Alive | | A | A |
| TCGA-91-6849 | 75 | Female | Stage IIIA | Alive | | C | A |
| TCGA-91-7771 | 62 | Male | Stage IIB | Alive | | C | C |
| TCGA-91-8496 | 63 | Female | Stage IB | Alive | | C | C |
| TCGA-91-8497 | 75 | Female | Stage IA | Dead | | C | C |
| TCGA-91-8499 | 76 | Female | Stage IA | Alive | | C | C |
| TCGA-91-A4BC | 59 | Male | Stage IIA | Alive | | B | B |
| TCGA-91-A4BD | 78 | Male | Stage IIA | Alive | | A | C |
| TCGA-93-7347 | 76 | Female | Stage IA | Alive | | C | C |
| TCGA-93-7348 | 75 | Female | Stage IA | Alive | | C | C |
| TCGA-93-8067 | 77 | Male | Stage IB | Alive | | A | B |
| TCGA-93-A4JN | 71 | Male | Stage IV | Alive | | A | C |
| TCGA-93-A4JO | 70 | Male | Stage IA | Dead | | A | C |
| TCGA-93-A4JP | 64 | Male | Stage IV | Alive | | A | C |
| TCGA-93-A4JQ | 49 | Male | Stage IA | Alive | | A | A |
| TCGA-95-7039 | 54 | Female | Stage IIB | Alive | | A | A |
| TCGA-95-7043 | 63 | Female | Stage IA | Dead | | A | C |
| TCGA-95-7562 | 71 | Male | Stage IIA | Dead | | C | C |
| TCGA-95-7567 | 61 | Male | Stage IIB | Alive | | A | C |
| TCGA-95-7944 | 71 | Male | Stage IA | Alive | | A | A |
| TCGA-95-7947 | 67 | Male | Stage IA | Alive | | A | C |
| TCGA-95-7948 | 42 | Female | Stage IB | Alive | | C | C |
| TCGA-95-8039 | 72 | Male | Stage IA | Alive | | A | C |
| TCGA-95-8494 | 67 | Male | Stage IIA | Alive | | A | A |
| TCGA-95-A4VK | 74 | Female | Stage IIIA | Alive | | A | A |
| TCGA-95-A4VN | 62 | Female | Stage IIA | Alive | | A | A |
| TCGA-95-A4VP | 66 | Female | Stage IIIA | Alive | | C | B |
| TCGA-97-7546 | 76 | Female | Stage IA | Alive | | A | C |
| TCGA-97-7547 | 67 | Female | Stage IB | Alive | | C | C |
| TCGA-97-7552 | 70 | Male | Stage IB | Alive | | A | A |
| TCGA-97-7553 | 58 | Female | Stage IA | Alive | | A | C |
| TCGA-97-7554 | 83 | Female | Stage IIIA | Alive | | A | C |
| TCGA-97-7937 | 65 | Male | Stage IB | Alive | | A | C |
| TCGA-97-7938 | 76 | Female | Stage IA | Dead | | A | C |
| TCGA-97-7941 | 72 | Female | Stage IA | Alive | | A | A |
| TCGA-97-8171 | 81 | Male | Stage IV | Alive | | A | C |
| TCGA-97-8172 | 75 | Female | Stage IB | Alive | | A | C |
| TCGA-97-8174 | 67 | Male | Stage IIA | Dead | | A | B |
| TCGA-97-8175 | 55 | Female | Stage IB | Alive | | B | A |
| TCGA-97-8176 | 63 | Male | Stage IIIA | Dead | | B | B |
| TCGA-97-8177 | 59 | Female | Stage IB | Alive | | C | C |
| TCGA-97-8179 | 72 | Male | Stage IA | Alive | | A | B |
| TCGA-97-8547 | 78 | Female | Stage IIIA | Alive | | A | A |
| TCGA-97-8552 | 55 | Female | Stage I | Alive | | C | C |
| TCGA-97-A4LX | 81 | Male | Stage IB | Alive | | A | C |
| TCGA-97-A4M0 | 60 | Female | Stage IB | Alive | | A | C |
| TCGA-97-A4M1 | 52 | Female | Stage IA | Alive | | A | C |
| TCGA-97-A4M2 | 66 | Male | Stage IA | Alive | | A | C |
| TCGA-97-A4M3 | 69 | Female | Stage IA | Alive | | B | B |
| TCGA-97-A4M5 | 83 | Male | Stage IA | Alive | | C | B |
| TCGA-97-A4M6 | 45 | Female | Stage IA | Alive | | A | C |
| TCGA-97-A4M7 | 74 | Male | Stage IA | Alive | | C | C |
| TCGA-99-7458 | 74 | Female | Stage IIIA | Alive | | A | C |
| TCGA-99-8025 | 72 | Female | Stage IIIA | Alive | | A | A |
| TCGA-99-8028 | 50 | Female | Stage IA | Alive | | A | C |
| TCGA-99-8032 | 61 | Male | Stage IA | Alive | | C | B |
| TCGA-99-8033 | 74 | Female | Stage IV | Dead | | A | B |
| TCGA-99-AA5R | 70 | Female | Stage IA | Alive | | A | C |
| TCGA-J2-8192 | 65 | Female | Stage IIA | Alive | | C | A |
| TCGA-J2-8194 | 69 | Female | Stage IIB | Alive | | C | B |
| TCGA-J2-A4AD | 61 | Female | Stage IA | Dead | | A | A |
| TCGA-J2-A4AE | 77 | Female | Stage IA | Alive | | C | C |
| TCGA-J2-A4AG | 66 | Female | Stage IA | Alive | | A | A |
| TCGA-L4-A4E5 | 48 | Female | Stage I | Alive | | A | C |
| TCGA-L4-A4E6 | 67 | Male | Stage IA | Alive | | A | C |
| TCGA-L9-A443 | 63 | Female | Stage IA | Dead | | A | C |
| TCGA-L9-A444 | 60 | Female | Stage IA | Alive | | A | C |
| TCGA-L9-A50W | 75 | Male | Stage IIA | Dead | | B | C |
| TCGA-L9-A5IP | 40 | Female | Stage IV | Dead | | B | A |
| TCGA-L9-A743 | 56 | Male | Stage IIA | Alive | | B | A |
| TCGA-L9-A7SV | 69 | Male | Stage IIA | Alive | | A | C |
| TCGA-L9-A8F4 | 64 | Female | Stage IB | Alive | | A | C |
| TCGA-MN-A4N1 | 60 | Male | Stage IIA | Alive | | A | A |
| TCGA-MN-A4N4 | 57 | Male | Stage IA | Alive | | B | A |
| TCGA-MN-A4N5 | 63 | Male | Stage IA | Alive | | A | A |
| TCGA-MP-A4SV | 67 | Male | Stage IB | Dead | | A | A |
| TCGA-MP-A4SW | 53 | Male | Stage IIB | Dead | | C | C |
| TCGA-MP-A4SY | 61 | Male | Stage IIB | Dead | | B | A |
| TCGA-MP-A4T4 | 68 | Female | Stage IIB | Dead | | A | A |
| TCGA-MP-A4T6 | 76 | Female | Stage IIIA | Dead | | A | C |
| TCGA-MP-A4T7 | 75 | Female | Stage IV | Dead | | B | B |
| TCGA-MP-A4T8 | 68 | Male | Stage IIIA | Dead | | C | B |
| TCGA-MP-A4T9 | 54 | Female | Stage IIIA | Dead | | A | A |
| TCGA-MP-A4TA | 75 | Female | Stage IA | Dead | | A | B |
| TCGA-MP-A4TC | 77 | Male | Stage IIIA | Dead | | B | A |
| TCGA-MP-A4TD | 71 | Male | Stage IIIA | Dead | | C | B |
| TCGA-MP-A4TE | 56 | Male | Stage IIA | Dead | | C | B |
| TCGA-MP-A4TF | 58 | Female | Stage IIA | Dead | | A | A |
| TCGA-MP-A4TH | 70 | Female | Stage IA | Alive | | A | C |
| TCGA-MP-A4TI | 72 | Male | Stage IIA | Dead | | A | A |
| TCGA-MP-A4TJ | 62 | Female | Stage IA | Dead | | C | C |
| TCGA-MP-A4TK | 56 | Female | Stage IIB | Dead | | B | A |
| TCGA-MP-A5C7 | 76 | Female | Stage IB | Alive | | C | C |
| TCGA-NJ-A4YF | 50 | Female | Stage IA | Alive | | C | B |
| TCGA-NJ-A4YG | 65 | Male | Stage IB | Alive | | A | C |
| TCGA-NJ-A4YI | 87 | Female | Stage IIIA | Dead | | A | C |
| TCGA-NJ-A4YP | 52 | Male | Stage IB | Alive | | A | B |
| TCGA-NJ-A4YQ | 69 | Female | Stage IA | Alive | | A | A |
| TCGA-NJ-A55A | 76 | Female | Stage IB | Alive | | C | C |
| TCGA-NJ-A55O | 56 | Female | Stage IIA | Alive | | C | B |
| TCGA-NJ-A55R | 67 | Male | Stage IA | Alive | | C | C |
| TCGA-NJ-A7XG | 49 | Male | Stage IIIA | Alive | | A | C |
| TCGA-O1-A52J | 74 | Female | Stage IA | Dead | | C | C |
| TCGA-S2-AA1A | 68 | Female | Stage IA | Alive | | C | C |

**Supplementary Table S2 Functional annotation for m^6^A-related genes (Gene Ontology-Biological process)**

| Ontology | Description | GeneRatio | pvalue | qvalue |
| --- | --- | --- | --- | --- |
| BP | T cell activation | 74/738 | 2.63E-24 | 1.08E-20 |
| BP | Positive regulation of leukocyte activation | 65/738 | 1.66E-22 | 3.41E-19 |
| BP | Positive regulation of cell activation | 66/738 | 2.54E-22 | 3.47E-19 |
| BP | Positive regulation of T cell activation | 45/738 | 1.03E-20 | 8.63E-18 |
| BP | Positive regulation of leukocyte cell-cell adhesion | 47/738 | 1.26E-20 | 8.63E-18 |
| BP | Regulation of T cell activation | 56/738 | 1.26E-20 | 8.63E-18 |
| BP | Positive regulation of lymphocyte activation | 56/738 | 4.24E-19 | 2.48E-16 |
| BP | Positive regulation of cytokine production | 63/738 | 6.48E-19 | 3.32E-16 |
| BP | Positive regulation of cell-cell adhesion | 48/738 | 3.37E-18 | 1.53E-15 |
| BP | Leukocyte cell-cell adhesion | 55/738 | 5.08E-18 | 2.08E-15 |
| BP | Regulation of leukocyte cell-cell adhesion | 52/738 | 5.86E-18 | 2.18E-15 |
| BP | Regulation of leukocyte proliferation | 42/738 | 2.38E-16 | 8.11E-14 |
| BP | Regulation of mononuclear cell proliferation | 40/738 | 3.74E-16 | 1.18E-13 |
| BP | Positive regulation of cell adhesion | 57/738 | 4.42E-16 | 1.30E-13 |
| BP | Mononuclear cell proliferation | 45/738 | 1.26E-15 | 3.45E-13 |
| BP | Regulation of lymphocyte proliferation | 39/738 | 1.55E-15 | 3.97E-13 |
| BP | Leukocyte proliferation | 47/738 | 1.84E-15 | 4.44E-13 |
| BP | Lymphocyte proliferation | 44/738 | 4.12E-15 | 9.15E-13 |
| BP | Interferon-gamma production | 28/738 | 4.24E-15 | 9.15E-13 |
| BP | Regulation of immune effector process | 58/738 | 7.66E-15 | 1.57E-12 |
| BP | Alpha-beta T cell activation | 31/738 | 8.60E-15 | 1.68E-12 |
| BP | Lymphocyte differentiation | 50/738 | 1.36E-14 | 2.47E-12 |
| BP | Regulation of leukocyte mediated immunity | 37/738 | 1.38E-14 | 2.47E-12 |
| BP | Regulation of cell-cell adhesion | 55/738 | 2.07E-14 | 3.53E-12 |
| BP | T cell differentiation | 40/738 | 4.23E-14 | 6.93E-12 |
| BP | Regulation of adaptive immune response | 32/738 | 7.97E-14 | 1.26E-11 |
| BP | Regulation of interferon-gamma production | 25/738 | 1.73E-13 | 2.62E-11 |
| BP | Regulation of leukocyte differentiation | 42/738 | 2.11E-13 | 3.09E-11 |
| BP | Regulation of hemopoiesis | 57/738 | 3.35E-13 | 4.74E-11 |
| BP | Positive regulation of immune effector process | 36/738 | 4.24E-13 | 5.79E-11 |
| BP | Positive regulation of hemopoiesis | 34/738 | 7.50E-13 | 9.92E-11 |
| BP | Regulation of response to biotic stimulus | 50/738 | 8.47E-13 | 1.08E-10 |
| BP | T cell proliferation | 33/738 | 1.06E-12 | 1.31E-10 |
| BP | Negative regulation of immune system process | 53/738 | 2.33E-12 | 2.81E-10 |
| BP | Regulation of innate immune response | 41/738 | 6.03E-12 | 7.06E-10 |
| BP | Regulation of adaptive immune response based on somatic recombination of immune receptors built from immunoglobulin superfamily domains | 28/738 | 7.96E-12 | 9.06E-10 |
| BP | Adaptive immune response based on somatic recombination of immune receptors built from immunoglobulin superfamily domains | 45/738 | 1.45E-11 | 1.60E-09 |
| BP | Regulation of alpha-beta T cell activation | 22/738 | 1.71E-11 | 1.85E-09 |
| BP | Positive regulation of mononuclear cell proliferation | 26/738 | 1.89E-11 | 1.99E-09 |
| BP | Regulation of lymphocyte differentiation | 30/738 | 1.96E-11 | 2.01E-09 |
| BP | Positive regulation of leukocyte differentiation | 28/738 | 2.08E-11 | 2.08E-09 |
| BP | Positive regulation of leukocyte mediated immunity | 26/738 | 2.25E-11 | 2.20E-09 |
| BP | Positive regulation of leukocyte proliferation | 27/738 | 2.81E-11 | 2.68E-09 |
| BP | Immune response-activating cell surface receptor signaling pathway | 52/738 | 3.29E-11 | 2.96E-09 |
| BP | Immune response-activating signal transduction | 52/738 | 3.29E-11 | 2.96E-09 |
| BP | Positive regulation of interferon-gamma production | 18/738 | 3.32E-11 | 2.96E-09 |
| BP | Regulation of lymphocyte mediated immunity | 27/738 | 5.28E-11 | 4.61E-09 |
| BP | Regulation of T cell proliferation | 28/738 | 6.95E-11 | 5.94E-09 |
| BP | Positive regulation of lymphocyte proliferation | 25/738 | 9.52E-11 | 7.96E-09 |
| BP | T cell receptor signaling pathway | 31/738 | 1.15E-10 | 9.45E-09 |
| BP | Positive regulation of defense response | 44/738 | 1.90E-10 | 1.53E-08 |
| BP | Cytokine production involved in immune response | 21/738 | 4.98E-10 | 3.92E-08 |
| BP | Myeloid cell differentiation | 46/738 | 7.08E-10 | 5.47E-08 |
| BP | Alpha-beta T cell differentiation | 21/738 | 7.20E-10 | 5.47E-08 |
| BP | Regulation of T cell differentiation | 25/738 | 8.29E-10 | 6.18E-08 |
| BP | Positive regulation of lymphocyte differentiation | 20/738 | 3.76E-09 | 2.75E-07 |
| BP | Response to virus | 39/738 | 3.95E-09 | 2.84E-07 |
| BP | Lymphocyte mediated immunity | 40/738 | 4.74E-09 | 3.35E-07 |
| BP | CD4-positive, alpha-beta T cell activation | 19/738 | 5.67E-09 | 3.94E-07 |
| BP | T-helper 1 type immune response | 13/738 | 7.65E-09 | 5.22E-07 |
| BP | Positive regulation of alpha-beta T cell activation | 15/738 | 1.42E-08 | 9.31E-07 |
| BP | Regulation of CD4-positive, alpha-beta T cell activation | 15/738 | 1.42E-08 | 9.31E-07 |
| BP | Myeloid leukocyte differentiation | 28/738 | 1.45E-08 | 9.31E-07 |
| BP | Positive regulation of T cell differentiation | 18/738 | 1.45E-08 | 9.31E-07 |
| BP | Positive regulation of cytokine production involved in immune response | 14/738 | 1.71E-08 | 1.08E-06 |
| BP | Regulation of cytokine production involved in immune response | 17/738 | 1.78E-08 | 1.09E-06 |
| BP | Regulation of leukocyte migration | 28/738 | 1.78E-08 | 1.09E-06 |
| BP | Lymphocyte activation involved in immune response | 26/738 | 1.98E-08 | 1.18E-06 |
| BP | Antigen receptor-mediated signaling pathway | 36/738 | 1.98E-08 | 1.18E-06 |
| BP | T cell costimulation | 14/738 | 2.19E-08 | 1.28E-06 |
| BP | Regulation of leukocyte mediated cytotoxicity | 16/738 | 2.53E-08 | 1.46E-06 |
| BP | Lymphocyte migration | 20/738 | 2.60E-08 | 1.46E-06 |
| BP | Positive regulation of lymphocyte mediated immunity | 19/738 | 2.61E-08 | 1.46E-06 |
| BP | Natural killer cell mediated immunity | 15/738 | 2.79E-08 | 1.55E-06 |
| BP | Defense response to virus | 31/738 | 2.97E-08 | 1.62E-06 |
| BP | Lymphocyte costimulation | 14/738 | 3.57E-08 | 1.92E-06 |
| BP | Regulation of production of molecular mediator of immune response | 22/738 | 4.38E-08 | 2.33E-06 |
| BP | Positive regulation of production of molecular mediator of immune response | 18/738 | 5.66E-08 | 2.97E-06 |
| BP | Negative regulation of leukocyte activation | 25/738 | 5.88E-08 | 3.05E-06 |
| BP | Positive regulation of secretion | 36/738 | 6.32E-08 | 3.24E-06 |
| BP | Positive regulation of T cell proliferation | 18/738 | 6.64E-08 | 3.36E-06 |
| BP | Positive regulation of secretion by cell | 34/738 | 7.92E-08 | 3.96E-06 |
| BP | Regulation of natural killer cell mediated immunity | 12/738 | 1.03E-07 | 5.08E-06 |
| BP | Positive regulation of adaptive immune response based on somatic recombination of immune receptors built from immunoglobulin superfamily domains | 18/738 | 1.23E-07 | 6.01E-06 |
| BP | Interleukin-15-mediated signaling pathway | 7/738 | 1.90E-07 | 9.04E-06 |
| BP | Cellular response to interleukin-15 | 7/738 | 1.90E-07 | 9.04E-06 |
| BP | Leukocyte mediated cytotoxicity | 18/738 | 1.92E-07 | 9.04E-06 |
| BP | Peptidyl-tyrosine phosphorylation | 37/738 | 2.35E-07 | 1.10E-05 |
| BP | Positive regulation of adaptive immune response | 18/738 | 2.55E-07 | 1.18E-05 |
| BP | Negative regulation of immune response | 22/738 | 2.64E-07 | 1.20E-05 |
| BP | Peptidyl-tyrosine modification | 37/738 | 2.87E-07 | 1.28E-05 |
| BP | Regulation of inflammatory response | 40/738 | 2.88E-07 | 1.28E-05 |
| BP | CD4-positive, alpha-beta T cell differentiation | 15/738 | 2.94E-07 | 1.29E-05 |
| BP | Regulation of natural killer cell mediated cytotoxicity | 11/738 | 3.29E-07 | 1.43E-05 |
| BP | Response to interleukin-15 | 7/738 | 3.68E-07 | 1.59E-05 |
| BP | Regulation of cell killing | 16/738 | 5.02E-07 | 2.14E-05 |
| BP | Natural killer cell mediated cytotoxicity | 13/738 | 5.23E-07 | 2.21E-05 |
| BP | T cell mediated immunity | 17/738 | 5.56E-07 | 2.31E-05 |
| BP | T cell cytokine production | 11/738 | 5.58E-07 | 2.31E-05 |
| BP | Positive regulation of leukocyte mediated cytotoxicity | 12/738 | 5.74E-07 | 2.35E-05 |
| BP | Negative regulation of cell activation | 25/738 | 5.82E-07 | 2.36E-05 |
| BP | Negative regulation of lymphocyte activation | 21/738 | 6.28E-07 | 2.52E-05 |
| BP | Response to mechanical stimulus | 25/738 | 6.98E-07 | 2.78E-05 |
| BP | Cellular response to interferon-gamma | 23/738 | 7.36E-07 | 2.90E-05 |
| BP | Negative regulation of cytokine production | 35/738 | 7.51E-07 | 2.93E-05 |
| BP | Regulation of alpha-beta T cell differentiation | 13/738 | 9.39E-07 | 3.63E-05 |
| BP | Positive regulation of innate immune response | 25/738 | 9.97E-07 | 3.82E-05 |
| BP | Positive regulation of natural killer cell mediated immunity | 9/738 | 1.01E-06 | 3.84E-05 |
| BP | Response to interferon-gamma | 24/738 | 1.31E-06 | 4.93E-05 |
| BP | Response to type I interferon | 16/738 | 1.40E-06 | 5.20E-05 |
| BP | Regulation of leukocyte chemotaxis | 18/738 | 1.57E-06 | 5.81E-05 |
| BP | T cell migration | 13/738 | 1.63E-06 | 5.96E-05 |
| BP | Regulation of CD4-positive, alpha-beta T cell differentiation | 11/738 | 1.83E-06 | 6.65E-05 |
| BP | Cell chemotaxis | 31/738 | 1.90E-06 | 6.83E-05 |
| BP | Positive regulation of response to biotic stimulus | 27/738 | 2.06E-06 | 7.35E-05 |
| BP | Positive regulation of natural killer cell mediated cytotoxicity | 8/738 | 2.22E-06 | 7.80E-05 |
| BP | Regulation of I-kappab kinase/NF-kappab signaling | 27/738 | 2.23E-06 | 7.80E-05 |
| BP | Interferon-gamma-mediated signaling pathway | 15/738 | 2.30E-06 | 7.98E-05 |
| BP | I-kappab kinase/NF-kappab signaling | 29/738 | 2.89E-06 | 9.94E-05 |
| BP | Interleukin-6 production | 21/738 | 3.20E-06 | 0.000109 |
| BP | Positive regulation of cell killing | 12/738 | 3.51E-06 | 0.000118 |
| BP | Mast cell activation | 12/738 | 3.51E-06 | 0.000118 |
| BP | Type I interferon signaling pathway | 15/738 | 4.00E-06 | 0.000132 |
| BP | Cellular response to type I interferon | 15/738 | 4.00E-06 | 0.000132 |
| BP | Regulation of B cell proliferation | 12/738 | 4.20E-06 | 0.000138 |
| BP | Regulation of T cell mediated immunity | 13/738 | 4.46E-06 | 0.000145 |
| BP | Leukocyte chemotaxis | 25/738 | 4.74E-06 | 0.000153 |
| BP | Regulation of lymphocyte migration | 12/738 | 5.94E-06 | 0.00019 |
| BP | Regulation of T-helper 1 type immune response | 8/738 | 6.05E-06 | 0.000191 |
| BP | CD8-positive, alpha-beta T cell activation | 8/738 | 6.05E-06 | 0.000191 |
| BP | Receptor signaling pathway via JAK-STAT | 20/738 | 6.65E-06 | 0.000208 |
| BP | Positive regulation of supramolecular fiber organization | 23/738 | 7.36E-06 | 0.000227 |
| BP | Alpha-beta T cell proliferation | 9/738 | 7.44E-06 | 0.000227 |
| BP | Positive regulation of CD4-positive, alpha-beta T cell activation | 9/738 | 7.44E-06 | 0.000227 |
| BP | Regulation of type I interferon production | 17/738 | 7.53E-06 | 0.000228 |
| BP | Phagocytosis | 34/738 | 7.59E-06 | 0.000229 |
| BP | Regulation of calcium-mediated signaling | 15/738 | 7.66E-06 | 0.000229 |
| BP | Positive regulation of myeloid cell differentiation | 15/738 | 8.68E-06 | 0.000258 |
| BP | Type I interferon production | 17/738 | 9.34E-06 | 0.000275 |
| BP | Regulation of defense response to virus | 13/738 | 9.52E-06 | 0.000279 |
| BP | Production of molecular mediator of immune response | 29/738 | 1.08E-05 | 0.000314 |
| BP | Macrophage differentiation | 10/738 | 1.10E-05 | 0.000315 |
| BP | Positive regulation of alpha-beta T cell differentiation | 10/738 | 1.10E-05 | 0.000315 |
| BP | Positive regulation of leukocyte migration | 18/738 | 1.11E-05 | 0.000316 |
| BP | Regulation of chemotaxis | 24/738 | 1.17E-05 | 0.000332 |
| BP | Regulation of antigen receptor-mediated signaling pathway | 12/738 | 1.33E-05 | 0.000374 |
| BP | Negative regulation of leukocyte differentiation | 15/738 | 1.40E-05 | 0.000392 |
| BP | Regulation of myeloid cell differentiation | 26/738 | 1.46E-05 | 0.000402 |
| BP | Regulation of peptidyl-tyrosine phosphorylation | 26/738 | 1.46E-05 | 0.000402 |
| BP | Regulation of interleukin-6 production | 19/738 | 1.51E-05 | 0.000412 |
| BP | Receptor signaling pathway via STAT | 20/738 | 1.62E-05 | 0.000439 |
| BP | CD4-positive, alpha-beta T cell cytokine production | 7/738 | 1.93E-05 | 0.000518 |
| BP | Response to interleukin-9 | 5/738 | 1.93E-05 | 0.000518 |
| BP | Positive regulation of leukocyte chemotaxis | 14/738 | 2.13E-05 | 0.000566 |
| BP | Response to lipopolysaccharide | 30/738 | 2.17E-05 | 0.00057 |
| BP | Negative regulation of defense response | 26/738 | 2.17E-05 | 0.00057 |
| BP | Regulation of protein secretion | 31/738 | 2.33E-05 | 0.000609 |
| BP | Regulation of T cell cytokine production | 8/738 | 2.41E-05 | 0.000625 |
| BP | Cellular calcium ion homeostasis | 37/738 | 2.42E-05 | 0.000625 |
| BP | Regulation of actin filament-based process | 34/738 | 2.58E-05 | 0.000661 |
| BP | Homeostasis of number of cells | 25/738 | 2.61E-05 | 0.000665 |
| BP | Regulation of neuron death | 29/738 | 2.66E-05 | 0.000673 |
| BP | Osteoclast differentiation | 14/738 | 2.70E-05 | 0.000677 |
| BP | Regulation of B cell activation | 21/738 | 2.71E-05 | 0.000677 |
| BP | Response to molecule of bacterial origin | 31/738 | 2.90E-05 | 0.000721 |
| BP | Interleukin-27-mediated signaling pathway | 5/738 | 3.43E-05 | 0.000837 |
| BP | Interleukin-35-mediated signaling pathway | 5/738 | 3.43E-05 | 0.000837 |
| BP | Synapse pruning | 5/738 | 3.43E-05 | 0.000837 |
| BP | Positive regulation of leukocyte degranulation | 7/738 | 3.53E-05 | 0.000857 |
| BP | Neuron death | 31/738 | 3.59E-05 | 0.000866 |
| BP | Positive regulation of interleukin-6 production | 13/738 | 3.62E-05 | 0.000868 |
| BP | Positive regulation of cytoskeleton organization | 23/738 | 3.75E-05 | 0.000894 |
| BP | Positive regulation of actin filament polymerization | 14/738 | 3.81E-05 | 0.000897 |
| BP | Macrophage activation | 14/738 | 3.81E-05 | 0.000897 |
| BP | Positive regulation of myeloid leukocyte mediated immunity | 8/738 | 3.88E-05 | 0.000898 |
| BP | Regulation of metal ion transport | 33/738 | 3.92E-05 | 0.000898 |
| BP | Receptor-mediated endocytosis | 29/738 | 3.95E-05 | 0.000898 |
| BP | B cell activation | 29/738 | 3.95E-05 | 0.000898 |
| BP | Regulation of reactive oxygen species metabolic process | 21/738 | 3.95E-05 | 0.000898 |
| BP | Lymphocyte chemotaxis | 11/738 | 3.96E-05 | 0.000898 |
| BP | Interleukin-2 production | 10/738 | 3.97E-05 | 0.000898 |
| BP | Response to drug | 33/738 | 4.12E-05 | 0.000927 |
| BP | Calcium ion homeostasis | 37/738 | 4.23E-05 | 0.000937 |
| BP | Regulation of mast cell activation | 9/738 | 4.23E-05 | 0.000937 |
| BP | Regulation of T cell migration | 9/738 | 4.23E-05 | 0.000937 |
| BP | Regulation of CD8-positive, alpha-beta T cell activation | 6/738 | 4.35E-05 | 0.000959 |
| BP | Neutrophil degranulation | 38/738 | 4.44E-05 | 0.000972 |
| BP | Actin filament organization | 35/738 | 4.53E-05 | 0.000988 |
| BP | Positive regulation of inflammatory response | 18/738 | 4.77E-05 | 0.001035 |
| BP | Regulation of ion transmembrane transport | 38/738 | 4.84E-05 | 0.001045 |
| BP | Positive regulation of chemotaxis | 17/738 | 4.89E-05 | 0.001049 |
| BP | Positive regulation of I-kappab kinase/NF-kappab signaling | 20/738 | 4.98E-05 | 0.001063 |
| BP | Neutrophil activation involved in immune response | 38/738 | 5.06E-05 | 0.001073 |
| BP | Myoblast fusion | 9/738 | 5.11E-05 | 0.001073 |
| BP | Positive regulation of chemokine production | 9/738 | 5.11E-05 | 0.001073 |
| BP | Protein polymerization | 27/738 | 5.33E-05 | 0.001114 |
| BP | Cellular response to mechanical stimulus | 12/738 | 5.42E-05 | 0.001128 |
| BP | Positive regulation of regulated secretory pathway | 10/738 | 5.50E-05 | 0.001139 |
| BP | Interleukin-2-mediated signaling pathway | 5/738 | 5.69E-05 | 0.001172 |
| BP | Regulation of lymphocyte chemotaxis | 7/738 | 6.10E-05 | 0.001244 |
| BP | Regulation of chemokine production | 11/738 | 6.10E-05 | 0.001244 |
| BP | Myeloid leukocyte migration | 22/738 | 6.30E-05 | 0.001278 |
| BP | Positive regulation of gtpase activity | 33/738 | 6.38E-05 | 0.001288 |
| BP | Protein secretion | 36/738 | 7.20E-05 | 0.001446 |
| BP | Positive regulation of lipid localization | 13/738 | 7.33E-05 | 0.001465 |
| BP | Syncytium formation by plasma membrane fusion | 10/738 | 7.51E-05 | 0.001482 |
| BP | Cell-cell fusion | 10/738 | 7.51E-05 | 0.001482 |
| BP | Establishment of protein localization to extracellular region | 36/738 | 7.52E-05 | 0.001482 |
| BP | Negative regulation of transport | 37/738 | 8.17E-05 | 0.001601 |
| BP | Positive regulation of T cell cytokine production | 6/738 | 8.50E-05 | 0.001651 |
| BP | Positive regulation of natural killer cell activation | 6/738 | 8.50E-05 | 0.001651 |
| BP | Regulation of actin cytoskeleton organization | 30/738 | 8.69E-05 | 0.001667 |
| BP | Mast cell activation involved in immune response | 9/738 | 8.70E-05 | 0.001667 |
| BP | Negative regulation of adaptive immune response | 9/738 | 8.70E-05 | 0.001667 |
| BP | T cell activation involved in immune response | 14/738 | 8.93E-05 | 0.001688 |
| BP | Regulation of response to cytokine stimulus | 20/738 | 8.94E-05 | 0.001688 |
| BP | Cellular response to interleukin-2 | 5/738 | 8.95E-05 | 0.001688 |
| BP | Macrophage derived foam cell differentiation | 8/738 | 9.10E-05 | 0.001688 |
| BP | Monocyte differentiation | 8/738 | 9.10E-05 | 0.001688 |
| BP | Foam cell differentiation | 8/738 | 9.10E-05 | 0.001688 |
| BP | Mononuclear cell differentiation | 8/738 | 9.10E-05 | 0.001688 |
| BP | T cell differentiation involved in immune response | 11/738 | 9.17E-05 | 0.001692 |
| BP | Regulation of myeloid leukocyte differentiation | 15/738 | 9.24E-05 | 0.001697 |
| BP | Regulation of defense response to virus by virus | 7/738 | 0.0001 | 0.001831 |
| BP | Regulation of actin filament organization | 25/738 | 0.000101 | 0.001835 |
| BP | Syncytium formation | 10/738 | 0.000101 | 0.001835 |
| BP | Protein localization to extracellular region | 36/738 | 0.000102 | 0.001837 |
| BP | Mast cell mediated immunity | 9/738 | 0.000103 | 0.001842 |
| BP | Regulation of interleukin-2 production | 9/738 | 0.000103 | 0.001842 |
| BP | Regulation of peptide secretion | 31/738 | 0.000103 | 0.001843 |
| BP | Regulation of cytokine-mediated signaling pathway | 19/738 | 0.000106 | 0.001874 |
| BP | Extrinsic apoptotic signaling pathway | 22/738 | 0.000106 | 0.00188 |
| BP | Natural killer cell activation | 12/738 | 0.000112 | 0.001978 |
| BP | Response to metal ion | 30/738 | 0.000117 | 0.002045 |
| BP | Cellular divalent inorganic cation homeostasis | 37/738 | 0.000119 | 0.002075 |
| BP | Positive regulation of T cell mediated immunity | 9/738 | 0.000121 | 0.002105 |
| BP | Pattern recognition receptor signaling pathway | 20/738 | 0.000127 | 0.002188 |
| BP | Regulation of defense response to virus by host | 8/738 | 0.000134 | 0.0023 |
| BP | Negative regulation of innate immune response | 10/738 | 0.000135 | 0.002301 |
| BP | Response to interleukin-2 | 5/738 | 0.000135 | 0.002301 |
| BP | Regulation of receptor signaling pathway via JAK-STAT | 16/738 | 0.000141 | 0.002395 |
| BP | Cell killing | 18/738 | 0.000144 | 0.002437 |
| BP | Chemokine production | 11/738 | 0.000152 | 0.002566 |
| BP | Negative regulation of hemopoiesis | 17/738 | 0.000156 | 0.002618 |
| BP | Calcium ion transport | 33/738 | 0.000159 | 0.002656 |
| BP | Positive regulation of B cell proliferation | 8/738 | 0.00016 | 0.002659 |
| BP | Regulation of calcium ion transport | 23/738 | 0.000161 | 0.002659 |
| BP | Regulation of gtpase activity | 36/738 | 0.000161 | 0.002659 |
| BP | Regulation of supramolecular fiber organization | 30/738 | 0.000163 | 0.002683 |
| BP | Actin polymerization or depolymerization | 21/738 | 0.000166 | 0.002722 |
| BP | Positive regulation of cytosolic calcium ion concentration | 27/738 | 0.000174 | 0.002848 |
| BP | Tyrosine phosphorylation of STAT protein | 12/738 | 0.000176 | 0.002862 |
| BP | Myotube differentiation | 14/738 | 0.000192 | 0.003101 |
| BP | Response to interferon-beta | 7/738 | 0.000195 | 0.003101 |
| BP | Negative regulation of B cell activation | 7/738 | 0.000195 | 0.003101 |
| BP | Cellular response to interleukin-4 | 7/738 | 0.000195 | 0.003101 |
| BP | T-helper 2 cell cytokine production | 5/738 | 0.000196 | 0.003101 |
| BP | CD8-positive, alpha-beta T cell differentiation | 5/738 | 0.000196 | 0.003101 |
| BP | Positive regulation of receptor signaling pathway via JAK-STAT | 12/738 | 0.000196 | 0.003101 |
| BP | Regulation of MAP kinase activity | 28/738 | 0.000199 | 0.003132 |
| BP | Cellular response to lipopolysaccharide | 20/738 | 0.000201 | 0.003157 |
| BP | Entry into host | 16/738 | 0.000211 | 0.003288 |
| BP | Regulation of extrinsic apoptotic signaling pathway | 17/738 | 0.000211 | 0.003288 |
| BP | Actin filament polymerization | 19/738 | 0.000212 | 0.003298 |
| BP | Regulation of T cell receptor signaling pathway | 8/738 | 0.000227 | 0.003512 |
| BP | Positive regulation of protein serine/threonine kinase activity | 28/738 | 0.00023 | 0.003536 |
| BP | Regulation of alpha-beta T cell proliferation | 7/738 | 0.000239 | 0.003668 |
| BP | Interaction with host | 20/738 | 0.000259 | 0.003962 |
| BP | Granulocyte migration | 16/738 | 0.000266 | 0.004055 |
| BP | Positive regulation of receptor signaling pathway via STAT | 12/738 | 0.000268 | 0.004055 |
| BP | Regulation of macrophage migration | 8/738 | 0.000268 | 0.004055 |
| BP | Lipoxygenase pathway | 5/738 | 0.000275 | 0.004149 |
| BP | Lipid localization | 33/738 | 0.000287 | 0.004295 |
| BP | Regulation of receptor signaling pathway via STAT | 16/738 | 0.000287 | 0.004295 |
| BP | Icosanoid metabolic process | 14/738 | 0.000299 | 0.004423 |
| BP | Divalent inorganic cation transport | 35/738 | 0.000299 | 0.004423 |
| BP | Positive regulation of MAP kinase activity | 23/738 | 0.000299 | 0.004423 |
| BP | Positive regulation of protein polymerization | 15/738 | 0.000309 | 0.004551 |
| BP | Negative regulation of synapse organization | 6/738 | 0.000329 | 0.004825 |
| BP | Response to ketone | 19/738 | 0.000335 | 0.004904 |
| BP | Movement in host environment | 17/738 | 0.000349 | 0.005089 |
| BP | Response to interleukin-4 | 7/738 | 0.000351 | 0.005108 |
| BP | Response to alcohol | 21/738 | 0.000363 | 0.005257 |
| BP | Regulation of myeloid leukocyte mediated immunity | 9/738 | 0.00039 | 0.005628 |
| BP | B cell proliferation | 12/738 | 0.000398 | 0.005699 |
| BP | Positive regulation of neuron death | 12/738 | 0.000398 | 0.005699 |
| BP | Regulation of cytosolic calcium ion concentration | 28/738 | 0.0004 | 0.005708 |
| BP | Positive regulation of lipid storage | 6/738 | 0.000413 | 0.005857 |
| BP | Positive regulation of interleukin-2 production | 6/738 | 0.000413 | 0.005857 |
| BP | Regulation of cell shape | 16/738 | 0.000415 | 0.005865 |
| BP | Reactive oxygen species metabolic process | 24/738 | 0.000429 | 0.005981 |
| BP | Negative regulation of type I interferon production | 8/738 | 0.000429 | 0.005981 |
| BP | Regulation of leukocyte degranulation | 8/738 | 0.000429 | 0.005981 |
| BP | Mast cell degranulation | 8/738 | 0.000429 | 0.005981 |
| BP | Cell redox homeostasis | 9/738 | 0.000445 | 0.006175 |
| BP | Divalent metal ion transport | 34/738 | 0.000468 | 0.006475 |
| BP | Cytokine secretion | 10/738 | 0.000472 | 0.006475 |
| BP | Calcium-mediated signaling | 20/738 | 0.000472 | 0.006475 |
| BP | Cellular response to molecule of bacterial origin | 20/738 | 0.000472 | 0.006475 |
| BP | Regulation of pattern recognition receptor signaling pathway | 12/738 | 0.00048 | 0.006562 |
| BP | Regulation of actin polymerization or depolymerization | 18/738 | 0.000492 | 0.006671 |
| BP | Negative regulation of secretion | 18/738 | 0.000492 | 0.006671 |
| BP | Regulation of interferon-beta production | 8/738 | 0.000498 | 0.006707 |
| BP | Regulation of extrinsic apoptotic signaling pathway in absence of ligand | 8/738 | 0.000498 | 0.006707 |
| BP | Modulation of chemical synaptic transmission | 33/738 | 0.0005 | 0.00672 |
| BP | T-helper cell differentiation | 9/738 | 0.000505 | 0.006764 |
| BP | Positive regulation of NF-kappab transcription factor activity | 16/738 | 0.000513 | 0.006834 |
| BP | Response to auditory stimulus | 6/738 | 0.000514 | 0.006834 |
| BP | Regulation of tyrosine phosphorylation of STAT protein | 11/738 | 0.000515 | 0.006834 |
| BP | Regulation of trans-synaptic signaling | 33/738 | 0.00052 | 0.00687 |
| BP | Regulation of actin filament length | 18/738 | 0.000523 | 0.006898 |
| BP | Negative regulation of viral genome replication | 9/738 | 0.000572 | 0.007484 |
| BP | Negative regulation of leukocyte migration | 8/738 | 0.000575 | 0.007484 |
| BP | T cell selection | 8/738 | 0.000575 | 0.007484 |
| BP | Regulation of calcium ion transport into cytosol | 12/738 | 0.000577 | 0.007484 |
| BP | Regulation of lipase activity | 12/738 | 0.000577 | 0.007484 |
| BP | Positive regulation of type I interferon production | 10/738 | 0.00059 | 0.007601 |
| BP | Regulation of toll-like receptor signaling pathway | 10/738 | 0.00059 | 0.007601 |
| BP | Negative regulation of interferon-gamma production | 7/738 | 0.000596 | 0.00763 |
| BP | Positive regulation of lymphocyte migration | 7/738 | 0.000596 | 0.00763 |
| BP | Alcohol metabolic process | 29/738 | 0.000612 | 0.007802 |
| BP | Regulation of cell-cell adhesion mediated by integrin | 4/738 | 0.000615 | 0.007802 |
| BP | Limb bud formation | 4/738 | 0.000615 | 0.007802 |
| BP | T cell chemotaxis | 6/738 | 0.000632 | 0.007998 |
| BP | Tissue remodeling | 17/738 | 0.000636 | 0.008019 |
| BP | CD4-positive, alpha-beta T cell differentiation involved in immune response | 9/738 | 0.000647 | 0.008128 |
| BP | Response to calcium ion | 15/738 | 0.000656 | 0.008157 |
| BP | Lysosome localization | 10/738 | 0.000657 | 0.008157 |
| BP | Signal transduction in absence of ligand | 10/738 | 0.000657 | 0.008157 |
| BP | Extrinsic apoptotic signaling pathway in absence of ligand | 10/738 | 0.000657 | 0.008157 |
| BP | Glial cell proliferation | 8/738 | 0.000661 | 0.008157 |
| BP | Interferon-beta production | 8/738 | 0.000661 | 0.008157 |
| BP | Positive regulation of macrophage derived foam cell differentiation | 5/738 | 0.000665 | 0.008158 |
| BP | Cell junction disassembly | 5/738 | 0.000665 | 0.008158 |
| BP | Cellular response to biotic stimulus | 21/738 | 0.000702 | 0.008536 |
| BP | Type 2 immune response | 7/738 | 0.000702 | 0.008536 |
| BP | Regulation of actin cytoskeleton reorganization | 7/738 | 0.000702 | 0.008536 |
| BP | Negative regulation of inflammatory response | 18/738 | 0.000711 | 0.008617 |
| BP | Unsaturated fatty acid metabolic process | 13/738 | 0.000718 | 0.008654 |
| BP | Negative regulation of T cell activation | 13/738 | 0.000718 | 0.008654 |
| BP | Alpha-beta T cell activation involved in immune response | 9/738 | 0.000729 | 0.008731 |
| BP | Alpha-beta T cell differentiation involved in immune response | 9/738 | 0.000729 | 0.008731 |
| BP | Negative regulation of viral process | 12/738 | 0.000752 | 0.008989 |
| BP | Negative regulation of myeloid leukocyte differentiation | 8/738 | 0.000757 | 0.009022 |
| BP | Positive regulation of defense response to virus by host | 6/738 | 0.000771 | 0.009108 |
| BP | Dendritic cell migration | 6/738 | 0.000771 | 0.009108 |
| BP | Regulation of B cell differentiation | 6/738 | 0.000771 | 0.009108 |
| BP | Viral entry into host cell | 14/738 | 0.000776 | 0.009143 |
| BP | Regulation of protein polymerization | 20/738 | 0.000781 | 0.009172 |
| BP | Neuroinflammatory response | 10/738 | 0.000811 | 0.009495 |
| BP | Actin cytoskeleton reorganization | 12/738 | 0.00082 | 0.009516 |
| BP | Regulation of anion transport | 12/738 | 0.00082 | 0.009516 |
| BP | Regulation of receptor-mediated endocytosis | 12/738 | 0.00082 | 0.009516 |
| BP | Negative regulation of secretion by cell | 16/738 | 0.000822 | 0.009516 |
| BP | Positive regulation of ion transport | 23/738 | 0.000829 | 0.009569 |
| BP | Positive regulation of exocytosis | 11/738 | 0.000836 | 0.009593 |
| BP | Bone remodeling | 11/738 | 0.000836 | 0.009593 |
| BP | Lipid transport | 29/738 | 0.000847 | 0.00969 |
| BP | Regulation of hormone secretion | 22/738 | 0.00085 | 0.009698 |
| BP | Chronic inflammatory response | 5/738 | 0.000858 | 0.009743 |
| BP | I-kappab phosphorylation | 5/738 | 0.000858 | 0.009743 |
| BP | Response to carbohydrate | 20/738 | 0.00087 | 0.009844 |
| BP | Response to macrophage colony-stimulating factor | 4/738 | 0.000894 | 0.010065 |
| BP | Cellular response to macrophage colony-stimulating factor stimulus | 4/738 | 0.000894 | 0.010065 |
| BP | NIK/NF-kappab signaling | 17/738 | 0.000924 | 0.010328 |
| BP | Regulation of macrophage chemotaxis | 6/738 | 0.000932 | 0.010328 |
| BP | TRIF-dependent toll-like receptor signaling pathway | 6/738 | 0.000932 | 0.010328 |
| BP | Positive regulation of CD4-positive, alpha-beta T cell differentiation | 6/738 | 0.000932 | 0.010328 |
| BP | Regulation of actin nucleation | 6/738 | 0.000932 | 0.010328 |
| BP | Regulation of syncytium formation by plasma membrane fusion | 6/738 | 0.000932 | 0.010328 |
| BP | Negative regulation of response to external stimulus | 31/738 | 0.000936 | 0.010344 |
| BP | Peptidyl-tyrosine autophosphorylation | 7/738 | 0.000959 | 0.010563 |
| BP | Negative regulation of leukocyte cell-cell adhesion | 14/738 | 0.000966 | 0.010591 |
| BP | Regulation of small gtpase mediated signal transduction | 25/738 | 0.000967 | 0.010591 |
| BP | Response to ethanol | 13/738 | 0.000983 | 0.010716 |
| BP | Neutrophil migration | 13/738 | 0.000983 | 0.010716 |
| BP | Maintenance of location | 25/738 | 0.00101 | 0.010976 |
| BP | Cellular response to peptide | 29/738 | 0.00103 | 0.01117 |
| BP | Myeloid cell homeostasis | 15/738 | 0.001061 | 0.011477 |
| BP | Positive regulation of alpha-beta T cell proliferation | 5/738 | 0.001091 | 0.011729 |
| BP | Positive regulation of lymphocyte chemotaxis | 5/738 | 0.001091 | 0.011729 |
| BP | Cellular response to external stimulus | 25/738 | 0.001101 | 0.011812 |
| BP | Negative regulation of antigen receptor-mediated signaling pathway | 6/738 | 0.001118 | 0.011967 |
| BP | Response to nutrient | 16/738 | 0.001129 | 0.01205 |
| BP | Negative regulation of protein secretion | 11/738 | 0.001198 | 0.012751 |
| BP | Peptidyl-serine phosphorylation | 24/738 | 0.001206 | 0.012801 |
| BP | Positive regulation of protein-containing complex assembly | 21/738 | 0.001225 | 0.012971 |
| BP | Auditory behavior | 4/738 | 0.001252 | 0.013185 |
| BP | Negative regulation of long-term synaptic potentiation | 4/738 | 0.001252 | 0.013185 |
| BP | Negative regulation of lymphocyte differentiation | 8/738 | 0.001263 | 0.013266 |
| BP | Positive regulation of phagocytosis | 9/738 | 0.001276 | 0.013372 |
| BP | Negative regulation of adaptive immune response based on somatic recombination of immune receptors built from immunoglobulin superfamily domains | 7/738 | 0.001285 | 0.013428 |
| BP | Cellular response to peptide hormone stimulus | 25/738 | 0.001306 | 0.013613 |
| BP | Fc receptor signaling pathway | 20/738 | 0.001315 | 0.013673 |
| BP | Negative regulation of calcium-mediated signaling | 6/738 | 0.001332 | 0.013815 |
| BP | Regulation of actin filament polymerization | 16/738 | 0.001356 | 0.013919 |
| BP | Activation of protein kinase activity | 25/738 | 0.001361 | 0.013919 |
| BP | Cellular response to abiotic stimulus | 25/738 | 0.001361 | 0.013919 |
| BP | Cellular response to environmental stimulus | 25/738 | 0.001361 | 0.013919 |
| BP | Inflammatory cell apoptotic process | 5/738 | 0.001366 | 0.013919 |
| BP | Positive regulation of mast cell activation | 5/738 | 0.001366 | 0.013919 |
| BP | Regulation of myoblast fusion | 5/738 | 0.001366 | 0.013919 |
| BP | Mammary gland development | 14/738 | 0.001369 | 0.013919 |
| BP | Calcium ion transport into cytosol | 15/738 | 0.001376 | 0.01396 |
| BP | Mononuclear cell migration | 11/738 | 0.001422 | 0.014322 |
| BP | Response to chemokine | 11/738 | 0.001422 | 0.014322 |
| BP | Cellular response to chemokine | 11/738 | 0.001422 | 0.014322 |
| BP | Hormone secretion | 24/738 | 0.001434 | 0.014406 |
| BP | Fatty acid derivative metabolic process | 16/738 | 0.00144 | 0.014429 |
| BP | Peptidyl-serine modification | 25/738 | 0.00148 | 0.014788 |
| BP | Response to peptide hormone | 31/738 | 0.001554 | 0.015495 |
| BP | Regulation of endocytosis | 18/738 | 0.001567 | 0.015591 |
| BP | Regulation of mast cell activation involved in immune response | 6/738 | 0.001574 | 0.015622 |
| BP | Secondary metabolic process | 8/738 | 0.0016 | 0.0158 |
| BP | Macrophage migration | 8/738 | 0.0016 | 0.0158 |
| BP | Regulation of macrophage differentiation | 5/738 | 0.00169 | 0.01661 |
| BP | Negative regulation of T cell receptor signaling pathway | 5/738 | 0.00169 | 0.01661 |
| BP | Negative regulation of interferon-beta production | 4/738 | 0.001698 | 0.01665 |
| BP | Mammary gland epithelium development | 9/738 | 0.001738 | 0.016999 |
| BP | Positive regulation of peptidyl-tyrosine phosphorylation | 17/738 | 0.001743 | 0.01701 |
| BP | Regulation of regulated secretory pathway | 15/738 | 0.001766 | 0.017189 |
| BP | Positive regulation of myeloid leukocyte differentiation | 8/738 | 0.001794 | 0.017418 |
| BP | Positive regulation of cell projection organization | 28/738 | 0.001804 | 0.017476 |
| BP | Receptor internalization | 12/738 | 0.001819 | 0.017581 |
| BP | Myd88-independent toll-like receptor signaling pathway | 6/738 | 0.001849 | 0.017791 |
| BP | Regulation of natural killer cell activation | 6/738 | 0.001849 | 0.017791 |
| BP | Leukocyte homeostasis | 10/738 | 0.001912 | 0.018264 |
| BP | Negative regulation of leukocyte proliferation | 10/738 | 0.001912 | 0.018264 |
| BP | Negative regulation of viral life cycle | 10/738 | 0.001912 | 0.018264 |
| BP | Negative regulation of T cell differentiation | 7/738 | 0.001928 | 0.018332 |
| BP | Activated T cell proliferation | 7/738 | 0.001928 | 0.018332 |
| BP | Fatty acid metabolic process | 28/738 | 0.001942 | 0.018417 |
| BP | Negative regulation of peptide secretion | 11/738 | 0.001975 | 0.018692 |
| BP | Viral life cycle | 25/738 | 0.002044 | 0.019303 |
| BP | Receptor metabolic process | 17/738 | 0.002051 | 0.019326 |
| BP | Dendritic cell chemotaxis | 5/738 | 0.002067 | 0.019428 |
| BP | Hormone transport | 24/738 | 0.002088 | 0.019578 |
| BP | Cardiac ventricle morphogenesis | 9/738 | 0.002115 | 0.019784 |
| BP | Regulation of T cell mediated cytotoxicity | 6/738 | 0.002159 | 0.020066 |
| BP | Interleukin-4 production | 6/738 | 0.002159 | 0.020066 |
| BP | Regulatory T cell differentiation | 6/738 | 0.002159 | 0.020066 |
| BP | Response to antibiotic | 7/738 | 0.00219 | 0.020308 |
| BP | Cortical cytoskeleton organization | 8/738 | 0.002236 | 0.020533 |
| BP | Regulation of interleukin-10 production | 8/738 | 0.002236 | 0.020533 |
| BP | Glial cell activation | 8/738 | 0.002236 | 0.020533 |
| BP | Mechanosensory behavior | 4/738 | 0.002245 | 0.020533 |
| BP | Regulation of granulocyte differentiation | 4/738 | 0.002245 | 0.020533 |
| BP | Negative regulation of lymphocyte migration | 4/738 | 0.002245 | 0.020533 |
| BP | Chemokine-mediated signaling pathway | 10/738 | 0.002273 | 0.020747 |
| BP | Negative regulation of protein transport | 14/738 | 0.002446 | 0.022272 |
| BP | Regulation of cell adhesion mediated by integrin | 7/738 | 0.002479 | 0.02248 |
| BP | Positive regulation of antigen receptor-mediated signaling pathway | 5/738 | 0.002502 | 0.02248 |
| BP | Regulation of response to interferon-gamma | 5/738 | 0.002502 | 0.02248 |
| BP | Regulation of interferon-gamma-mediated signaling pathway | 5/738 | 0.002502 | 0.02248 |
| BP | Regulation of T-helper cell differentiation | 6/738 | 0.002507 | 0.02248 |
| BP | Negative regulation of signal transduction in absence of ligand | 6/738 | 0.002507 | 0.02248 |
| BP | Negative regulation of extrinsic apoptotic signaling pathway in absence of ligand | 6/738 | 0.002507 | 0.02248 |
| BP | Toll-like receptor signaling pathway | 14/738 | 0.0026 | 0.02326 |
| BP | Positive regulation of actin filament bundle assembly | 8/738 | 0.00276 | 0.024641 |
| BP | Regulation of cation transmembrane transport | 25/738 | 0.002784 | 0.024799 |
| BP | Erythrocyte differentiation | 12/738 | 0.002794 | 0.024818 |
| BP | Positive regulation of lipase activity | 9/738 | 0.002798 | 0.024818 |
| BP | Regulation of exocytosis | 18/738 | 0.002882 | 0.025349 |
| BP | Leukocyte migration involved in inflammatory response | 4/738 | 0.002901 | 0.025349 |
| BP | Positive regulation of type 2 immune response | 4/738 | 0.002901 | 0.025349 |
| BP | Positive regulation of mast cell activation involved in immune response | 4/738 | 0.002901 | 0.025349 |
| BP | Positive regulation of mast cell degranulation | 4/738 | 0.002901 | 0.025349 |
| BP | Negative regulation of natural killer cell mediated cytotoxicity | 4/738 | 0.002901 | 0.025349 |
| BP | Negative regulation of cytokine secretion | 4/738 | 0.002901 | 0.025349 |
| BP | Organic hydroxy compound biosynthetic process | 20/738 | 0.002931 | 0.025559 |
| BP | Cellular aldehyde metabolic process | 8/738 | 0.003056 | 0.026519 |
| BP | Interleukin-10 production | 8/738 | 0.003056 | 0.026519 |
| BP | Renal tubule morphogenesis | 9/738 | 0.003061 | 0.026519 |
| BP | Negative regulation of establishment of protein localization | 14/738 | 0.00311 | 0.026806 |
| BP | Response to decreased oxygen levels | 26/738 | 0.003115 | 0.026806 |
| BP | Regulation of reactive oxygen species biosynthetic process | 11/738 | 0.00312 | 0.026806 |
| BP | Negative regulation of extrinsic apoptotic signaling pathway | 11/738 | 0.00312 | 0.026806 |
| BP | Microglial cell activation | 7/738 | 0.003145 | 0.026847 |
| BP | Leukocyte activation involved in inflammatory response | 7/738 | 0.003145 | 0.026847 |
| BP | Endocytic recycling | 7/738 | 0.003145 | 0.026847 |
| BP | Regulation of protein-containing complex assembly | 30/738 | 0.003192 | 0.02716 |
| BP | Epidermal growth factor receptor signaling pathway | 12/738 | 0.0032 | 0.02716 |
| BP | Learning or memory | 20/738 | 0.003201 | 0.02716 |
| BP | Inflammatory response to antigenic stimulus | 8/738 | 0.003375 | 0.028516 |
| BP | Bone resorption | 8/738 | 0.003375 | 0.028516 |
| BP | Ossification | 28/738 | 0.003405 | 0.028665 |
| BP | Production of molecular mediator involved in inflammatory response | 10/738 | 0.00342 | 0.028665 |
| BP | Negative regulation of immune effector process | 12/738 | 0.003421 | 0.028665 |
| BP | Gland morphogenesis | 12/738 | 0.003421 | 0.028665 |
| BP | Positive regulation of cell morphogenesis involved in differentiation | 14/738 | 0.003494 | 0.029217 |
| BP | Sodium ion transmembrane transport | 15/738 | 0.003523 | 0.029356 |
| BP | Regulation of mononuclear cell migration | 7/738 | 0.003525 | 0.029356 |
| BP | Nitric oxide biosynthetic process | 9/738 | 0.003645 | 0.030142 |
| BP | Negative regulation of lymphocyte proliferation | 9/738 | 0.003645 | 0.030142 |
| BP | Negative regulation of natural killer cell mediated immunity | 4/738 | 0.003678 | 0.030142 |
| BP | Regulation of T cell chemotaxis | 4/738 | 0.003678 | 0.030142 |
| BP | Sequestering of triglyceride | 4/738 | 0.003678 | 0.030142 |
| BP | Regulation of mammary gland epithelial cell proliferation | 4/738 | 0.003678 | 0.030142 |
| BP | Cell-cell adhesion mediated by integrin | 4/738 | 0.003678 | 0.030142 |
| BP | Multi-organism cellular process | 4/738 | 0.003678 | 0.030142 |
| BP | Cortical actin cytoskeleton organization | 6/738 | 0.003803 | 0.031106 |
| BP | Monocarboxylic acid biosynthetic process | 19/738 | 0.003831 | 0.031221 |
| BP | Fc-gamma receptor signaling pathway | 13/738 | 0.003832 | 0.031221 |
| BP | Positive regulation of stress fiber assembly | 7/738 | 0.003938 | 0.032021 |
| BP | B cell activation involved in immune response | 9/738 | 0.003968 | 0.032075 |
| BP | Negative regulation of mononuclear cell proliferation | 9/738 | 0.003968 | 0.032075 |
| BP | Positive regulation of NIK/NF-kappab signaling | 9/738 | 0.003968 | 0.032075 |
| BP | Second-messenger-mediated signaling | 30/738 | 0.00399 | 0.032124 |
| BP | Renal tubule development | 10/738 | 0.00399 | 0.032124 |
| BP | Granulocyte chemotaxis | 12/738 | 0.004156 | 0.03334 |
| BP | Negative regulation of neuron death | 17/738 | 0.004157 | 0.03334 |
| BP | Positive regulation of vascular endothelial growth factor production | 5/738 | 0.004201 | 0.033558 |
| BP | Positive regulation of syncytium formation by plasma membrane fusion | 5/738 | 0.004201 | 0.033558 |
| BP | Lipid storage | 9/738 | 0.004313 | 0.034355 |
| BP | Organophosphate catabolic process | 13/738 | 0.004318 | 0.034355 |
| BP | Positive regulation of DNA replication | 6/738 | 0.004329 | 0.034379 |
| BP | Positive regulation of protein kinase B signaling | 15/738 | 0.004358 | 0.034446 |
| BP | Cytosolic calcium ion transport | 15/738 | 0.004358 | 0.034446 |
| BP | Regulation of symbiotic process | 18/738 | 0.004385 | 0.034446 |
| BP | Positive regulation of interleukin-1 beta production | 7/738 | 0.004388 | 0.034446 |
| BP | Negative regulation of I-kappab kinase/NF-kappab signaling | 7/738 | 0.004388 | 0.034446 |
| BP | Actin nucleation | 7/738 | 0.004388 | 0.034446 |
| BP | Regulation of cell morphogenesis | 32/738 | 0.004405 | 0.034517 |
| BP | Reactive oxygen species biosynthetic process | 12/738 | 0.004427 | 0.034624 |
| BP | Blood coagulation | 24/738 | 0.004488 | 0.034918 |
| BP | Cellular extravasation | 8/738 | 0.004491 | 0.034918 |
| BP | Regulation of osteoclast differentiation | 8/738 | 0.004491 | 0.034918 |
| BP | Fc receptor mediated stimulatory signaling pathway | 13/738 | 0.004578 | 0.035531 |
| BP | Regulation of phagocytosis | 10/738 | 0.004633 | 0.035892 |
| BP | Erythrocyte homeostasis | 12/738 | 0.004713 | 0.03644 |
| BP | Regulation of cellular component size | 26/738 | 0.004733 | 0.036526 |
| BP | Cellular defense response | 7/738 | 0.004875 | 0.037469 |
| BP | Myeloid dendritic cell activation | 5/738 | 0.004916 | 0.037469 |
| BP | Regulation of myeloid cell apoptotic process | 5/738 | 0.004916 | 0.037469 |
| BP | Negative regulation of osteoclast differentiation | 5/738 | 0.004916 | 0.037469 |
| BP | Regulation of B cell receptor signaling pathway | 5/738 | 0.004916 | 0.037469 |
| BP | Cytoplasmic pattern recognition receptor signaling pathway | 8/738 | 0.004919 | 0.037469 |
| BP | Regulation of nitric oxide biosynthetic process | 8/738 | 0.004919 | 0.037469 |
| BP | Epithelial cell migration | 25/738 | 0.004955 | 0.037674 |
| BP | Negative regulation of response to biotic stimulus | 10/738 | 0.004984 | 0.037752 |
| BP | Vascular endothelial growth factor receptor signaling pathway | 10/738 | 0.004984 | 0.037752 |
| BP | Cell cycle arrest | 18/738 | 0.00501 | 0.037881 |
| BP | Regulation of insulin secretion | 15/738 | 0.005084 | 0.03837 |
| BP | Positive regulation of neuron projection development | 21/738 | 0.005158 | 0.038852 |
| BP | Positive regulation of neurogenesis | 31/738 | 0.005258 | 0.039538 |
| BP | Carboxylic acid biosynthetic process | 25/738 | 0.005306 | 0.039826 |
| BP | Positive regulation of hormone secretion | 12/738 | 0.005328 | 0.039916 |
| BP | Hemostasis | 24/738 | 0.00536 | 0.040084 |
| BP | Organic acid biosynthetic process | 25/738 | 0.005489 | 0.040834 |
| BP | Epithelium migration | 25/738 | 0.005489 | 0.040834 |
| BP | Nitric oxide metabolic process | 9/738 | 0.005491 | 0.040834 |
| BP | Macrophage chemotaxis | 6/738 | 0.005539 | 0.041046 |
| BP | Regulation of neutrophil migration | 6/738 | 0.005539 | 0.041046 |
| BP | Coagulation | 24/738 | 0.005551 | 0.041057 |
| BP | Positive regulation of macrophage chemotaxis | 4/738 | 0.005629 | 0.041412 |
| BP | T-helper 1 cell differentiation | 4/738 | 0.005629 | 0.041412 |
| BP | Gamma-delta T cell activation | 4/738 | 0.005629 | 0.041412 |
| BP | Negative regulation of biomineral tissue development | 5/738 | 0.005712 | 0.041775 |
| BP | Negative regulation of biomineralization | 5/738 | 0.005712 | 0.041775 |
| BP | Tumor necrosis factor production | 11/738 | 0.00578 | 0.041775 |
| BP | T cell activation via T cell receptor contact with antigen bound to MHC molecule on antigen presenting cell | 3/738 | 0.005821 | 0.041775 |
| BP | Hypersensitivity | 3/738 | 0.005821 | 0.041775 |
| BP | Regulation of T cell tolerance induction | 3/738 | 0.005821 | 0.041775 |
| BP | Regulation of chronic inflammatory response | 3/738 | 0.005821 | 0.041775 |
| BP | Cardiac ventricle formation | 3/738 | 0.005821 | 0.041775 |
| BP | Regulation of toll-like receptor 9 signaling pathway | 3/738 | 0.005821 | 0.041775 |
| BP | Protein folding in endoplasmic reticulum | 3/738 | 0.005821 | 0.041775 |
| BP | Glial cell-derived neurotrophic factor receptor signaling pathway | 3/738 | 0.005821 | 0.041775 |
| BP | Regulation of T-helper 1 cell differentiation | 3/738 | 0.005821 | 0.041775 |
| BP | Positive regulation of megakaryocyte differentiation | 3/738 | 0.005821 | 0.041775 |
| BP | Positive regulation of B cell receptor signaling pathway | 3/738 | 0.005821 | 0.041775 |
| BP | Positive regulation of animal organ morphogenesis | 9/738 | 0.005934 | 0.042474 |
| BP | Regulation of B cell mediated immunity | 7/738 | 0.00597 | 0.042474 |
| BP | Regulation of immunoglobulin mediated immune response | 7/738 | 0.00597 | 0.042474 |
| BP | Ruffle organization | 7/738 | 0.00597 | 0.042474 |
| BP | Negative regulation of peptidyl-tyrosine phosphorylation | 7/738 | 0.00597 | 0.042474 |
| BP | Steroid metabolic process | 23/738 | 0.006 | 0.042613 |
| BP | Positive regulation of calcium-mediated signaling | 6/738 | 0.006229 | 0.044162 |
| BP | Response to transforming growth factor beta | 19/738 | 0.006358 | 0.044997 |
| BP | Cellular response to drug | 8/738 | 0.006394 | 0.045011 |
| BP | Positive regulation of tyrosine phosphorylation of STAT protein | 8/738 | 0.006394 | 0.045011 |
| BP | Positive regulation of Ras protein signal transduction | 8/738 | 0.006394 | 0.045011 |
| BP | Homotypic cell-cell adhesion | 9/738 | 0.006404 | 0.045011 |
| BP | Sterol transport | 11/738 | 0.006562 | 0.046044 |
| BP | Positive regulation of response to cytokine stimulus | 7/738 | 0.006582 | 0.046044 |
| BP | Regulation of type 2 immune response | 5/738 | 0.006596 | 0.046044 |
| BP | Positive regulation of T cell migration | 5/738 | 0.006596 | 0.046044 |
| BP | Tissue migration | 25/738 | 0.006702 | 0.046708 |
| BP | Negative regulation of leukocyte mediated cytotoxicity | 4/738 | 0.006822 | 0.047061 |
| BP | Negative regulation of leukocyte chemotaxis | 4/738 | 0.006822 | 0.047061 |
| BP | Positive regulation of T-helper 1 type immune response | 4/738 | 0.006822 | 0.047061 |
| BP | Cardiac right ventricle morphogenesis | 4/738 | 0.006822 | 0.047061 |
| BP | Regulation of Arp2/3 complex-mediated actin nucleation | 4/738 | 0.006822 | 0.047061 |
| BP | Positive regulation of myoblast fusion | 4/738 | 0.006822 | 0.047061 |
| BP | Reactive nitrogen species metabolic process | 9/738 | 0.006902 | 0.047534 |
| BP | T cell mediated cytotoxicity | 6/738 | 0.006979 | 0.04785 |
| BP | Tumor necrosis factor superfamily cytokine production | 11/738 | 0.006983 | 0.04785 |
| BP | Regulation of NIK/NF-kappab signaling | 11/738 | 0.006983 | 0.04785 |
| BP | Phospholipid metabolic process | 29/738 | 0.007067 | 0.048348 |
| BP | Response to oxygen levels | 26/738 | 0.007225 | 0.049348 |
| CC | External side of plasma membrane | 50/761 | 1.49E-12 | 6.44E-10 |
| CC | Membrane raft | 30/761 | 1.48E-05 | 0.002106 |
| CC | Membrane microdomain | 30/761 | 1.57E-05 | 0.002106 |
| CC | Secretory granule membrane | 28/761 | 2.65E-05 | 0.002106 |
| CC | Early endosome | 32/761 | 3.25E-05 | 0.002106 |
| CC | Membrane region | 30/761 | 3.26E-05 | 0.002106 |
| CC | MHC protein complex | 7/761 | 3.42E-05 | 0.002106 |
| CC | Endocytic vesicle | 27/761 | 0.000101 | 0.005427 |
| CC | Intrinsic component of synaptic vesicle membrane | 8/761 | 0.000414 | 0.019869 |
| CC | Transport vesicle | 30/761 | 0.000734 | 0.031672 |
| CC | Extrinsic component of membrane | 24/761 | 0.000941 | 0.036938 |
| CC | Phagocytic vesicle | 14/761 | 0.001136 | 0.039518 |
| CC | Exocytic vesicle | 19/761 | 0.00119 | 0.039518 |
| CC | Mast cell granule | 5/761 | 0.001334 | 0.041135 |
| CC | Specific granule | 15/761 | 0.001484 | 0.042686 |
| CC | Extrinsic component of cytoplasmic side of plasma membrane | 11/761 | 0.001612 | 0.043056 |
| CC | Integral component of synaptic vesicle membrane | 6/761 | 0.0018 | 0.043056 |
| CC | Cytoplasmic side of plasma membrane | 15/761 | 0.001896 | 0.043056 |
| CC | Tertiary granule | 15/761 | 0.001896 | 0.043056 |
| CC | Lamellipodium | 17/761 | 0.002279 | 0.049169 |
| CC | Ficolin-1-rich granule membrane | 8/761 | 0.002408 | 0.049478 |
| MF | Immune receptor activity | 33/754 | 5.46E-17 | 4.21E-14 |
| MF | Cytokine receptor activity | 24/754 | 7.57E-13 | 2.92E-10 |
| MF | SH3 domain binding | 22/754 | 1.78E-08 | 4.57E-06 |
| MF | Cytokine receptor binding | 32/754 | 8.30E-08 | 1.60E-05 |
| MF | Cytokine binding | 20/754 | 6.45E-07 | 9.95E-05 |
| MF | MHC protein complex binding | 8/754 | 4.54E-06 | 0.000584 |
| MF | Cytokine activity | 25/754 | 1.37E-05 | 0.001512 |
| MF | Receptor ligand activity | 40/754 | 2.63E-05 | 0.002281 |
| MF | Tumor necrosis factor receptor binding | 8/754 | 2.66E-05 | 0.002281 |
| MF | Signaling receptor activator activity | 40/754 | 3.31E-05 | 0.002556 |
| MF | MHC protein binding | 9/754 | 4.19E-05 | 0.002937 |
| MF | Growth factor receptor binding | 17/754 | 6.83E-05 | 0.004392 |
| MF | Small gtpase binding | 35/754 | 9.05E-05 | 0.00537 |
| MF | Nucleoside-triphosphatase regulator activity | 30/754 | 0.000112 | 0.006188 |
| MF | Protein tyrosine kinase activity | 16/754 | 0.000136 | 0.006984 |
| MF | Gtpase regulator activity | 27/754 | 0.000176 | 0.008226 |
| MF | Gtpase activator activity | 25/754 | 0.000181 | 0.008226 |
| MF | Ras gtpase binding | 33/754 | 0.000239 | 0.010241 |
| MF | Steroid dehydrogenase activity | 7/754 | 0.000392 | 0.015899 |
| MF | Tumor necrosis factor receptor superfamily binding | 8/754 | 0.000687 | 0.026504 |
| MF | Ras guanyl-nucleotide exchange factor activity | 13/754 | 0.000883 | 0.032427 |
| MF | Rho gtpase binding | 16/754 | 0.001057 | 0.037086 |
| MF | Steroid dehydrogenase activity, acting on the CH-OH group of donors, NAD or NADP as acceptor | 6/754 | 0.001202 | 0.040324 |
| MF | Steroid binding | 12/754 | 0.001357 | 0.043637 |
| MF | Peptide disulfide oxidoreductase activity | 4/754 | 0.001501 | 0.04634 |

**Supplementary Table S3 Functional annotation for m^6^A-related genes (Kyoto Encyclopedia of Genes and Genomes-Biological process)**

| Description | | GeneRatio | pvalue | qvalue |
| --- | --- | --- | --- | --- |
| Cytokine-cytokine receptor interaction | 50/392 | 2.43E-15 | 5.61E-13 |  |
| Osteoclast differentiation | | 30/392 | 2.29E-13 | 2.63E-11 |
| Th17 cell differentiation | | 25/392 | 2.62E-11 | 2.01E-09 |
| JAK-STAT signaling pathway | | 30/392 | 1.34E-10 | 7.75E-09 |
| Th1 and Th2 cell differentiation | | 21/392 | 1.68E-09 | 7.73E-08 |
| Natural killer cell mediated cytotoxicity | | 25/392 | 2.56E-09 | 9.82E-08 |
| Human T-cell leukemia virus 1 infection | | 30/392 | 1.94E-07 | 6.39E-06 |
| Viral protein interaction with cytokine and cytokine receptor | | 19/392 | 2.34E-07 | 6.74E-06 |
| B cell receptor signaling pathway | | 17/392 | 2.75E-07 | 6.78E-06 |
| Inflammatory bowel disease | | 15/392 | 3.20E-07 | 6.78E-06 |
| Chagas disease | | 19/392 | 3.24E-07 | 6.78E-06 |
| Chemokine signaling pathway | | 27/392 | 4.75E-07 | 9.13E-06 |
| Leishmaniasis | | 16/392 | 5.99E-07 | 1.06E-05 |
| Epstein-Barr virus infection | | 27/392 | 1.32E-06 | 2.17E-05 |
| Influenza A | | 24/392 | 2.40E-06 | 3.69E-05 |
| Hematopoietic cell lineage | | 17/392 | 4.38E-06 | 6.32E-05 |
| Human cytomegalovirus infection | | 27/392 | 1.05E-05 | 0.000142 |
| Fc gamma R-mediated phagocytosis | | 16/392 | 1.43E-05 | 0.000183 |
| Acute myeloid leukemia | | 13/392 | 1.52E-05 | 0.000184 |
| PD-L1 expression and PD-1 checkpoint pathway in cancer | | 15/392 | 2.03E-05 | 0.000234 |
| Hepatitis C | | 20/392 | 6.39E-05 | 0.000702 |
| Human immunodeficiency virus 1 infection | | 24/392 | 8.42E-05 | 0.000882 |
| PI3K-Akt signaling pathway | | 34/392 | 9.09E-05 | 0.000911 |
| Viral myocarditis | | 11/392 | 0.000118 | 0.001113 |
| Toll-like receptor signaling pathway | | 15/392 | 0.00013 | 0.001113 |
| C-type lectin receptor signaling pathway | | 15/392 | 0.00013 | 0.001113 |
| T cell receptor signaling pathway | | 15/392 | 0.00013 | 0.001113 |
| Cell adhesion molecules | | 18/392 | 0.00029 | 0.002225 |
| Hepatitis B | | 19/392 | 0.000291 | 0.002225 |
| TNF signaling pathway | | 15/392 | 0.000301 | 0.002225 |
| Toxoplasmosis | | 15/392 | 0.000301 | 0.002225 |
| Yersinia infection | | 17/392 | 0.000309 | 0.002225 |
| Measles | | 17/392 | 0.000367 | 0.002518 |
| Phagosome | | 18/392 | 0.000371 | 0.002518 |
| Tuberculosis | | 20/392 | 0.000417 | 0.002749 |
| NF-kappa B signaling pathway | | 14/392 | 0.000451 | 0.002888 |
| Staphylococcus aureus infection | | 13/392 | 0.000676 | 0.00421 |
| Graft-versus-host disease | | 8/392 | 0.000772 | 0.004685 |
| Type I diabetes mellitus | | 8/392 | 0.00091 | 0.005376 |
| Pertussis | | 11/392 | 0.000987 | 0.005592 |
| AGE-RAGE signaling pathway in diabetic complications | | 13/392 | 0.000999 | 0.005592 |
| Kaposi sarcoma-associated herpesvirus infection | | 20/392 | 0.001019 | 0.005592 |
| Rap1 signaling pathway | | 21/392 | 0.001216 | 0.00652 |
| Fc epsilon RI signaling pathway | | 10/392 | 0.001468 | 0.007689 |
| Rheumatoid arthritis | | 12/392 | 0.001653 | 0.008466 |
| Parathyroid hormone synthesis, secretion and action | | 13/392 | 0.001721 | 0.008623 |
| Viral carcinogenesis | | 20/392 | 0.002004 | 0.009829 |
| MAPK signaling pathway | | 26/392 | 0.002049 | 0.00984 |
| NOD-like receptor signaling pathway | | 18/392 | 0.002831 | 0.01332 |
| Pancreatic cancer | | 10/392 | 0.003419 | 0.015454 |
| Chronic myeloid leukemia | | 10/392 | 0.003419 | 0.015454 |
| Amoebiasis | | 12/392 | 0.003627 | 0.01608 |
| Ras signaling pathway | | 21/392 | 0.004099 | 0.017827 |
| Human papillomavirus infection | | 27/392 | 0.005217 | 0.022272 |
| Adherens junction | | 9/392 | 0.006886 | 0.028862 |
| Colorectal cancer | | 10/392 | 0.008276 | 0.034069 |
| Focal adhesion | | 18/392 | 0.00845 | 0.034176 |
| Intestinal immune network for iga production | | 7/392 | 0.008692 | 0.034496 |
| Leukocyte transendothelial migration | | 12/392 | 0.008829 | 0.034496 |
| Allograft rejection | | 6/392 | 0.009132 | 0.035084 |
| Proteoglycans in cancer | | 18/392 | 0.010263 | 0.038786 |
| Foxo signaling pathway | | 13/392 | 0.010594 | 0.03939 |
| Bacterial invasion of epithelial cells | | 9/392 | 0.01158 | 0.042372 |
| Sphingolipid signaling pathway | | 12/392 | 0.01223 | 0.044051 |
| Antigen processing and presentation | | 9/392 | 0.012551 | 0.044511 |
| Autoimmune thyroid disease | | 7/392 | 0.01323 | 0.04621 |
